# Iridium-Catalyzed Reductive Nitro-Mannich Cyclization

**DOI:** 10.1002/chem.201405256

**Published:** 2014-11-14

**Authors:** Alex W Gregory, Alan Chambers, Alison Hawkins, Pavol Jakubec, Darren J Dixon

**Affiliations:** a Department of Chemistry, Chemistry Research Laboratory, University of Oxford Mansfield Road, Oxford OX1 3TA (UK)

**Keywords:** amide activation, domino reactions, iridium, Mannich reaction, reduction, silanes

## Abstract

A new chemoselective reductive nitro-Mannich cyclization reaction sequence of nitroalkyl-tethered lactams has been developed. Relying on the rapid and chemoselective iridium(I)-catalyzed reduction of lactams to the corresponding enamine, subsequent nitro-Mannich cyclization of tethered nitroalkyl functionality provides direct access to important alkaloid natural-product-like structures in yields up to 81 % and in diastereoselectivities that are typically good to excellent. An in-depth understanding of the reaction mechanism has been gained through NMR studies and characterization of reaction intermediates. The new methodology has been applied to the total synthesis of (±)-*epi*-epiquinamide in four steps.

Reaction cascades are becoming mainstream in organic synthesis, allowing the synthesis of advanced structures with fewer purification steps, increased speed and efficiency.^1^, ^2^ The development of new cascade sequences can be either methodology- or target-driven and in a few cases they can provide the critical link from a late stage intermediate to the end-game sequence in a total synthesis. To this end we recently reported an unprecedented chemoselective reductive nitro-Mannich cyclization of **1** proceeding via a putative iminium intermediate to form the **B** ring in manzamine A (**2**) (Scheme 1).^3^, ^4^

**Scheme 1:**
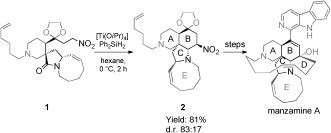
Reductive nitro-Mannich cascade in the total synthesis of manzamine A.^3^

Recognising the synthetic potential of this novel annulation strategy, we wanted to investigate whether an analogous reductive nitro-Mannich cyclization of *N*-linked lactam substrates of type **3** was feasible.^5^–^7^ Such chemistry would allow direct access to fused nitrogen-containing bicycles of type **5** via reactive iminium ion intermediates **4** (Scheme 2).^8^ These motifs are abundant in nature, making up major classes of alkaloid natural products which show important biological activity.^9^, ^10^ In addition, the presence of the versatile nitro group could be exploited as a handle to access, for example, ketone^11^ and amine functionality. As such the new methodology would be useful for natural product and library synthesis alike. Herein we wish to report our findings.

**Scheme 2:**
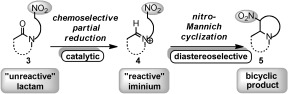
Proposed partial reductive nitro-Mannich cyclization concept.

Readily prepared caprolactam-derived substrate **3 a** was selected as a model system and initially subjected to the modified Buchwald conditions used in the synthesis of manzamine A.^12^, ^13^ Pleasingly we were able to isolate the desired bicycle **5 a** albeit in only 17 % yield (Table 1, entry 1). A range of typical hydridic reducing agents including DIBAL (diisobutylaluminiumhydride) and Schwartz reagent ([ZrCl(C_5_H_5_)_2_H]) were screened. Unfortunately, in all cases poor yields and/or full reduction products were observed. However, inspired by the work of Nagashima,^14^ an attempted reduction of **3 a** using substoichiometric Vaska’s complex [IrCl(CO)(PPh_3_)_2_]^15^ (2.5 mol %) and silane (TMDS or PMDS)^16^ resulted in the desirable formation of **5 a** in 23 and 36 % yield, respectively (Table 1, entries 2 and 3). Quenching the reaction with 1 M HCl allowed efficient removal of excess silane and its by-products. Basification, extraction and purification afforded **5 a** in improved yield (Table 1, entry 4). With this promising method in hand we attempted to lower the catalyst loading (Table 1, entries 4–6). Pleasingly lowering to 0.1 % had little detrimental impact on the yield; however, for practicality (in weighing out the catalyst) we chose to use 0.5 mol % of Vaska’s complex. A solvent screen (Table 1, entries 7–9) revealed that toluene was indeed the best solvent. The concentration of the reaction was an important parameter, with high dilution leading to an increased yield of 80 % (Table 1, entries 10 and 11). After optimization we demonstrated the reaction was scalable; using 2 g of **3 a** bicycle **5 a** was formed in good yield and excellent diastereoselectivity (Table 1, entry 12).^17^

**Table 1:** Optimization results.

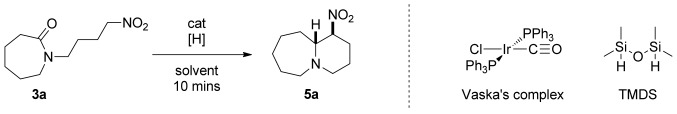										
Entry^[a]^	*T*	Solvent	Conc. [M]	Catalyst	Cat. loading [mol %]	Silane	Silane [equiv]	Work up	Yield^[b]^ [%]	d.r.^[c]^
1^[d]^	RT	toluene	0.05	[Ti(O*i*Pr_4_)]	210	TMDS	2.1	silica gel^[e]^	17	98:2
2	RT	toluene	0.05	[IrCl(CO)(PPh_3_)_2_]	2.5	TMDS	2.0	silica gel^[e]^	23	97:3
3	RT	toluene	0.05	[IrCl(CO)(PPh_3_)_2_]	2.5	PMDS	2.0	silica gel^[e]^	36	88:12
4	RT	toluene	0.05	[IrCl(CO)(PPh_3_)_2_]	2.5	TMDS	2.0	HCl^[f]^	53	88:12
5	RT	toluene	0.05	[IrCl(CO)(PPh_3_)_2_]	0.5	TMDS	2.0	HCl^[f]^	68	89:11
6	RT	toluene	0.05	[IrCl(CO)(PPh_3_)_2_]	0.1	TMDS	2.0	HCl^[f]^	55	91:9
7	RT	hexane	0.05	[IrCl(CO)(PPh_3_)_2_]	0.5	TMDS	2.0	HCl^[f]^	53	91:9
8	RT	THF	0.05	[IrCl(CO)(PPh_3_)_2_]	0.5	TMDS	2.0	HCl^[f]^	50	86:14
9	RT	CH_2_Cl_2_	0.05	[IrCl(CO)(PPh_3_)_2_]	0.5	TMDS	2.0	HCl^[f]^	48	88:12
10	RT	toluene	0.25	[IrCl(CO)(PPh_3_)_2_]	0.5	TMDS	2.0	HCl^[f]^	54	91:9
11	RT	toluene	0.01	[IrCl(CO)(PPh_3_)_2_]	0.5	TMDS	2.0	HCl^[f]^	80	88:12
12^[g]^	RT	toluene	0.01	[IrCl(CO)(PPh_3_)_2_]	0.5	TMDS	2.0	HCl^[f]^	71	98:2

[a] All reactions carried out on 0.10 g of **3 a** unless otherwise started, the major by-product was the corresponding fully reduced lactam (1-(4-nitrobutyl)azepane). [b] Isolated yield after purification. [c] d.r. measured by NMR spectroscopy of the isolated products. [d] Reaction time 28 h. [e] Reaction mixture was concentrated in vacuo and injected directly onto a silica gel column for chromatography. [f] Reaction was extracted with HCl (1 M), basified (K_2_CO_3_) and extracted into ether. [g] Reaction performed on 2 g of **3 a**.

NMR experiments were performed to identify the intermediates present at each stage of the reaction. Pure starting material is shown in Figure 1 spectrum A and starting material with TMDS and mesitylene internal standard is shown in spectrum B. On addition of Vaska’s complex, rapid (5 mins) and clean conversion to the enamine intermediate **7** was witnessed (Figure 1, spectrum **C**). On addition of HCl, iminium ion **8** was clearly observed (Figure 1, spectrum **D**); subsequently, and in a separate reaction, iminium ion **8** was isolated and fully characterized.^18^ Upon basification with solid K_2_CO_3_ and extraction the desired bicycle **5 a** was formed (Figure 1, spectrum **E**).

**Figure 1:**
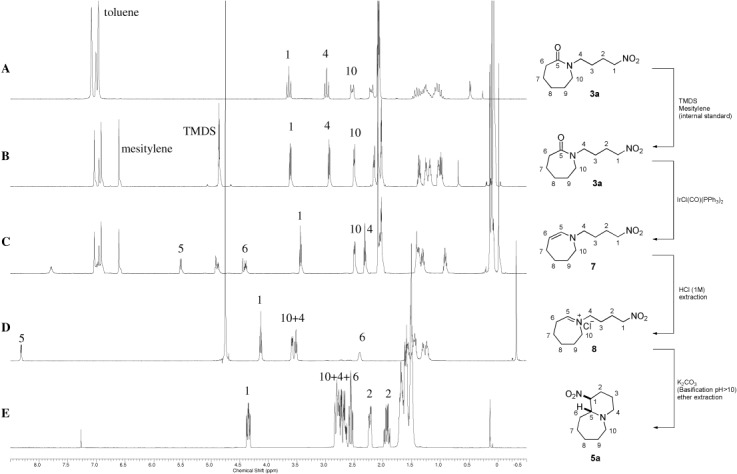
^1^H NMR spectra of starting material, intermediates and the desired product at selected stages of the reaction. Spectra A, B, and C were measured in [D_8_]toluene. Spectrum D was obtained in D_2_O and spectrum E was measured in CDCl_3_.

Given the direct evidence for the presence of each intermediate at their respective stages in the reaction we can postulate that the reaction proceeds by the mechanism shown in Scheme 3. The iridium complex catalyses the partial reduction of amide **3 a** to the siloxy intermediate **9**,^19^ elimination and subsequent loss of a proton reveals enamine **7**. Only upon acidic workup does the enamine reprotonate to give the stable, water-soluble, iminium ion **8**. Upon addition of solid potassium carbonate, the resulting rise in pH to >10 facilitates smooth cyclization of the putative nitronate **10** to form product bicycle **5 a**.

**Scheme 3:**
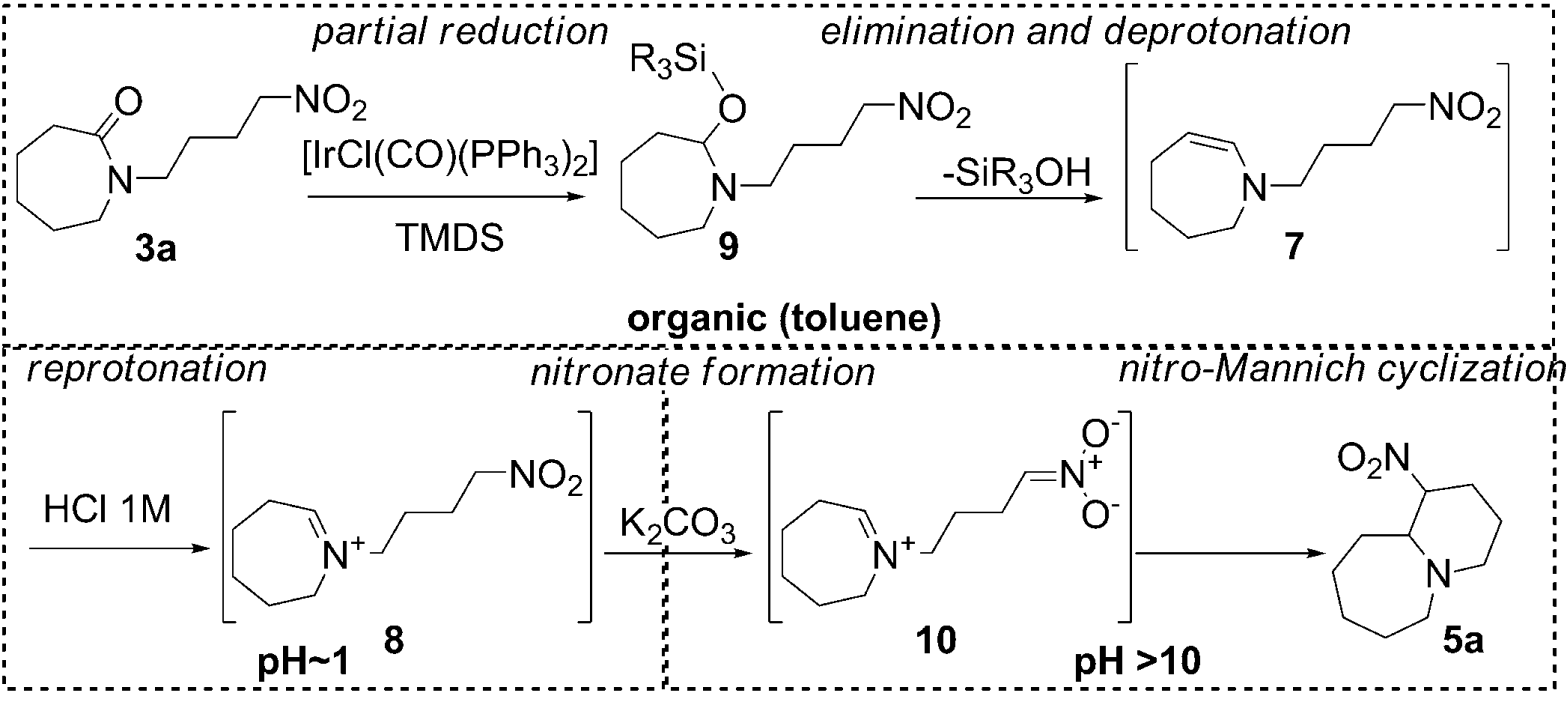
Postulated reaction mechanism.

With optimized conditions in hand we proceeded to assess the scope of the reaction (Figure 2). The size of the lactam ring could be varied from five-membered up to eight-membered with all substrates progressing in good to excellent d.r. (**5 a**–**5 d**). The reaction also tolerated ether functionality as demonstrated in the synthesis of oxazapane **5 e**. The method could be extended by lengthening the nitroalkane tether to access seven-membered rings on ring closure as exemplified by **5 f** and **5 g**. Shortening the nitroalkane tether was also possible, with bicycles **5 h** and **5 i** being formed in good yield albeit with a reduction in diastereoselectivity.

**Figure 2:**
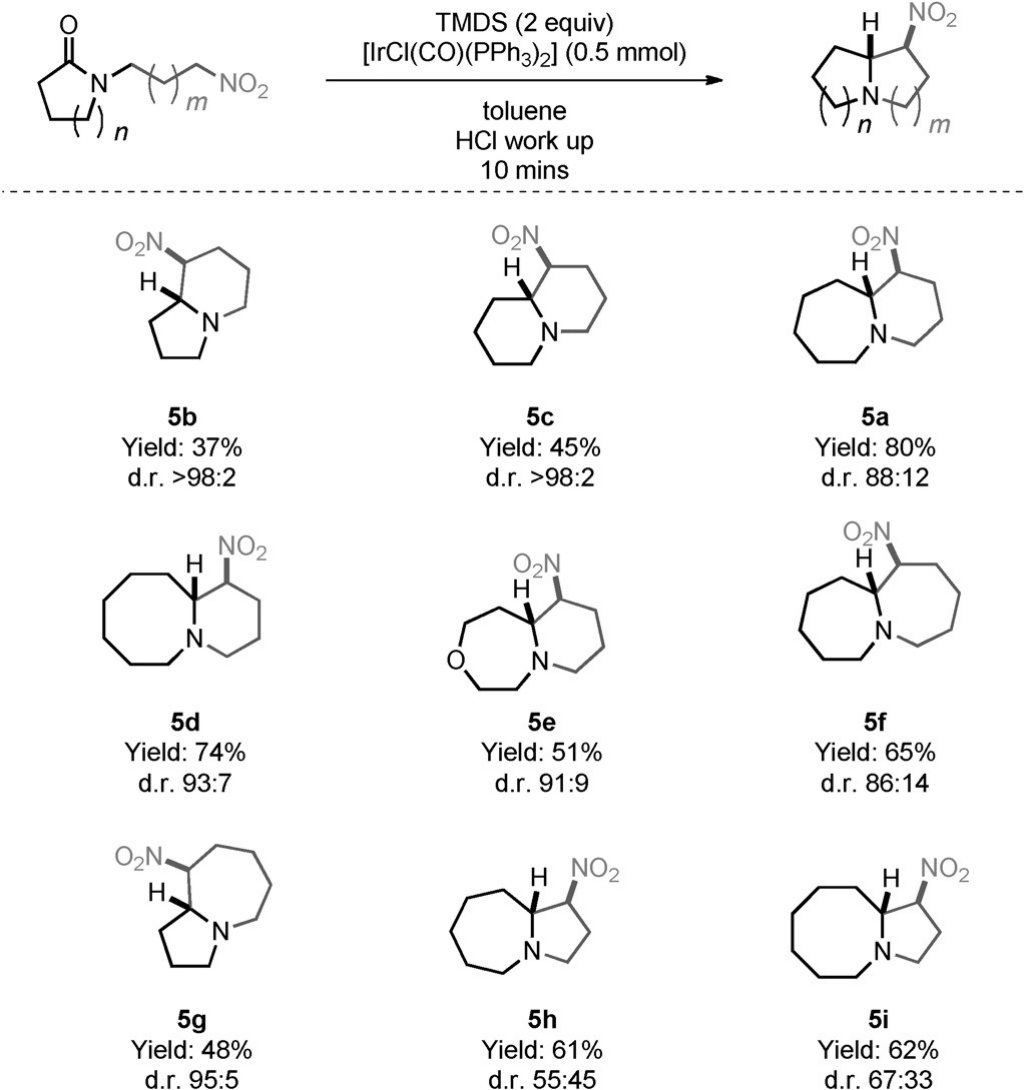
Reaction scope (aliphatic tether).

Substrates possessing an arene group in the nitroalkane tether also cyclized to their respective tricyclic products (**5 j**–**5 l**) in good to excellent yields (52–81 %) and diastereoselectivities up to 98:2 (Figure 3).

**Figure 3:**
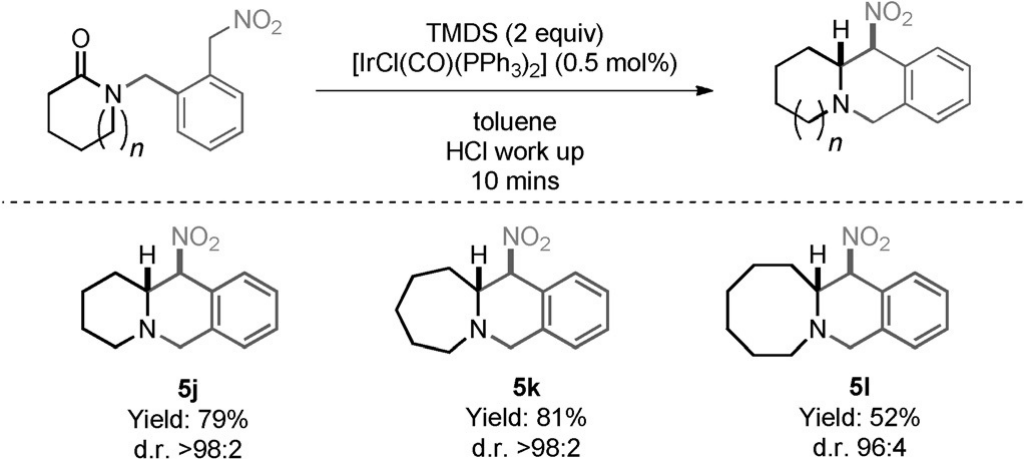
Reaction scope (aryl-linked tether).

Application of the methodology to the synthesis of (±)-*epi*-epiquinamide^20^ was demonstrated from bicycle **5 c**. Following the key reductive nitro-Mannich cyclization step, Raney nickel reduction and concommitant acetylation of bicycle **5 c** allowed the synthesis of natural product (±)-*epi*-epiquinamide^20^ in four steps from valerolactam and dibromobutane starting materials (Scheme 4).^18^ The synthesis of (±)-*epi*-epiquinamide confirmed the relative stereochemistry of **5 c** as (R*,S*) by comparison with literature data.^20^ Compounds **5 f** and **5 g** (formed by a seven-membered ring cyclization) were also assigned as (R*,S*) by NOE spectroscopy data from compound **5 f**. All other compounds were assigned as (R*,S*) by analogy to **5 c** and **5 f**.

**Scheme 4:**
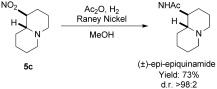
Total synthesis of (±)-*epi*-epiquinamide.

In conclusion, we have developed a novel chemoselective reductive cyclization. The process is unprecedented, fast, efficient and stereoselective and provides heterocyclic compounds with cores that are abundant in nature and make up classes of alkaloids that show potent biological activity. We have gained a thorough understanding of the reaction mechanism through NMR studies and have applied the new methodology to the total synthesis of (±)-*epi*-epiquinamide in four steps. We are currently developing an asymmetric variant of this reaction and expanding the concept of the reductive cyclization to include a range of new substrates, all of which will be disclosed in due course.

## Experimental Section

TMDS (2.0 equiv) and [IrCl(CO)(PPh_3_)_2_] (0.005 equiv) were added to a stirred solution of nitro-lactam **3** (1.0 equiv) in toluene (0.01 M) under an inert atmosphere at room temperature. The resulting solution was stirred for 10 mins until complete conversion of the starting material was observed (TLC), and then quenched with 1 M HCl (12 mL mmol^−1^ **3**). The aqueous layer was separated and the organic layer was extracted (1 M HCl, 3×12 mL mmol^−1^ **3**). The combined aqueous extracts were washed (Et_2_O, 3×6 mL mmol^−1^ **3**) and basified to pH>10 (K_2_CO_3_). The aqueous layer was then extracted (Et_2_O, 3×6 mL mmol^−1^ **3**), and the organic phases combined, dried (MgSO_4_), filtered and concentrated in vacuo. The residue was purified by FCC to yield the desired compound **5**.

## Supporting Information

As a service to our authors and readers, this journal provides supporting information supplied by the authors. Such materials are peer reviewed and may be re-organized for online delivery, but are not copy-edited or typeset. Technical support issues arising from supporting information (other than missing files) should be addressed to the authors.

miscellaneous_information
