# Supplementary material for: Iridium-Catalyzed Reductive Nitro-Mannich Cyclization
Source: Chemistry. 2014 Nov 14;21(1):111–4. doi: 10.1002/chem.201405256 (PMC4730865; doi:10.1002/chem.201405256)
Supplement: Supplementary file 1 — miscellaneous_information [file chem0021-0111-sd1.pdf]

CHEMISTRY
A European Journal

Supporting Information

© Copyright Wiley-VCH Verlag GmbH & Co. KGaA, 69451 Weinheim, 2014

Iridium-Catalyzed Reductive Nitro-Mannich Cyclization

Alex W. Gregory, Alan Chambers, Alison Hawkins, Pavol Jakubec, and Darren J. Dixon*[a]

chem_201405256_sm_miscellaneous_information.pdf

1. Contents

2. Experimental Section 4

2.1 General Experimental 4

2.1.1 General Experimental Techniques 4

2.1.2 Solvents and Reagents 4

2.1.3 Chromatography 4

2.1.4 Spectroscopy 5

2.1.5 Melting Points 5

2.1.6 Names 5

3. Experimental 6

3.1.1 General procedure A for the synthesis of *N*-alkylated lactams 11 6

3.1.2 Preparation and characterisation of 1-(4-bromobutyl)azepan-2-one (11a) 6

3.1.3 Preparation and characterisation of 1-(4-bromobutyl)pyrrolidin-2-one (11b) .7

3.1.4 Preparation and characterisation of 1-(5-bromopentyl)pyrrolidin-2-one (11g) 8

3.1.5 Preparation and characterisation of 1-(4-bromobutyl)piperidin-2-one (11c) ... 8

3.1.6 Preparation and characterisation of 1-(5-bromopentyl)azepan-2-one (11f) 9

3.1.7 Preparation and characterisation of 1-(4-bromobutyl)azocan-2-one (11d) 10

3.1.8 Preparation and characterisation of 4-(4-bromobutyl)-1,4-oxazepan-5-one (11e) 10

3.1.9 Preparation and characterisation of 1-[2-(bromomethyl)benzyl]piperidin-2-one (11j) 11

3.1.10 Preparation and characterisation of 1-[2-(bromomethyl)benzyl]azepan-2-one (11k) 12

3.1.11 Preparation and characterisation of 1-[2-(bromomethyl)benzyl]azocan-2-one (11l) 12

3.1.12 Preparation and characterisation of 1-(3-chloropropyl)azepan-2-one (11h) 13

3.1.13 Preparation and characterisation of 1-(3-chloropropyl)azocan-2-one (11i) 14

3.2 General procedure B for the synthesis of nitrated *N*-alkylated lactams 3 ... 14

3.2.1 Preparation and characterisation of 1-(4-Nitrobutyl)pyrrolidin-2-one (3b) 15

3.2.2 Preparation and characterisation of 1-(4-Nitrobutyl)azepan-2-one (3a) 15

3.2.3 Preparation and characterisation of 1-(5-Nitropentyl)pyrrolidin-2-one (3g) . 16

3.2.4 Preparation and characterisation of 1-(4-Nitrobutyl)piperidin-2-one (3c) 16

3.2.5 Preparation and characterisation of 1-(5-Nitropentyl)azepan-2-one (3f) 17

3.2.6 Preparation and characterisation of 1-(4-Nitrobutyl)azocan-2-one (3d) 18

3.3 General procedure C for the synthesis of nitrated N-alkylated lactams 3e and 5k 18

3.3.1 Preparation and characterisation of 4-(4-nitrobutyl)-1,4-oxazepan-5-one (3e) 19

3.3.2 Preparation and characterisation of 1-[2-(nitromethyl)benzyl]azepan-2-one (3k) 19

3.4 General procedure D for the synthesis of nitrated *N*-alkylated lactams 3h and 3i 20

3.4.1 Preparation and characterisation of 1-(3-nitropropyl)azepan-2-one (3h) 20

3.4.2 Preparation and characterisation of 1-(3-nitropropyl)azocan-2-one (3i) 21

3.5 General procedure E for the synthesis of nitrated N-alkylated lactams 3j and 3l 22

3.5.1 Preparation and characterisation of 1-[2-(nitromethyl)benzyl]piperidin-2-one (3j) 22
3.5.2 Preparation and characterisation of 1-[2-(nitromethyl)benzyl]azocan-2-one (3l) 23
3.6 General procedure F for the synthesis of nitrated bicycles 5. 24
3.6.1 Preparation and characterisation of (R*,S*)-8-nitrooctahydroindolizine (5b) 24
3.6.2 Preparation and characterisation of (R*,S*)-1-nitrooctahydro-2H-quinolizine (5c) 25
3.6.3 Preparation and characterisation of (R*,S*)-1-nitrodecahydropyrido[1,2-*a*]azepine (5a) 25
3.6.4 Preparation and characterisation of (R*,S*)-1-nitrodecahydro-2*H*-pyrido[1,2-*a*]azocine (5d) 26
3.6.5 Preparation and characterisation of (R*,S*)-10-nitrooctahydro-2*H*-pyrido[1,2-*d*][1,4]oxazepine (5e) 27
3.6.6 Preparation and characterisation of (R*,S*)-1-nitrodecahydro-1*H*-azepino[1,2-*a*]azepine (5f) 28
3.6.7 Preparation and characterisation of (R*,S*)-9-nitrooctahydro-1*H*-pyrrolo[1,2-*a*]azepine (5g) 29
3.6.8 Preparation and characterisation of (R*,S*)-1-nitrooctahydro-1*H*-pyrrolo[1,2-*a*]azepine (5h) 29
3.6.9 Preparation and characterisation of (R*,S*)-1-nitrodecahydropyrrolo[1,2-*a*]azocine (5i) 30
3.6.10 Preparation and characterisation of (R*,*S**)-11-nitro-1,3,4,6,11,11a-hexahydro-2*H*-pyrido[1,2-*b*]isoquinoline (5j) 31
3.6.11 Preparation and characterisation of (R*,*S**)-12-nitro-5,7,8,9,10,11,11a,12-octahydroazepino[1,2-*b*]isoquinoline (5k) 32
3.6.12 Preparation and characterisation of (R*,*S**)-13-nitro-7,8,9,10,11,12,12a,13-octahydro-5*H*-azocino[1,2-*b*]isoquinoline (5l) 33
3.7 Preparation and characterisation of 1-(4-nitrobutyl)-2,3,4,5-tetrahydro-1*H*-azepine (7) 33
3.8 Preparation and characterisation of 1-(4-nitrobutyl)-3,4,5,6-tetrahydro-2*H*-azepinium chloride (8) 34
3.9 Preparation and characterisation of *N*-(octahydro-2*H*-quinolizin-1-yl)acetamide ((±)-*epi*-epiquinamide) 35
4. ^{1}HNMR and ^{13}CNMR Spectra 36
4.1 Nitroalkane Spectra 36
4.1.1 ^{1}HNMR spectrum of 1-(4-Nitrobutyl)pyrrolidin-2-one (3b) 36
4.1.2 ^{13}CNMR spectrum of 1-(4-Nitrobutyl)pyrrolidin-2-one (3b) 37
4.1.3 ^{1}HNMR spectrum of 1-(4-Nitrobutyl)piperidin-2-one (3c) 38
4.1.4 ^{13}CNMR spectrum of 1-(4-Nitrobutyl)piperidin-2-one (3c) 39
4.1.5 ^{1}HNMR spectrum of 1-(4-Nitrobutyl)azepan-2-one (3a) 40
4.1.6 ^{13}CNMR spectrum of 1-(4-Nitrobutyl)azepan-2-one (3a) 41
4.1.7 ^{1}HNMR spectrum of 1-(4-Nitrobutyl)azocan-2-one (3d) 42
4.1.8 ^{13}CNMR spectrum of 1-(4-Nitrobutyl)azocan-2-one (3d) 43
4.1.9 ^{1}HNMR spectrum of 4-(4-nitrobutyl)-1,4-oxazepan-5-one (3e) 44
4.1.10 ^{13}CNMR spectrum of 4-(4-nitrobutyl)-1,4-oxazepan-5-one (3e) 45
4.1.11 ^{1}HNMR spectrum of 1-(5-Nitropentyl)azepan-2-one (3f) 46
4.1.12 ^{13}CNMR spectrum of 1-(5-Nitropentyl)azepan-2-one (3f) 47
4.1.13 ^{1}HNMR spectrum of 1-(5-Nitropentyl)pyrrolidin-2-one (3g) 48
4.1.14 ^{13}CNMR spectrum of 1-(5-Nitropentyl)pyrrolidin-2-one (3g) 49
4.1.15 ^{1}HNMR spectrum of 1-(3-nitropropyl)azepan-2-one (3h) 50
4.1.16 ^{13}CNMR spectrum of 1-(3-nitropropyl)azepan-2-one (3h) 51

4.1.17 ^{1}HNMR spectrum of 1-(3-nitropropyl)azocan-2-one (3i) 52
4.1.18 ^{13}CNMR spectrum of 1-(3-nitropropyl)azocan-2-one (3i) 53
4.1.19 ^{1}HNMR spectrum of 1-[2-(nitromethyl)benzyl]piperidin-2-one (3j)....54
4.1.20 ^{13}CNMR spectrum of 1-[2-(nitromethyl)benzyl]piperidin-2-one (3j)....55
4.1.21 ^{1}HNMR spectrum of 1-[2-(nitromethyl)benzyl]azepan-2-one (3k)....56
4.1.22 ^{1}HNMR spectrum of 1-[2-(nitromethyl)benzyl]azepan-2-one (3k)....57
4.1.23 ^{1}HNMR spectrum of 1-[2-(nitromethyl)benzyl]azocan-2-one (3l)....58
4.1.24 ^{13}CNMR spectrum of 1-[2-(nitromethyl)benzyl]azocan-2-one (3l) 59
4.2 Cyclized Nitro-Mannich Product Spectra 60
4.2.1 ^{1}HNMR spectrum of 8-Nitrooctahydroindolizine (5b)....60
4.2.2 ^{13}CNMR spectrum of 8-Nitrooctahydroindolizine (5b) 61
4.2.3 ^{1}HNMR spectrum of 1-Nitrooctahydro-2H-quinolizine (5b)....62
4.2.4 ^{13}CNMR spectrum of 1-Nitrooctahydro-2H-quinolizine (5c) 63
4.2.5 ^{1}HNMR spectrum of 1-Nitrodecahydropyrido[1,2-*a*]azepine (5a) 64
4.2.6 ^{13}CNMR spectrum of 1-Nitrodecahydropyrido[1,2-*a*]azepine (5a)....65
4.2.7 ^{1}HNMR spectrum of 1-Nitrodecahydro-2*H*-pyrido[1,2-*a*]azocine (5d)....66
4.2.8 ^{13}CNMR spectrum of 1-Nitrodecahydro-2*H*-pyrido[1,2-*a*]azocine (5d)....67
4.2.9 ^{1}HNMR spectrum of 10-nitrooctahydro-2*H*-pyrido[1,2-*d*][1,4]oxazepine (5e)68
4.2.10 ^{13}CNMR spectrum of 10-nitrooctahydro-2*H*-pyrido[1,2-*d*][1,4]oxazepine (5e) 69
4.2.11 ^{1}HNMR spectrum of 1-Nitrodecahydro-1*H*-azepino[1,2-*a*]azepine (5f)....70
4.2.12 ^{13}CNMR spectrum of 1-Nitrodecahydro-1*H*-azepino[1,2-*a*]azepine (5f)....71
4.2.13 NOE spectrum of 1-Nitrodecahydro-1*H*-azepino[1,2-*a*]azepine (5f)....72
4.2.14 NOESY spectrum of 1-Nitrodecahydro-1*H*-azepino[1,2-*a*]azepine (5f)....73
4.2.15 ^{1}HNMR spectrum of 9-Nitrooctahydro-1H-pyrrolo[1,2-*a*]azepine (5g) 74
4.2.16 ^{13}CNMR spectrum of 9-Nitrooctahydro-1*H*-pyrrolo[1,2-*a*]azepine (5g) 75
4.2.17 ^{1}HNMR spectrum of 1-nitrooctahydro-1*H*-pyrrolo[1,2-*a*]azepine (5h)....76
4.2.18 ^{13}CNMR spectrum of 1-nitrooctahydro-1*H*-pyrrolo[1,2-*a*]azepine (5h)....77
4.2.19 ^{1}HNMR spectrum of 1-nitrooctahydro-1*H*-pyrrolo[1,2-*a*]azepine mixed diastereomers (5h)....78
4.2.20 ^{13}CNMR spectrum of 1-nitrooctahydro-1*H*-pyrrolo[1,2-*a*]azepine mixed diastereomers (5h)....79
4.2.21 ^{1}HNMR spectrum of 1-nitrodecahydropyrrolo[1,2-*a*]azocine (5i)....80
4.2.22 ^{13}CNMR spectrum of 1-nitrodecahydropyrrolo[1,2-*a*]azocine (5i)....81
4.2.23 ^{1}HNMR spectrum of 1-nitrodecahydropyrrolo[1,2-*a*]azocine mixed diastereomer (5i) 82
4.2.24 ^{13}CNMR spectrum of 1-nitrodecahydropyrrolo[1,2-*a*]azocine mixed diastereomer (5i) 83
4.2.25 ^{1}HNMR spectrum of 11-nitro-1,3,4,6,11,11a-hexahydro-2*H*-pyrido[1,2-*b*]isoquinoline (5j) 84
4.2.26 ^{1}HNMR spectrum of 11-nitro-1,3,4,6,11,11a-hexahydro-2*H*-pyrido[1,2-*b*]isoquinoline (5j) 85
4.2.27 ^{1}HNMR spectrum of 12-nitro-5,7,8,9,10,11,11a,12-octahydroazepino[1,2-*b*]isoquinoline (5k)....86
4.2.28 ^{13}CNMR spectrum of 12-nitro-5,7,8,9,10,11,11a,12-octahydroazepino[1,2-*b*]isoquinoline (5k)....87
4.2.29 ^{1}HNMR spectrum of 13-nitro-7,8,9,10,11,12,12a,13-octahydro-5*H*-azocino[1,2-*b*]isoquinoline (5l)....88
4.2.30 ^{13}CNMR spectrum of 13-nitro-7,8,9,10,11,12,12a,13-octahydro-5*H*-azocino[1,2-*b*]isoquinoline (5l)....89
4.2.31 ^{1}HNMR spectrum of 1-(4-Nitrobutyl)-2,3,4,5-tetrahydro-1*H*-azepine (7)....90
4.2.32 ^{13}CNMR spectrum of 1-(4-Nitrobutyl)-2,3,4,5-tetrahydro-1*H*-azepine (7) ..91

4.2.33 ^{1}HNMR spectrum of 1-(4-Nitrobutyl)-3,4,5,6-tetrahydro-2*H*-azepinium chloride (8)......92
4.2.34 ^{13}CNMR spectrum of 1-(4-Nitrobutyl)-3,4,5,6-tetrahydro-2*H*-azepinium chloride (8)......93
4.2.35 ^{1}HNMR spectrum of (±)-*epi*-epiquinamide......94
4.2.36 ^{13}CNMR spectrum of (±)-*epi*-epiquinamide......95

2. Experimental Section

2.1 General Experimental

2.1.1 General Experimental Techniques

For reactions requiring anhydrous conditions, glassware was dried in an oven at 100 °C and reactions were carried out under a nitrogen/argon atmosphere. Room temperature (RT) refers to 20-25 °C. Temperatures of 0 °C and -78 °C were achieved using an ice-bath and dry ice in acetone respectively.

2.1.2 Solvents and Reagents

Bulk solutions were concentrated under reduced pressure using a Büchi rotary evaporator. All solvents were commercially supplied or provided by the communal stills of the Chemistry Research Laboratory, Oxford. These stills operate using activated alumina columns. Petroleum ether (PE) refers to distilled light petroleum with boiling points in the range of 30 - 40 °C. Commercially available reagents were used as received.

2.1.3 Chromatography

All reactions were monitored by thin-layer chromatography (TLC) where appropriate using Merck Kiesel gel 60 F_{254} (230-400 mesh) silica plates which were visualised by UV-light (250 nm) or by staining using aqueous potassium permanganate solutions where appropriate. Column chromatography was carried out using Merck Kieselgel 60 silica gel (230-400 mesh).

2.1.4 Spectroscopy

All ^{1}H and ^{13}C nuclear magnetic resonance (NMR) spectra were collected on either a Bruker DPX400 (400 MHz ^{1}H, 100 MHz ^{13}C), Bruker DQX400 (400 MHz ^{1}H, 100 MHz ^{13}C), Bruker AVC500 (500 MHz ^{1}H, 125 MHz ^{13}C), Bruker AVN400 (400 MHz ^{1}H, 100 MHz ^{13}C) or Bruker DPX300 (300 MHz ^{1}H, 75 MHz ^{13}C) and in the deuterated solvent stated. Chemical shift values (δ) are reported relative to tetramethylsilane (δ = 0 ppm) using the residual solvent peak as an internal reference. Assignments were aided by COSY, DEPT, HSQC, NOESY and TOCSY experiments and were only quoted where the assignments are clearly assignable. The abbreviations s, d, t, q, quin, sept, br, m and e.g. "t" denote singlet, doublet, triplet, quartet, quintet, septet, broad, multiplet and apparent multiplicity respectively. All coupling constants, *J*, are quoted in Hz. Low resolution mass spectrometric (*m/z*) data was acquired by electrospray ionisation (ESI) on an LCT Premier Open Access instrument. High resolution mass spectra were recorded on a Bruker MicroTof mass spectrometer (ESI) by the internal service at the Department of Organic Chemistry, University of Oxford. Infrared spectra (ν_{max}) were recorded on a Bruker Tensor 27 FT-IR spectrometer as a thin film on a diamond ATR module. Only selected maximum absorbances are reported.

2.1.5 Melting Points

Melting points (m.p.) were recorded in degrees Celsius (°C), using a Leica Galen III hot-stage microscope apparatus and are reported uncorrected.

2.1.6 Names

Compound names are those generated by ACD LABS 12.0 software and ACD ilab online chemical database service following the IUPAC nomenclature.

3. Experimental

3.1.1 General procedure A for the synthesis of *N*-alkylated lactams 11

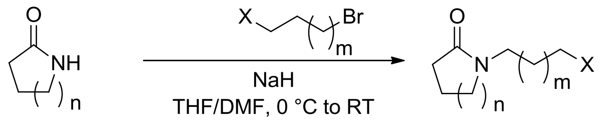

According to literature procedure,[1] to a suspension of NaH (60% dispersion in mineral oil, 1.5 eq.) in a mixture of THF:DMF (5:1, 0.09 M) was added the corresponding lactam (1.0 eq.) at 0 °C and stirred for 1 h. the corresponding bromoalkane (2.5 - 5.0 eq.) was then added to the mixture at 0 °C. The reaction mixture was warmed to room temperature and stirred 18 h. Upon completion the reaction was quenched with water. The organic phase was separated and the aqueous layer was extracted with EtOAc. The combined organic extracts were washed with brine, dried over $MgSO_4$, filtered and concentrated *in vacuo*. The residue was purified by FCC to yield the title compound **11**.

3.1.2 Preparation and characterisation of 1-(4-bromobutyl)azepan-2-one (11a)

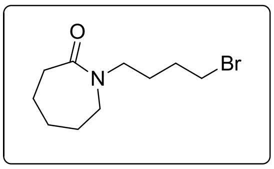

Prepared according to general procedure **A** using ε-caprolactam (5.00 g, 44.0 mmol) and 1,4-dibromobutane (220 mmol). Purification by FCC yielded *N*-alkyl lactam **11a** (8.20 g, 75%) as a yellow oil. **IR** ν_{max}(film)/cm^{-1} 1634 (C=O); **^{1}H NMR** (400 MHz, $CDCl_3$) δ_H 1.53 - 1.71 (m, 8H, C$\underline{H_2}$$CH_2CH_2$Br, C(O)$CH_2C\underline{H_2}$, C(O)$CH_2CH_2C\underline{H_2}$, C(O)$CH_2CH_2CH_2C\underline{H_2}$), 1.79 ("quin", *J* = 7.0, 2H, C$\underline{H_2}$$CH_2$Br), 2.40 - 2.50 (m, 2H, C(O)C$\underline{H_2}$), 3.24 - 3.30 (m, 2H, C(O)$CH_2CH_2CH_2CH_2$C$\underline{H_2}$), 3.33 (t, *J* = 7.0, 2H, C$\underline{H_2}$$CH_2CH_2CH_2$Br), 3.38 (t, *J* = 6.5, 2H, C$\underline{H_2}$Br); **^{13}C NMR** (100 MHz, $CDCl_3$) δ_C 23.4,

26.5, 28.6, 29.8, 29.9 ($\underline{C}H_2CH_2Br$), 33.7 ($\underline{C}H_2Br$), 37.2 ($C(O)\underline{C}H_2$), 46.9 ($\underline{C}H_2CH_2CH_2CH_2Br$), 49.4 ($C(O)CH_2CH_2CH_2CH_2\underline{C}H_2$), 175.7 ($\underline{C}O$); ***m/z*** (ESI$^+$) 270 ([M+Na]$^+$), **HRMS** (ES$^+$) exact mass calculated for [M+Na]$^+$ ($C_{10}H_{18}BrNNaO^+$) requires *m/z* 270.0464 and 272.0444, found *m/z* 270.0469 and 272.0450.

3.1.3 Preparation and characterisation of 1-(4-bromobutyl)pyrrolidin-2-one (11b)

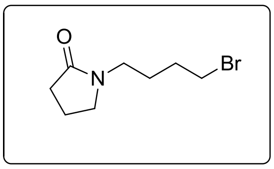

Prepared according to general procedure **A** using 2-pyrrolidinone (0.100 g, 1.18 mmol) and 1,4-dibromobutane (220 mmol). Purification by FCC yielded *N*-alkyl lactam **11b** (0.22 g, 86%) as a yellow oil. **IR** ν_{max}(film)/cm^{-1} 1673 (C=O); **^{1}H NMR** (400 MHz, $CDCl_3$) δ_H 1.63 - 1.72 (m, 2H, $C\underline{H_2}(CH_2)_2Br$), 1.84 (“quin”, J = 7.0, 2H, $C\underline{H_2}CH_2Br$), 1.94 - 2.06 (m, 2H, $C(O)CH_2C\underline{H_2}$), 2.37 (t, J = 8.0, 2H, $C(O)C\underline{H_2}$), 3.30 (t, J = 7.0, 2H, $C\underline{H_2}(CH_2)_3Br$), 3.37 (t, J = 7.0, 2H, $C(O)(CH_2)_2C\underline{H_2}$), 3.43 (t, J = 6.5, 2H, $C\underline{H_2}Br$); **^{13}C NMR** (100 MHz, $CDCl_3$) δ_C 17.8 ($C(O)CH_2\underline{C}H_2$), 25.6 ($\underline{C}H_2(CH_2)_2Br$), 29.7 ($\underline{C}H_2CH_2Br$), 30.9 ($C(O)\underline{C}H_2$), 33.3 ($\underline{C}H_2Br$), 41.3 ($\underline{C}H_2(CH_2)_3Br$), 46.9 ($C(O)(CH_2)_2\underline{C}H_2$), 175.0 ($\underline{C}O$); ***m/z*** (ESI$^+$) 220 ([M+H]$^+$); **HRMS** (ES$^+$) exact mass calculated for [M+Na]$^+$ ($C_8H_{14}BrNNaO^+$) requires *m/z* 242.0151 and 244.0131, found *m/z* 242.0150 and 244.0132. ^{1}H NMR data in agreement with that previously reported in the literature.[2]

3.1.4 Preparation and characterisation of 1-(5-bromopentyl)pyrrolidin-2-one (11g)

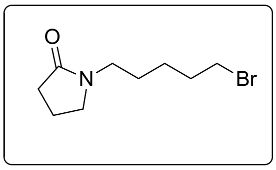

Prepared according to general procedure **A** using 2-pyrrolidinone (1.00 g, 11.8 mmol) and 1,5-dibromopentane (220 mmol). Purification by FCC yielded *N*-alkyl lactam **11g** (1.91 g, 69%) as a pale yellow oil. **IR** ν_{max}(film)/cm^{-1} 1676 (C=O); **^{1}H NMR** (400 MHz, $CDCl_3$) δ_H 1.34 - 1.45 (m, 2H, C$\underline{H_2}$(CH_2)$_2$Br), 1.45 - 1.57 (m, 2H, C$\underline{H_2}$(CH_2)$_3$Br), 1.79 - 1.90 (m, 2H, C$\underline{H_2}$$CH_2$Br), 1.93 - 2.05 (m, 2H, C(O)CH_2C$\underline{H_2}$), 2.34 (t, *J* = 8.0, 2H, C(O)C$\underline{H_2}$), 3.24 (t, *J* = 7.0, 2H, C$\underline{H_2}$(CH_2)$_4$Br), 3.29 - 3.42 (m, 4H, C$\underline{H_2}$Br, C(O)(CH_2)$_2$C$\underline{H_2}$); **^{13}C NMR** (100 MHz, $CDCl_3$) δ_C 17.9 (C(O)$CH_2$$\underline{C}H_2$), 25.2 ($\underline{C}H_2$($CH_2$)$_2$Br), 26.4 ($\underline{C}H_2$($CH_2$)$_3$Br), 31.0 (C(O)$\underline{C}H_2$), 32.2 ($\underline{C}H_2$$CH_2$Br), 33.6 ($\underline{C}H_2$Br), 42.1 ($\underline{C}H_2$($CH_2$)$_4$Br), 47.1 (C(O)($CH_2$)$_2$$\underline{C}H_2$), 174.9 ($\underline{C}$O); ***m/z*** (ESI$^+$) 234 ([M+H]$^+$; **HRMS** (ES$^+$) exact mass calculated for [M+Na]$^+$ ($C_9H_{16}BrNNaO^+$) requires *m/z* 256.0307 and 258.0287, found *m/z* 256.0307 and 258.0287.

3.1.5 Preparation and characterisation of 1-(4-bromobutyl)piperidin-2-one (11c)

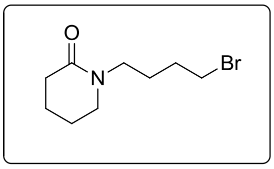

Prepared according to general procedure **A** using δ-valerolactam (5.00 g, 50.4 mmol) and 1,4-dibromobutane (220 mmol). Purification by FCC yielded *N*-alkyl lactam **11c** (7.07 g, 60%) as a yellow oil. **IR** ν_{max}(film)/cm^{-1} 1631 (C=O); **^{1}H NMR** (400 MHz, $CDCl_3$) δ_H 1.61 - 1.92 (m, 8H, C$\underline{H_2}$$CH_2Br, C\underline{H_2}$($CH_2$)$_2$Br, C(O)$CH_2C\underline{H_2}$, C(O)($CH_2$)$_2C\underline{H_2}$), 2.38 (t, *J*

= 6.0, 2H, $C(O)C\underline{H_2}$), 3.22 (t, *J* = 5.5, 2H, $C(O)(CH_2)_3C\underline{H_2}$), 3.30 - 3.43 (m, 4H, $C\underline{H_2}Br$, $C\underline{H_2}(CH_2)_3Br$); **^{13}C NMR** (100 MHz, $CDCl_3$) δ_C 21.2, 23.1, 25.4, 29.7, 32.1 ($C(O)\underline{C}H_2$), 33.4, 45.7 ($\underline{C}H_2Br$, $\underline{C}H_2(CH_2)_3Br$), 47.6 ($C(O)(CH_2)_3\underline{C}H_2$), 169.6 ($\underline{C}O$). ***m/z*** ($ESI^+$) 234 $[M+H]^+$; **HRMS** (ES^+) exact mass calculated for $[M+Na]^+$ ($C_9H_{16}BrNNaO^+$) requires *m/z* 256.0307 and 258.0287, found *m/z* 256.0306 and 258.0288. ^{1}H NMR data in agreement with that previously reported in the literature.[2]

3.1.6 Preparation and characterisation of 1-(5-bromopentyl)azepan-2-one (11f)

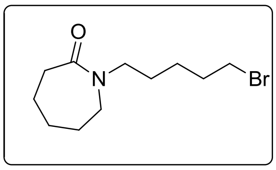

Prepared according to general procedure **A** using ε-caprolactam (1.00 g, 8.85 mmol) and 1,5-dibromopentane (220 mmol). Purification by FCC yielded *N*-alkyl lactam **11f** (1.69 g, 73%) as a pale yellow oil. **IR** ν_{max}(film)/cm^{-1} 1636 (C=O); **^{1}H NMR** (500 MHz, $CDCl_3$) δ_H 1.36 - 1.44 (m, 2H, $C\underline{H_2}(CH_2)_2Br$), 1.45 - 1.54 (m, 2H, $C\underline{H_2}(CH_2)_3Br$), 1.56 - 1.73 (m, 6H, $C(O)CH_2C\underline{H_2}$, $C(O)(CH_2)_2C\underline{H_2}$, $C(O)(CH_2)_3C\underline{H_2}$), 1.85 (“quin”, *J* = 7.0, 2H, $C\underline{H_2}CH_2Br$), 2.45 - 2.49 (m, 2H, $C(O)C\underline{H_2}$), 3.28 - 3.31 (m, 2H, $C(O)(CH_2)_4C\underline{H_2}$), 3.33 (t, *J* = 7.5, 2H, $C\underline{H_2}(CH_2)_4Br$), 3.37 (t, *J* = 7.0, 2H, $C\underline{H_2}Br$); **^{13}C NMR** (100 MHz, $CDCl_3$) δ_C 23.4, 25.3 ($\underline{C}H_2(CH_2)_2Br$), 27.2 ($\underline{C}H_2(CH_2)_3Br$), 28.6, 29.9, 32.4 ($\underline{C}H_2CH_2Br$), 33.7 ($\underline{C}H_2Br$), 37.2 ($C(O)\underline{C}H_2$), 47.8 ($\underline{C}H_2(CH_2)_4Br$), 49.5 ($C(O)(CH_2)_4\underline{C}H_2$), 175.5 ($\underline{C}O$); ***m/z*** ($ESI^+$) 262 ($[M+H]^+$); **HRMS** ($ES^+$) exact mass calculated for $[M+Na]^+$ ($C_{11}H_{20}BrNNaO^+$) requires *m/z* 284.0620 and 286.0600, found *m/z* 284.0621 and 286.0604.

3.1.7 Preparation and characterisation of 1-(4-bromobutyl)azocan-2-one (11d)

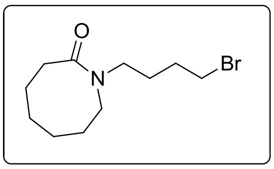

Prepared according to general procedure **A** using azocan-2-one (1.00 g, 7.86 mmol) and 1,4-dibromobutane (220 mmol). Purification by FCC yielded *N*-alkyl lactam **11d** (1.49 g, 72%) as a pale yellow oil. **IR** ν_{max}(film)/cm^{-1} 1627 (C=O); **^{1}H NMR** (400 MHz, $CDCl_3$) δ_H 1.35 - 1.51 (m, 4H, $C(O)(CH_2)_2C\underline{H_2}$, $C(O)(CH_2)_3C\underline{H_2}$), 1.53 - 1.84 (m, 8H, $C(O)(CH_2)_4C\underline{H_2}$, $C(O)CH_2C\underline{H_2}$, $C\underline{H_2}CH_2Br$, $C\underline{H_2}(CH_2)_2Br$), 2.37 - 2.44 (m, 2H, $C(O)C\underline{H_2}$), 3.26 (t, *J* = 7.5, 2H, $C\underline{H_2}(CH_2)_3Br$), 3.32 - 3.43 (m, 4H, $C(O)(CH_2)_5C\underline{H_2}$, $C\underline{H_2}Br$); **^{13}C NMR** (100 MHz, $CDCl_3$) δ_C 24.3 ($C(O)(CH_2)_3\underline{C}H_2$), 26.2, 26.3, 28.6 ($C(O)CH_2\underline{C}H_2$), 29.2 ($C(O)(CH_2)_4\underline{C}H_2$), 30.0 ($\underline{C}H_2CH_2Br$), 33.5 ($\underline{C}H_2Br$), 33.9 ($C(O)\underline{C}H_2$), 44.0 ($\underline{C}H_2(CH_2)_3Br$), 46.8 ($C(O)(CH_2)_5\underline{C}H_2$), 174.8 ($\underline{C}O$); ***m/z*** (ESI$^+$) 262 $[M+H]^+$; **HRMS** (ES$^+$) exact mass calculated for $[M+Na]^+$ ($C_{11}H_{20}BrNNaO^+$) requires *m/z* 284.0620 and 286.0600, found *m/z* 284.0614 and 286.0593.

3.1.8 Preparation and characterisation of 4-(4-bromobutyl)-1,4-oxazepan-5-one (11e)

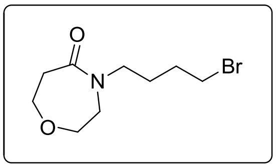

Prepared according to general procedure **A** using 1,4-oxazepan-5-one (0.900 g, 7.8 mmol) and 1,4-dibromobutane (220 mmol). Purification by FCC yielded *N*-alkyl lactam **11e** (1.68 g, 86%) as a clear colourless oil. **IR** ν_{max}(film)/cm^{-1} 1640 (C=O); **^{1}H NMR** (400 MHz, $CDCl_3$) δ_H 1.63 – 1.73 (m, 2H), 1.83 – 1.92 (m, 2H), 2.74 – 2.78 (m, 2H,

NC(O)C$\underline{H}_2$), 3.41 – 3.50 (m, 6H, (O)NC$\underline{H}_2$CH$_2$O, BrC$\underline{H}_2$, and Br(CH$_2$)$_3$C$\underline{H}_2$), 3.74 – 3.81 (m, 4H, (C(O)CH$_2$C$\underline{H}_2$OC$\underline{H}_2$); **^{13}C NMR** (100 MHz, CDCl$_3$) δ_C 26.4, 29.7, 33.5 ($\underline{C}$H$_2$Br), 41.2 (C(O)$\underline{C}$H$_2$), 47.4, 51.6, 65.5, 70.6, 174.3 ($\underline{C}$O); ***m/z*** (ESI$^+$) 250 ([M+H]$^+$, ^{79}Br); **HRMS** (ES$^+$) exact mass calculated for [M+Na]$^+$ ($C_9H_{16}BrNNaO_2^+$) requires *m/z* 272.0257 and 274.0236, found *m/z* 272.0260 and 274.0240.

3.1.9 Preparation and characterisation of 1-[2-(bromomethyl)benzyl]piperidin-2-one (11j)

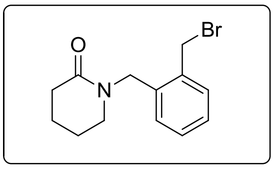

Prepared according to general procedure **A** using piperidin-2-one (3.00 g, 30 mmol) and 1,2-bis(bromomethyl)benzene (27.0 g, 105 mmol). Purification by FCC yielded *N*-alkyl lactam **11j** (2.79 g, 33%) as a colourless oil. **IR** ν_{max}(film)/cm^{-1} 1627 (C=O); **^{1}H NMR** (400 MHz, CDCl$_3$) δ_H 1.70 – 1.79 (m, 4H, C(O)CH$_2$C$\underline{H}_2$C$\underline{H}_2$,), 2.47 (t, *J* = 6.0, 2H, C(O)C$\underline{H}_2$), 3.10 (t, *J* = 6.0, 2H, NC$\underline{H}_2$), 4.52 (s, 2H, BrC$\underline{H}_2$), 4.70 (s, 2H, NC$\underline{H}_2$Ar), 7.10 – 7.12 (m, 1H, Ar$\underline{H}$), 7.19 – 7.24 (m, 2H, Ar$\underline{H}$), 7.30 – 7.32 (m, 1H, Ar$\underline{H}$); **^{13}C NMR** (100 MHz, CDCl$_3$) δ_C 21.2, 23.0, 31.5 (Br$\underline{C}$H$_2$), 32.3 (C(O)$\underline{C}$H$_2$), 47.2 (N$\underline{C}$H$_2$CH$_2$), 47.4 (N$\underline{C}$H$_2$Ar), 128.3 (Ar$\underline{C}$H), 129.0 (Ar$\underline{C}$H), 129.7 (Ar$\underline{C}$H), 131.3 (Ar$\underline{C}$H), 135.3 (Ar$\underline{C}_{quat}$), 136.7 (Ar$\underline{C}_{quat}$), 170.0 ($\underline{C}$O); ***m/z*** (ESI$^+$) 304 ([M+Na]$^+$); **HRMS** (ES$^+$) exact mass calculated for [M+Na]$^+$ ($C_{13}H_{16}BrNNaO^+$) requires *m/z* 304.0307 and 306.0287, found *m/z* 304.0308 and 306.0289.

3.1.10 Preparation and characterisation of 1-[2-(bromomethyl)benzyl]azepan-2-one (11k)

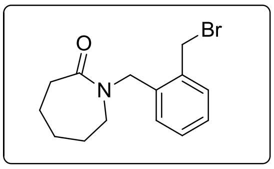

Prepared according to general procedure **A** using azepan-2-one (2.00 g, 17.6 mmol) and 1,2-bis(bromomethyl)benzene (11.5g, 44 mmol). Purification by FCC yielded *N*-alkyl lactam **11k** (2.62 g, 51%) as a colourless oil. **IR** ν_{max}(film)/cm^{-1} 1633 (C=O); **^{1}H NMR** (400 MHz, $CDCl_3$) δ_H 1.56 – 1.63 (m, 2H), 1.72 – 1.79 (m, 4H), 2.66 – 2.71 (m, 2H, CO$\underline{H_2}$), 3.29 – 3.34 (m, 2H, NC$\underline{H_2}$CH$_2$), 4.60 (s, 2H, ArC$\underline{H_2}$Br), 4.76 (s, 2H, NC$\underline{H_2}$Ar), 7.20 – 7.23 (m, 1H), 7.28 – 7.33 (m, 2H), 7.38 – 7.40 (m, 1H); **^{13}C NMR** (100 MHz, $CDCl_3$) δ_C 23.4, 27.8, 30.0, 31.5 (Ar$\underline{C}$H$_2$Br), 37.1 (CO$\underline{C}$H$_2$), 47.8 (N$\underline{C}$H$_2$Ar), 48.4 (N$\underline{C}$H$_2$CH$_2$), 128.1, 129.0, 129.6, 131.2, 136.0, 136.4, 176.1 ($\underline{C}$O); ***m/z*** (ESI$^+$) 296 ([M+H]$^+$, ^{79}Br), 297 ([M+H]$^+$, ^{81}Br); **HRMS** (ES$^+$) exact mass calculated for [M+Na]$^+$ ($C_{14}H_{18}BrNNaO^+$) requires *m/z* 318.0464 and 320.0444, found *m/z* 318.0462 and 320.0443.

3.1.11 Preparation and characterisation of 1-[2-(bromomethyl)benzyl]azocan-2-one (11l)

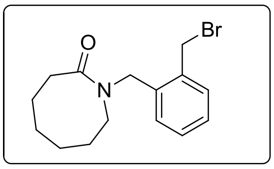

Prepared according to general procedure **A** using azocan-2-one (1.93 g, 15.2 mmol) and 1,2-bis(bromomethyl)benzene (9.9 g, 38 mmol). Purification by FCC yielded *N*-alkyl lactam **11l** (2.02 g, 43%) as a colourless oil. **IR** ν_{max}(film)/cm^{-1} 1627 (C=O); **^{1}H NMR** (400 MHz, $CDCl_3$) δ_H 1.50 – 1.62 (m, 4H), 1.65 - 1.71 (m, 2H), 1.86 – 1.92 (m, 2H), 2.65 – 2.70 (m, 2H, C(O)C$\underline{H_2}$), 3.38 – 3.43 (m, 2H, NC$\underline{H_2}$CH$_2$), 4.56 (s, 2H, BrC$\underline{H_2}$),

4.76 (s, 2H, $NC\underline{H_2}Ar$), 7.19 – 7.22 (m, 1H), 7.27 – 7.30 (m, 2H), 7.35 – 7.37 (m, 1H); **^{13}C NMR** (100 MHz, $CDCl_3$) δ_C 24.3, 26.3, 28.3, 28.9, 31.5 ($Br\underline{C}H_2$), 33.7 ($C(O)\underline{C}H_2$), 44.2 ($N\underline{C}H_2CH_2$), 45.4 ($N\underline{C}H_2Ar$), 128.2, 129.1, 129.4, 131.2, 135.5, 136.3, 175.8 ($\underline{C}O$); ***m/z*** (ESI^+) 306 ($[M+H]^+$, ^{79}Br), 308 ($[M+H]^+$, ^{81}Br), **HRMS** (ES^+) exact mass calculated for $[M+Na]^+$ ($C_{15}H_{20}BrNNaO^+$) requires *m/z* 332.0620 and 334.0601, found *m/z* 332.0624 and 334.0605.

3.1.12 Preparation and characterisation of 1-(3-chloropropyl)azepan-2-one (11h)

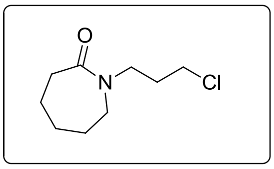

Prepared according to general procedure **A** using azepan-2-one (2.3 g, 20 mmol) and 1-bromo-3-chloropropane (4.9 ml, 50 mmol). Purification by FCC yielded *N*-alkyl lactam **11h** (2.59 g, 69%) as a colourless oil. **IR** ν_{max}(film)/cm^{-1} 1625(C=O); **^{1}H NMR** (400 MHz, $CDCl_3$) δ_H 1.59 – 1.78 (m, 6H, $C(O)CH_2C\underline{H_2}C\underline{H_2}C\underline{H_2}$), 2.01 (“quin”, *J* = 7.0, 2H, $ClCH_2C\underline{H_2}$), 2.50 – 2.55 (m, 2H, $C(O)C\underline{H_2}$), 3.36 – 3.41 (m, 2H, $NC\underline{H_2}CH_2CH_2CH_2$), 3.50 (t , *J* = 7.0, 2H, $ClC\underline{H_2}$), 3.56 (t, 2H, *J* = 7.0, $ClCH_2CH_2C\underline{H_2}$), **^{13}C NMR** (100 MHz, $CDCl_3$) δ_C 23.4, 28.7, 30.0, 31.2 ($ClCH_2C\underline{H_2}$), 37.3 ($C(O)\underline{C}H_2$), 42.6 ($Cl\underline{C}H_2$), 46.4 ($ClCH_2CH_2\underline{C}H_2$), 50.3 ($N\underline{C}H_2CH_2CH_2CH_2$), 176.1 ($\underline{C}(O)$); ***m/z*** ($ESI^+$) 190 ($[M+H]^+$); **HRMS** ($ES^+$) exact mass calculated for $[M+H]^+$ ($C_9H_{17}ClNO^+$) requires *m/z* 190.09932 and 19.09637, found *m/z* 190.09921 and 192.09620.

3.1.13 Preparation and characterisation of 1-(3-chloropropyl)azocan-2-one (11i)

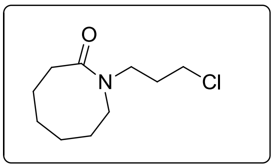

Prepared according to general procedure **A** using azocan-2-one (0.88 g, 6.9 mmol) and 1-bromo-3-chloropropane (1.7 ml, 17 mmol). Purification by FCC yielded *N*-alkyl lactam **11i** (0.98 g, 70%) as a colourless oil. **IR** ν_{max}(film)/cm^{-1} 1626 (C=O); **^{1}H NMR** (400 MHz, $CDCl_3$) δ_H 1.44 - 1.60 (m, 4H, $NCH_2CH_2C\underline{H}_2C\underline{H}_2$), 1.63 – 1.73 (m, 2H, $NCH_2C\underline{H}_2CH_2CH_2$), 1.76 – 1.86 (m, 2H, $C(O)CH_2C\underline{H}_2$), 2.07 (quin, *J* = 6.5, 2H, $ClCH_2C\underline{H}_2$), 2.46 – 2.53 (m, 2H, $C(O)C\underline{H}_2$), 3.44 (t, *J* = 6.5, 2H, $ClCH_2CH_2C\underline{H}_2$), 3.48 - 3.53 (m, 2H, $NC\underline{H}_2CH_2CH_2CH_2$), 3.58 (t, 2H, *J* = 6.5, $ClC\underline{H}_2$); **^{13}C NMR** (100 MHz, $CDCl_3$) δ_C 24.3 ($NCH_2CH_2\underline{C}H_2CH_2$), 26.3 ($NCH_2CH_2CH_2\underline{C}H_2$), 28.7 ($C(O)CH_2\underline{C}H_2$), 29.3 ($NCH_2\underline{C}H_2CH_2CH_2$), 30.9 ($ClCH_2\underline{C}H_2$), 34.0 ($C(O)\underline{C}H_2$), 42.9 ($Cl\underline{C}H_2$), 43.3 ($ClCH_2CH_2\underline{C}H_2$), 47.7 ($N\underline{C}H_2CH_2CH_2CH_2$), 175.2 ($\underline{C}(O)$); ***m/z*** (ESI$^+$) 204 ([M+H]$^+$); **HRMS** (ES$^+$) exact mass calculated for [M+H]$^+$ ($C_{10}H_{19}ONCl^+$) requires *m/z* 204.11497 and 206.11202, found *m/z* 204.11486 and 206.11187.

3.2 General procedure B for the synthesis of nitrated *N*-alkylated lactams 3

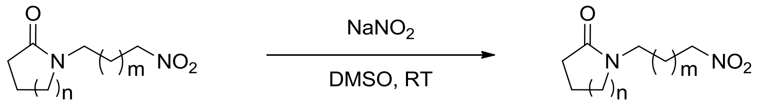

To a stirred solution of **11** (1.0 eq.) in DMSO (0.5 M) under an inert atmosphere was added solid $NaNO_2$ (2.0 eq.). The resulting mixture was stirred for 3 h (or until the reaction was complete by TLC analysis) at room temperature then quenched with water. The organic phase was separated and the aqueous layer was extracted (EtOAc). The

combined organic extracts were washed (brine), dried ($MgSO_4$), filtered and concentrated *in vacuo*. The residue was purified by FCC to yield the corresponding nitrated lactams **3**.

3.2.1 Preparation and characterisation of 1-(4-Nitrobutyl)pyrrolidin-2-one (3b)

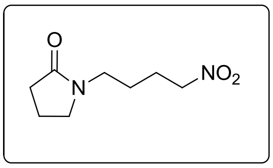

Prepared according to general procedure **B** using **11b** (0.191 g, 0.868 mmol). Purification by FCC yielded nitrated lactam **3b** (0.063 g, 39%) as a yellow oil. **IR** ν_{max}(film)/cm^{-1} 1668 (C=O), 1547 (NO_2), 1383 (NO_2); **^{1}H NMR** (400 MHz, $CDCl_3$) δ_H 1.62 ("quin", *J* = 7.5, 2H, $C\underline{H_2}(CH_2)_2NO_2$), 1.92 - 2.10 (m, 4H, $C\underline{H_2}CH_2NO_2$, $C(O)CH_2C\underline{H_2}$), 2.38 (t, *J* = 8.0, 2H, $C(O)C\underline{H_2}$), 3.26 - 3.46 (m, 4H, $C\underline{H_2}(CH_2)_3NO_2$, $C(O)(CH_2)_2C\underline{H_2}$), 4.43 (t, *J* = 6.5, 2H, $C\underline{H_2}NO_2$); **^{13}C NMR** (100 MHz, $CDCl_3$) δ_C 17.9 ($C(O)CH_2\underline{C}H_2$), 23.9 ($\underline{C}H_2(CH_2)_2NO_2$), 24.4 ($\underline{C}H_2CH_2NO_2$), 30.9 ($C(O)(CH_2)_2\underline{C}H_2$), 41.3 ($\underline{C}H_2(CH_2)_3NO_2$), 47.0 ($C(O)\underline{C}H_2$), 74.9 ($\underline{C}H_2NO_2$), 175.3 ($\underline{C}O$); ***m/z*** (ESI$^+$) 187 $[M+H]^+$; **HRMS** (ES$^+$) exact mass calculated for $[M+Na]^+$ ($C_8H_{14}N_2NaO_3^+$) requires *m/z* 209.0897, found *m/z* 209.0897.

3.2.2 Preparation and characterisation of 1-(4-Nitrobutyl)azepan-2-one (3a)

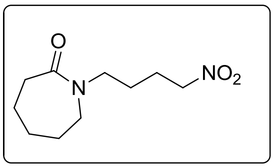

Prepared according to general procedure **B** using **11a** (5.00 g, 20.2 mmol). Purification by FCC yielded nitrated lactam **3a** (2.61 g, 60%) as a yellow oil. **IR** ν_{max}(film)/cm^{-1} 1633 (C=O), 1548 (NO_2), 1377 (NO_2); **^{1}H NMR** (400 MHz, $CDCl_3$) δ_H 1.52 - 1.76 (m, 8H, $C\underline{H_2}(CH_2)_2NO_2$, $C(O)CH_2C\underline{H_2}$, $C(O)(CH_2)_2C\underline{H_2}$, $C(O)(CH_2)_3C\underline{H_2}$), 1.91 - 2.03 (m, 2H,

$C\underline{H}_2CH_2NO_2$), 2.45 - 2.52 (m, 2H, $C(O)C\underline{H}_2$), 3.27 - 3.34 (m, 2H, $C(O)(CH_2)_4C\underline{H}_2$), 3.39 (t, J = 7.0, 2H, $C\underline{H}_2(CH_2)_3NO_2$), 4.42 (t, J = 6.5, 2H, $C\underline{H}_2NO_2$); **^{13}C NMR** (100 MHz, $CDCl_3$) δ_C 23.4, 24.5 ($\underline{C}H_2CH_2NO_2$), 24.7, 28.6, 29.9, 37.2 ($C(O)\underline{C}H_2$), 46.7 ($\underline{C}H_2(CH_2)_3NO_2$), 49.5 ($C(O)(CH_2)_4\underline{C}H_2$), 75.1 ($\underline{C}H_2NO_2$), 176.0 ($\underline{C}O$); ***m/z*** ($ESI^+$) 215 $[M+H]^+$; **HRMS** (ES^+) exact mass calculated for $[M+Na]^+$ ($C_{10}H_{18}N_2NaO_3^+$) requires *m/z* 237.1210, found *m/z* 237.1214.

3.2.3 Preparation and characterisation of 1-(5-Nitropentyl)pyrrolidin-2-one (3g)

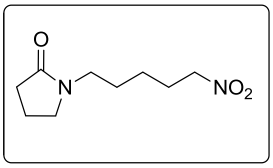

Prepared according to general procedure **B** using **11g** (1.89 g, 8.08 mmol). Purification by FCC yielded nitrated lactam **3g** (0.70 g, 43%) as a yellow oil. **IR** ν_{max}(film)/cm^{-1} 1672 (C=O), 1546 (NO_2), 1383 (NO_2); **^{1}H NMR** (400 MHz, $CDCl_3$) δ_H 1.28 - 1.41 (m, 2H, $C\underline{H}_2(CH_2)_2NO_2$), 1.48 - 1.61 (m, 2H, $C\underline{H}_2(CH_2)_3NO_2$), 1.90 - 2.08 (m, 4H, $C\underline{H}_2CH_2NO_2$, $C(O)CH_2C\underline{H}_2$), 2.33 (t, J = 8.0, 2H, $C(O)C\underline{H}_2$), 3.24 (t, J = 7.0, 2H, $C\underline{H}_2(CH_2)_4NO_2$), 3.32 (t, J = 7.0, 2H, $C(O)(CH_2)_2C\underline{H}_2$), 4.34 (t, J = 7.0, 2H, $C\underline{H}_2NO_2$); **^{13}C NMR** (100 MHz, $CDCl_3$) δ_C 17.7, 23.3 ($\underline{C}H_2(CH_2)_2NO_2$), 26.3 ($\underline{C}H_2(CH_2)_3NO_2$), 26.7, 30.8 ($C(O)\underline{C}H_2$), 41.7 ($\underline{C}H_2(CH_2)_4NO_2$), 46.9 ($C(O)(CH_2)_2\underline{C}H_2$), 75.3 ($\underline{C}H_2NO_2$), 174.9 ($\underline{C}O$); ***m/z*** ($ESI^+$) 201 $[M+H]^+$; **HRMS** (ES^+) exact mass calculated for $[M+Na]^+$ ($C_9H_{16}N_2NaO_3^+$) requires *m/z* 223.1053, found *m/z* 223.1061.

3.2.4 Preparation and characterisation of 1-(4-Nitrobutyl)piperidin-2-one (3c)

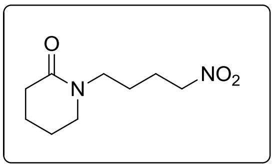

Prepared according to general procedure **B** using **11c** (2.00 g, 8.55 mmol). Purification by FCC yielded nitrated lactam **3c** (0.62 g, 36%) as a yellow oil. **IR** ν_{max}(film)/cm^{-1} 1624 (C=O), 1546 (NO_2), 1353 (NO_2); **^{1}H NMR** (500 MHz, $CDCl_3$) δ_H 1.65 ("dt", *J* = 14.5, 7.5, 2H, $C\underline{H_2}(CH_2)_2NO_2$), 1.74 - 1.86 (m, 4H, $C(O)CH_2C\underline{H_2}$, $C(O)(CH_2)_2C\underline{H_2}$), 1.95 - 2.06 (m, 2H, $C\underline{H_2}CH_2NO_2$), 2.38 (t, *J* = 6.5, 2H, $C(O)C\underline{H_2}$), 3.26 (t, *J* = 5.5, 2H, $C(O)(CH_2)_3C\underline{H_2}$), 3.43 (t, *J* = 7.0, 2H, $C\underline{H_2}(CH_2)_3NO_2$), 4.45 (t, *J* = 7.0, 2H, $C\underline{H_2}NO_2$); **^{13}C NMR** (125 MHz, $CDCl_3$) δ_C 21.3, 23.2, 23.7 ($\underline{C}H_2(CH_2)_2NO_2$), 24.5 ($\underline{C}H_2CH_2NO_2$), 32.3 ($C(O)\underline{C}H_2$), 45.7 ($\underline{C}H_2(CH_2)_3NO_2$), 47.8 ($C(O)(CH_2)_3\underline{C}H_2$), 75.1 ($\underline{C}H_2NO_2$), 170.0 ($\underline{C}O$); ***m/z*** (ESI$^+$) 201 [M+H]$^+$; **HRMS** (ES$^+$) exact mass calculated for [M+Na]$^+$ ($C_9H_{16}N_2NaO_3^+$) requires *m/z* 223.1053, found *m/z* 223.1060.

3.2.5 Preparation and characterisation of 1-(5-Nitropentyl)azepan-2-one (3f)

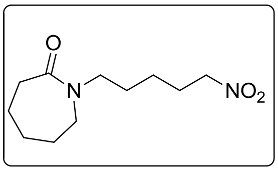

Prepared according to general procedure **B** using **11f** (1.64 g, 6.26 mmol). Purification by FCC yielded nitrated lactam **3f** (0.86 g, 60%) as a yellow oil. **IR** ν_{max}(film)/cm^{-1} 1634 (C=O), 1547 (NO_2), 1372 (NO_2); **^{1}H NMR** (400 MHz, $CDCl_3$) δ_H 1.34 - 1.45 (m, 2H, $C\underline{H_2}(CH_2)_2NO_2$), 1.52 - 1.81 (m, 8H, $C(O)CH_2C\underline{H_2}$, $C(O)(CH_2)_2C\underline{H_2}$, $C(O)(CH_2)_3C\underline{H_2}$, $C\underline{H_2}(CH_2)_3NO_2$), 2.00 - 2.11 (m, 2H, $C\underline{H_2}CH_2NO_2$), 2.48 - 2.56 (m, 2H, $C(O)C\underline{H_2}$), 3.31 - 3.35 (m, 2H, $C(O)(CH_2)_4C\underline{H_2}$), 3.38 (t, *J* = 7.5, 2H, $C\underline{H_2}(CH_2)_4NO_2$), 4.39 (t, *J* = 7.0, 2H, $C\underline{H_2}NO_2$); **^{13}C NMR** (100 MHz, $CDCl_3$) δ_C 23.3, 23.3, 26.8 ($\underline{C}H_2CH_2NO_2$), 27.1 ($\underline{C}H_2(CH_2)_3NO_2$), 28.5, 29.8, 37.1 ($C(O)\underline{C}H_2$), 47.4 ($\underline{C}H_2(CH_2)_4NO_2$), 49.4 ($C(O)(CH_2)_4\underline{C}H_2$), 75.3 ($\underline{C}H_2NO_2$), 175.6 ($\underline{C}O$); ***m/z*** (ESI$^+$) 229 [M+H]$^+$; **HRMS** (ES$^+$) exact mass calculated for [M+Na]$^+$ ($C_{11}H_{20}N_2NaO_3^+$) requires *m/z* 251.1366, found *m/z* 251.1365.

3.2.6 Preparation and characterisation of 1-(4-Nitrobutyl)azocan-2-one (3d)

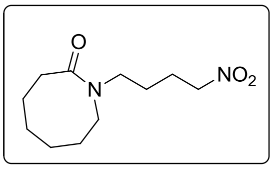

Prepared according to general procedure **B** using **11d** (1.31 g, 5.00 mmol). Purification by FCC yielded nitrated lactam **3d** (0.63 g, 55%) as a yellow. **IR** ν_{max}(film)/cm^{-1} 1622 (C=O), 1546 (NO_2), 1380 (NO_2); **^{1}H NMR** (400 MHz, $CDCl_3$) δ_H 1.41 - 1.56 (m, 4H, $C(O)(CH_2)_2C\underline{H_2}$, $C(O)(CH_2)_3C\underline{H_2}$), 1.57 - 1.69 (m, 4H, $C(O)(CH_2)_4C\underline{H_2}$, $C\underline{H_2}(CH_2)_2NO_2$), 1.71 - 1.83 (m, 2H, $C(O)CH_2C\underline{H_2}$), 1.93 - 2.05 (m, 2H, $C\underline{H_2}CH_2NO_2$), 2.41 - 2.51 (m, 2H, $C(O)C\underline{H_2}$), 3.34 (t, *J* = 7.0, 2H, $C\underline{H_2}(CH_2)_3NO_2$), 3.39 - 3.48 (m, 2H, $C(O)(CH_2)_5C\underline{H_2}$), 4.42 (t, *J* = 7.0, 2H, $C\underline{H_2}NO_2$); **^{13}C NMR** (100 MHz, $CDCl_3$) δ_C 24.3, 24.5, 24.7, 26.2, 28.7, 29.2, 33.9 ($C(O)\underline{C}H_2$), 43.9 ($\underline{C}H_2(CH_2)_3NO_2$), 46.9 ($C(O)(CH_2)_5\underline{C}H_2$), 75.2 ($\underline{C}H_2NO_2$), 175.1 ($\underline{C}O$); ***m/z*** (ESI$^+$) 229 [M+H]$^+$; **HRMS** (ES$^+$) exact mass calculated for [M+Na]$^+$ ($C_{11}H_{20}N_2NaO_3^+$) requires *m/z* 251.1366, found *m/z* 251.1365.

3.3 General procedure C for the synthesis of nitrated N-alkylated lactams 3e and 5k

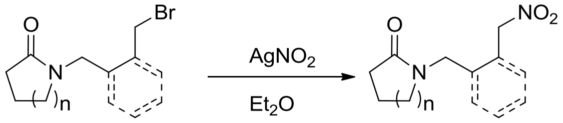

To a stirred solution of **11** (1.0 eq.) in diethylether (1.9M) with the exclusion of light (in a foil covered flask) under an inert atmosphere was added $AgNO_2$ (4.0 eq.). The resulting mixture was stirred for 5 days at room temperature. The mixture was then filtered, washing the filter cake with diethylether. The filtrate was concentrated *in vacuo* and purified by FCC to yield the corresponding nitrated lactams **3**.

3.3.1 Preparation and characterisation of 4-(4-nitrobutyl)-1,4-oxazepan-5-one (3e)

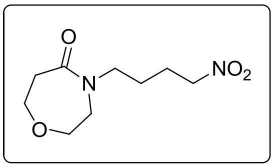

Prepared according to general procedure **C** using **11e** (1.5 g, 6.0 mmol). Purification by FCC yielded nitrated lactam **3e** (0.55 g, 42%) as a clear colourless oil. **IR** ν_{max}(film)/cm^{-1} 1640 (C=O), 1548 (NO_2), 1372 (NO_2); **^{1}H NMR** (400 MHz, $CDCl_3$) δ_H 1.57 - 1.67 (m, 2H), 1.98 – 2.07 (m, 2H), 2.75 – 2.79 (m, 2H $C\underline{H}_2C(O)$), 3.44 – 3.48 (m, 4H, $OCH_2C\underline{H}_2N$, $C\underline{H}_2(CH_2)_3NO_2$), 3.73 – 3.78 (m, 2H), 3.78 – 3.81 (m, 2H), 4.44 – 4.49 (t, *J* = 7.0, 2H, $C\underline{H}_2NO_2$); **^{13}C NMR** (100 MHz, $CDCl_3$) δ_C 24.4, 24.7, 41.2 ($C(O)\underline{C}H_2$), 47.3, 51.6, 65.5, 70.5, 75.0 ($\underline{C}H_2NO_2$), 174.5 ($\underline{C}O$); ***m/z*** (ESI$^+$) 217 ([M+H]$^+$); **HRMS** (ES$^+$) exact mass calculated for [M+Na]$^+$ ($C_9H_{16}N_2NaO_4^+$) requires *m/z* 239.1002, found *m/z* 239.1012.

3.3.2 Preparation and characterisation of 1-[2-(nitromethyl)benzyl]azepan-2-one (3k)

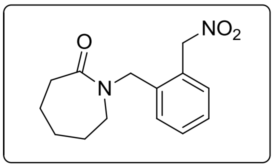

Prepared according to general procedure **C** using **11k** (2.00 g, 6.27 mmol). Purification by FCC yielded nitrated lactam **3k** (0.43 g, 24%) as a yellow oil. **IR** ν_{max}(film)/cm^{-1} 1620 (C=O), 1551 (NO_2), 1370 (NO_2); **^{1}H NMR** (400 MHz, $CDCl_3$) δ_H 1.52 - 1.59 (m, 2H), 1.67 - 1.76 (m, 4H), 2.56 – 2.61 (m, 2H, $C(O)C\underline{H}_2$), 3.23 – 3.27 (m, 2H, $NC\underline{H}_2CH_2$), 4.72 (s, 2H, $NC\underline{H}_2Ar$), 5.68 (s, 2H, $ArC\underline{H}_2NO_2$), 7.31 – 7.34 (m, 1H), 7.37-7.46 (m, 3H); **^{13}C NMR** (100 MHz, $CDCl_3$) δ_C 23.2, 27.6, 29.8, 36.8 ($CO\underline{C}H_2$), 48.1 ($N\underline{C}H_2Ar$), 48.4

($N\underline{C}H_2CH_2$), 76.7 ($NO_2\underline{C}H_2$), 128.5 ($Ar\underline{C}H$), 128.9 ($Ar\underline{C}H$), 130.3 ($Ar\underline{C}H$), 130.6 ($Ar\underline{C}H$), 132.4 ($Ar\underline{C}_{quat}$), 137.7 ($Ar\underline{C}_{quat}$), 176.0 ($\underline{C}O$); ***m/z*** (ESI^+) 263 ($[M+H]^+$); **HRMS** (ES^+) exact mass calculated for $[M+Na]^+$ ($C_{14}H_{18}N_2NaO_3^+$) requires *m/z* 285.1210, found *m/z* 285.1210.

3.4 General procedure D for the synthesis of nitrated *N*-alkylated lactams 3h and 3i

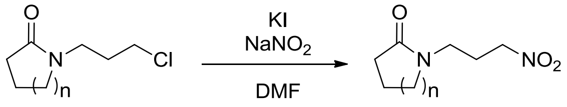

To a stirred solution of **11** (1.0 eq.) in DMSO (1.2 M) under an inert atmosphere was added solid $NaNO_2$ (2.4 eq.) and KI (0.05 eq). The resulting mixture was stirred at room temperature until the reaction was complete. The reaction was then quenched with water and extracted with EtOAc. The combined organic extracts were washed with brine, dried ($MgSO_4$), filtered and concentrated *in vacuo*. The residue was purified by FCC to yield the corresponding nitrated lactams **3h-3i.**

3.4.1 Preparation and characterisation of 1-(3-nitropropyl)azepan-2-one (3h)

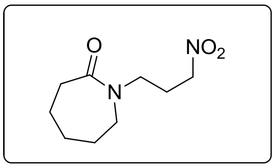

Prepared according to general procedure **D** using **11h** (1.6 g, 8.5 mmol). Purification by FCC yielded nitrated lactam **3h** (0.35 g, 21%) as a yellow oil. **IR** ν_{max}(film)/cm^{-1} 1635 (C=O), 1549 (NO_2), 1369 (NO_2); ^{1}**H NMR** (400 MHz, C_6D_6) δ_H 0.98 – 1.04 (m, 2H, $NCH_2C\underline{H_2}CH_2CH_2$), 1.14 – 1.20 (m, 2H, $C(O)CH_2CH_2C\underline{H_2}$), 1.23 – 1.29 (m, 2H, $C(O)CH_2C\underline{H_2}$), 1.65 (“quin”, 2H, J = 7.0, $NO_2CH_2C\underline{H_2}$), 2.19 – 2.22 (m, 2H, $C(O)C\underline{H_2}$),

2.51 – 2.53 (m, 2H, $NC\underline{H_2}CH_2CH_2CH_2$), 3.00 (t, *J* = 7.0, 2H, $C(O)NC\underline{H_2}CH_2CH_2NO_2$), 3.81 (t, *J* = 7.0, 2H, $NO_2C\underline{H_2}$); **^{13}C NMR** (400 MHz, C_6D_6) δ_C 23.9 ($C(O)CH_2\underline{C}H_2$), 26.6 ($NO_2CH_2\underline{C}H_2$), 29.2 ($NCH_2\underline{C}H_2CH_2CH_2$), 30.2 ($C(O)CH_2CH_2\underline{C}H_2$), 37.4 ($C(O)\underline{C}H_2$), 45.4 ($NO_2CH_2CH_2\underline{C}H_2$), 49.5 ($C(O)N\underline{C}H_2CH_2CH_2CH_2$), 73.5 ($NO_2\underline{C}H_2$), 175.4 ($\underline{C}(O)$); ***m/z*** ($ESI^+$) 201 ($[M+H]^+$); **HRMS** ($ES^+$) exact mass calculated for $[M+Na]^+$ ($C_9H_{16}N_2NaO_3^+$) requires *m/z* 223.1053 and 224.1086 found *m/z* 223.1062 and 224.1091.

3.4.2 Preparation and characterisation of 1-(3-nitropropyl)azocan-2-one (3i)

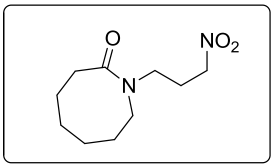

Prepared according to general procedure **D** using **11i** (1.0 g, 4.9 mmol). Purification by FCC yielded nitrated lactam **3i** (0.35 g, 33%) as a yellow oil. **IR** ν_{max}(film)/cm^{-1} 1626 (C=O), 1547 (NO_2), 1377 (NO_2); **^{1}H NMR** (400 MHz, $CDCl_3$) δ_H 1.44 - 1.59 (m, 4H, $C(O)CH_2CH_2C\underline{H_2}C\underline{H_2}$), 1.63 - 1.71 (m, 2H, $C(O)NCH_2C\underline{H_2}CH_2CH_2$), 1.76 - 1.85 (m, 2H, $C(O)CH_2C\underline{H_2}$), 2.25-2.34 (m, 2H, $NO_2CH_2C\underline{H_2}$), 2.46 – 2.53 (m, 2H, $C(O)C\underline{H_2}$), 3.42 (t, 2H, *J* = 6.5, $NO_2CH_2CH_2C\underline{H_2}$), 3.48 (t, 2H, *J* = 5.9, $C(O)NC\underline{H_2}CH_2CH_2CH_2$), 4.45 (t, 2H, *J* = 7.0, $NO_2C\underline{H_2}$); **^{13}C NMR** (100 MHz, $CDCl_3$) δ_C 24.2, 25.9 ($NO_2CH_2\underline{C}H_2$), 26.2, 28.7 ($C(O)CH_2\underline{C}H_2$), 29.2 ($NCH_2\underline{C}H_2CH_2CH_2$), 33.9 ($C(O)\underline{C}H_2$), 42.5 ($NO_2CH_2CH_2\underline{C}H_2$), 47.5 ($N\underline{C}H_2CH_2CH_2CH_2$), 73.5 ($NO_2\underline{C}H_2$), 175.6 ($\underline{C}(O)$); ***m/z*** ($ESI^+$) 215.13902 ($[M+H]^+$); **HRMS** ($ES^+$) exact mass calculated for $[M+H]^+$ ($C_{10}H_{19}O_3N_2^+$) requires *m/z* 215.13902 and 216.14237, found *m/z* 215.13888 and 216.14224.

3.5 General procedure E for the synthesis of nitrated N-alkylated lactams 3j and 3l

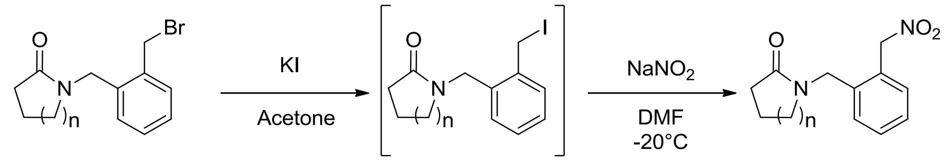

To a stirred solution of **11** (1.0 eq.) in acetone (1.2 M) under an inert atmosphere was added sodium iodide (2.0 eq.). The resulting mixture was stirred for 24 h at room temperature with the exclusion of light (in an aluminium foil covered flask). The mixture was concentrated *in vacuo* then dissolved in diethyl ether and washed with water. The organic layer was concentrated to afford the crude iodide, which was used without purification. The iodide was dissolved in DMF (0.25 M) under nitrogen. Sodium nitrite (3 eq) was added and the reaction was cooled to – 20 °C and stirred until the reaction was complete by ^{1}HNMR analysis. The reaction was diluted with EtOAc, washed with brine and dried over Na_2SO_4. The organic layer was concentrated *in vacuo* and purified by FCC to yield the corresponding nitrated lactams **3j** and **3l**.

3.5.1 Preparation and characterisation of 1-[2-(nitromethyl)benzyl]piperidin-2-one (3j)

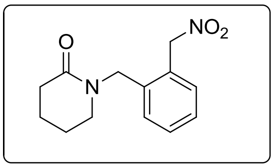

Prepared according to general procedure **E** using **11j** (1.77 g, 6.27 mmol) crude iodide was obtained in (1.9 g, 92%). The iodide (1 g, 3 mmol) was used according to procedure C. Purification by FCC (Et_2O) yielded nitrated lactam **3j** (0.2 g, 27%) as a yellow oil. **IR** ν_{max}(film)/cm^{-1} 1616 (C=O), 1548 (NO_2), 1374 (NO_2); **^{1}H NMR** (400 MHz, $CDCl_3$) δ_H 1.63 – 1.72 (m, 4H, C(O)CH$_2$C$\underline{H_2}$, C(O)CH$_2$CH$_2$C$\underline{H_2}$), 2.35 (t, *J* = 6.0, 2H, C(O)C$\underline{H_2}$), 3.04 (t, 2H, NC$\underline{H_2}$CH$_2$), 4.67 (s, 2H, NC$\underline{H_2}$Ar), 5.59 (s, 2H, NO$_2$CH$_2$), 7.22 – 7.25 (m,

1H), 7.29 – 7.38 (m, 3H); **^{13}C NMR** (100 MHz, $CDCl_3$) δ_C 21.1, 22.8, 32.2 ($C(O)\underline{C}H_2$), 47.2 ($N\underline{C}H_2CH_2$), 47.9 ($N\underline{C}H_2Ar$), 76.7 ($NO_2\underline{C}H_2$), 128.6 (ArCH), 129.0 (ArCH), 130.2 (ArCH), 130.7 (ArCH), 132.6 (ArC_{quat}), 137.2 (ArC_{quat}), 169.8 ($\underline{C}O$); ***m/z*** (ESI^+) 249 ($[M+H]^+$) **HRMS** (ES^+) exact mass calculated for $[M+H]^+$ ($C_{13}H_{17}O_3N_2^+$) requires *m/z* 249.12337 and 250.12672, found *m/z* 249.12327 and 250.12656.

3.5.2 Preparation and characterisation of 1-[2-(nitromethyl)benzyl]azocan-2-one (3l)

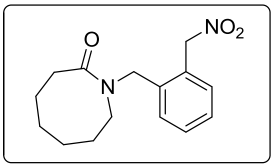

Prepared according to general procedure **E** using **11l** (2.0 g, 6.5 mmol). Purification by FCC (Et_2O) yielded nitrated lactam **3l** (0.36 g, 20%) as a white solid. (**m.p.** = 90-95 °C). **IR** ν_{max}(film)/cm^{-1} 1626 (C=O), 1542 (NO_2), 1363 (NO_2); **^{1}H NMR** (500 MHz, C_6D_6) δ_H 1.03 - 1.18 (m, 6H, $NCH_2C\underline{H_2}C\underline{H_2}C\underline{H_2}$), 1.54 – 1.61 (m, 2H, $C(O)CHC\underline{H_2}$), 2.29 – 2.35 (m, 2H, $C(O)C\underline{H_2}CH_2$), 2.76 – 2.81 (m, 2H, $NC\underline{H_2}CH_2$), 4.49 (br s, 2H, $C(O)NC\underline{H_2}Ar$), 5.16 (s, 2H, $NO_2C\underline{H_2}Ar$), 6.86 (br d, *J* = 7.5, 1H, Ar$\underline{H}$), 6.91 (br d, *J* = 7.5, 1H, Ar$\underline{H}$), 6.95 (td, *J* = 7.5, 1.0, 1H, Ar$\underline{H}$), 7.01 (td, *J* = 7.5, 1.0, 1H, Ar$\underline{H}$); **^{13}C NMR** (125 MHz, C_6D_6) δ_C 24.6, 26.6, 28.3, 29.3 $C(O)CH_2\underline{C}H_2$), 33.9 ($C(O)\underline{C}H_2CH_2$), 44.6 ($N\underline{C}H_2CH_2$), 44.7 ($C(O)N\underline{C}H_2Ar$), 76.7 ($NO_2\underline{C}H_2Ar$), 128.0 – 180.7 (Ar$\underline{C}$H, hidden under C_6H_6 peak, assigned with the help of HSQC), 129.9 (Ar$\underline{C}$H), 130.1 (Ar$\underline{C}$H), 130.8 (Ar$\underline{C}$H), 133.3 ($Ar\underline{C}_{quat}$), 138.7 ($Ar\underline{C}_{quat}$), 174.7 ($\underline{C}$(O)); ***m/z*** (ESI^+) 277 ($[M+H]^+$); **HRMS** (ES^+) exact mass calculated for $[M+H]^+$ ($C_{15}H_{21}O_3N_2^+$) requires *m/z* 277.15467 and 278.15802, found *m/z* 277.15442 and 278.15787.

3.6 General procedure F for the synthesis of nitrated bicycles 5.

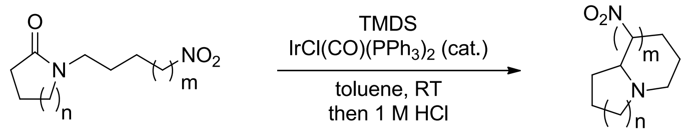

To a stirred solution of **3** (1.0 eq.) in toluene (0.01 M) under an inert atmosphere at room temperature was added TMDS (2.0 eq.) and $IrCl(CO)(PPh_3)_2$ (0.005 eq.). The resulting solution was stirred until complete conversion of the starting material (TLC) then quenched with 1 M HCl (12 mL/mmol **3**). The aqueous layer was separated and the organic layer was extracted (1 M HCl, 3 × 12 mL/mmol **3**). The combined aqueous extracts were washed (Et_2O, 3 × 6 mL/mmol **3**) and basified to pH 10 (K_2CO_3). The aqueous layer was then extracted (Et_2O, 3 × 6 mL/mmol **3**), the organic phases combined, dried ($MgSO_4$), filtered and concentrated *in vacuo*. The residue was purified by FCC to yield the title compound **5.**

3.6.1 Preparation and characterisation of (R*,S*)-8-nitrooctahydroindolizine (5b)

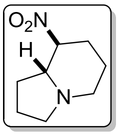

Prepared according to general procedure **F** using **3b** (0.100 g, 0.537 mmol). Purification by FCC to yield the title compound **5b** (0.022 g, 37%, d.r. 95:5) as a yellow oil. **IR** ν_{max}(film)/cm^{-1} 1544 (NO_2), 1375 (NO_2); **^{1}H NMR** (500 MHz, $CDCl_3$) δ_H 1.58 - 1.78 (m, 3H), 1.78 - 1.91 (m, 3H), 1.95 - 2.02 (m, 1H, C$\underline{H_a}$H$_b$CHCHNO$_2$), 2.09 ("td", *J* = 11.5, 2.5, 1H, NC$\underline{H_a}$H$_b$), 2.26 ("q", *J* = 9.0, 1H, NC$\underline{H_c}$H$_d$), 2.30 - 2.37 (m, 2H, NC$\underline{H}$(C)$_2$), 3.02 - 3.15 (m, 2H, NCH$_a\underline{H_b}$, NCH$_c\underline{H_d}$), 4.26 (ddd, *J* = 11.5, 9.5, 4.0, 1H, C$\underline{H}$NO$_2$); **^{13}C NMR** (125 MHz, $CDCl_3$) δ_C 20.6, 24.0, 28.2, 30.1 ($\underline{C}$H$_2$CHCHNO$_2$), 51.2 (N$\underline{C}$H$_a$H$_b$), 53.9 (N$\underline{C}$H$_c$H$_d$), 65.7 ($\underline{C}$HCHNO$_2$), 87.9 ($\underline{C}$HNO$_2$); ***m/z*** (ESI$^+$) 171 [M+H]$^+$; **HRMS** (ES$^+$)

exact mass calculated for $[M+H]^+$ ($C_8H_{15}N_2O_2^+$) requires *m/z* 171.1128, found *m/z* 171.1124.

3.6.2 Preparation and characterisation of (R*,S*)-1-nitrooctahydro-2H-quinolizine (5c)

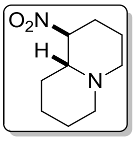

Prepared according to general procedure **F** using **3c** (0.0630 g, 0.315 mmol). Purification by FCC to yield the title compound **5c** (0.026 g, 45%, d.r. >98:2) as a yellow oil. **IR** ν_{max}(film)/cm^{-1} 1543 (NO_2), 1371 (NO_2); **^{1}H NMR** (400 MHz, $CDCl_3$) δ_C 1.18 - 1.37 (m, 2H), 1.48 - 1.86 (m, 6H), 1.95 ("qd", *J* = 12.5, 4.5, 1H, $C\underline{H_a}H_bCHNO_2$), 2.08 - 2.20 (m, 2H, $NC\underline{H_a}CH_b$, $NCH_a\underline{H_b}$), 2.22 - 2.32 (m, 2H, $CH_a\underline{H_b}CHNO_2$, $C\underline{H}CHNO_2$), 2.79 ("d", *J* = 12.0, 1H, $NC\underline{H_c}CH_d$), 2.88 ("d", *J* = 11.5, 1H, $NCH_cC\underline{H_d}$), 4.30 (ddd, *J* = 12.0, 9.5, 4.0, 1H, $C\underline{H}NO_2$); **^{13}C NMR** (100 MHz, $CDCl_3$) δ_C 23.0, 23.5, 25.3, 28.8, 30.5 ($\underline{C}H_2CHNO_2$), 55.3 ($N\underline{C}H_2$), 56.1 ($N\underline{C}H_2$), 64.0 ($\underline{C}HCHNO_2$), 89.4 ($\underline{C}HNO_2$); ***m/z*** (ESI$^+$) 185 ($[M+H]^+$). All data in agreement with that previously reported in the literature.[3]

3.6.3 Preparation and characterisation of (R*,S*)-1-nitrodecahydropyrido[1,2-*a*]azepine (5a)

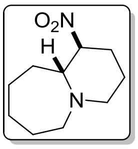

Prepared according to general procedure **F** using **3a** (0.100 g, 0.467 mmol). Purification by FCC to yield the title compound **5a** (0.074 g, 80%, d.r. 88:12) as a yellow oil. **IR** ν_{max}(film)/cm^{-1} 1543 (NO_2), 1375 (NO_2); **^{1}H NMR** (400 MHz, $CDCl_3$) – major

diastereomer: 1.44 - 1.75 (m, 10H), 1.94 (qd, *J* = 12.5, 5.0, 1H, $C\underline{H_a}H_bCHNO_2$), 2.19 - 2.29 (m, 1H, $CH_a\underline{H_b}CHNO_2$), 2.57 (td, *J* = 12.0, 3.5, 1H, $NC\underline{H_a}H_b$), 2.62 - 2.87 (m, 4H, $NCH_a\underline{H_b}$, $NC\underline{H_c}\underline{H_d}$, $C\underline{H}CHNO_2$), 4.36 (ddd, *J* = 11.5, 9.5, 4.0, 1H, $C\underline{H}NO_2$); minor diastereomer observable: 2.00 - 2.09 (m, 1H), 2.46 - 2.52 (m, 1H), 2.88 - 2.96 (m, 1H), 3.26 - 3.36 (m, 1H), 4.51 - 4.63 (m, 1H); **^{13}C NMR** (100 MHz, $CDCl_3$) δ_C – major diastereomer: 23.4, 23.8, 28.2, 28.6, 29.7, 30.5, 54.8 ($N\underline{C}H_2$), 55.7 ($N\underline{C}H_2$), 65.2 ($\underline{C}HCHNO_2$), 88.0 ($\underline{C}HNO_2$); minor diastereomer observable: 23.2, 24.1, 25.2, 25.3, 25.9, 27.6, 48.3 ($N\underline{C}H_2$), 56.5 ($N\underline{C}H_2$), 62.7 ($\underline{C}HCHNO_2$), 85.6 ($\underline{C}HNO_2$); ***m/z*** (ESI^+) 199 ($[M+H]^+$); **HRMS** (ES^+) exact mass calculated for $[M+H]^+$ ($C_{10}H_{19}N_2O_2^+$) requires *m/z* 199.1441, found *m/z* 199.1437.

Prepared according to general procedure **F** using **3a** (2.00 g, 9.35 mmol). Purification of crude mixture (d.r. 88:12) by FCC to yield the title compound **5a** (1.31 g, 71%, single diastereomer, the minor diastereomer decomposed during FCC). **^{1}H NMR** (400 MHz, $CDCl_3$) δ_H 1.43 - 1.76 (m, 10H), 1.93 (“qd”, *J* = 12.5, 5.0, 1H, $C\underline{H_a}H_bCHNO_2$), 2.17 - 2.29 (m, 1H, $CH_a\underline{H_b}CHNO_2$), 2.56 (“td”, *J* = 12.0, 3.0, 1H, $NC\underline{H_a}H_b$), 2.62 - 2.88 (m, 4H, $NCH_a\underline{H_b}$, $NC\underline{H_c}\underline{H_b}$, $C\underline{H}CHNO_2$), 4.35 (ddd, *J* = 11.5, 9.5, 4.0, 1H, $C\underline{H}NO_2$); **^{13}C NMR** (100 MHz, $CDCl_3$) δ_C 23.4, 23.8, 28.2, 28.6, 29.7, 30.5, 54.7 ($N\underline{C}H_2$), 55.7 ($N\underline{C}H_2$), 65.1 ($\underline{C}HCHNO_2$), 88.0 ($\underline{C}HNO_2$).

3.6.4 Preparation and characterisation of (R*,S*)-1-nitrodecahydro-2*H*-pyrido[1,2-*a*]azocine (5d)

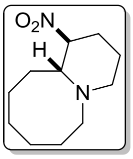

Prepared according to general procedure **F** using **3d** (0.100 g, 0.439 mmol). Purification by FCC to yield the title compound **5d** (0.069 g, 74%, d.r. 93:7) as a yellow oil. **IR** ν_{max}(film)/cm^{-1} 1542 (NO_2), 1373 (NO_2); **^{1}H NMR** (500 MHz, C_6D_6) – major diastereomer: δ_H 1.05 - 1.72 (m, 12H), 1.73 - 1.86 (m, 2H, $C\underline{H_2}CHNO_2$), 2.08 - 2.22 (m, 1H, $NC\underline{H_a}H_b$), 2.24 - 2.39 (m, 2H, $NC\underline{H_c}\underline{H_d}$), 2.40 - 2.48 (m, 1H, $NCH_a\underline{H_b}$), 2.87 (ddd, *J* = 9.5, 6.5, 3.0, 1H, $C\underline{H}CHNO_2$), 4.25 (“td”, *J* = 10.5, 5.0, 1H, $C\underline{H}NO_2$); minor diastereomer observable: 2.66 - 2.72 (m, 2H), 4.08 - 4.15 (m, 1H); **^{13}C NMR** (125 MHz, C_6D_6) δ_C – major diastereomer: 22.8, 26.2, 26.3, 26.7, 27.5, 29.4, 31.0 ($\underline{C}H_2CHNO_2$), 49.8, 53.9, 64.5 ($\underline{C}HCHNO_2$), 86.8 ($\underline{C}HNO_2$); ***m/z*** (ESI$^+$) 213 ([M+H]$^+$); **HRMS** (ES$^+$) exact mass calculated for [M+H]$^+$ ($C_{11}H_{21}N_2O_2^+$) requires *m/z* 213.1598, found *m/z* 213.1597.

3.6.5 Preparation and characterisation of (R*,S*)-10-nitrooctahydro-2*H*-pyrido[1,2-*d*][1,4]oxazepine (5e)

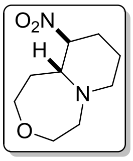

Prepared according to general procedure **F** using **3e** (0.095 g, 0.44 mmol). Purification by FCC to yield the title compound **5e** (0.045 mg, 51%, d.r. 91:9) as a yellow oil. **IR** ν_{max}(film)/cm^{-1} 1540 (NO_2), 1374 (NO_2); **^{1}H NMR** (400 MHz, $CDCl_3$) – major diastereomer: δ_H 1.59 - 1.70 (m, 1H, $NO_2CHCH_2CH_a\underline{H_b}$), 1.72 - 1.79 (m, 1H, $NO_2CHCH_2C\underline{H_a}H_b$), 1.80 – 1.86 (m, 2H, $OCH_2C\underline{H_2}CH$), 1.89 – 2.00 (m, 1H, $NO_2CHCH_a\underline{H_b}$), 2.23 - 2.29 (m, 1H, $NO_2CHC\underline{H_a}H_b$), 2.53 (“td”, *J* = 12.0, 3.0, 1H, $NCH_a\underline{H_b}CH_2CH_2$), 2.79 – 2.95 (m, 4H, $NC\underline{H_a}H_bCH_2CH_2$, $NC\underline{H}CHNO_2$, $NC\underline{H_a}\underline{H_b}CH_2O$),

3.68 – 3.76 (m, 3H, $OCH_a\underline{H_b}CH_2CH$, $O\underline{CH_aH_b}CH_2N$), 3.80 – 3.86 (m, 1H, $OC\underline{H_a}H_bCH_2CH$), 4.35 (ddd, *J* = 11.5, 9.5, 4.5, 1H, $NO_2C\underline{H}$); minor diastereomer observable: 1.42 – 1.49 (m, 1H), 3.03 – 3.10 (m, 1H), 3.45 – 3.52 (m, 1H), 4.59 – 4.64 (m, 1H, $NO_2C\underline{H}$); **^{13}C NMR** (100 MHz, $CDCl_3$) δ_C – major diastereomer: 23.2 $NCH_2\underline{C}H_2CH_2$), 29.9 $NO_2CH\underline{C}H_2$), 32.7 ($OCH_2\underline{C}H_2CH$), 55.3 ($N\underline{C}H_2CH_2CH_2$), 57.8 ($N\underline{C}H_2CH_2O$), 63.7 ($N\underline{C}HCHNO_2$), 65.5 ($O\underline{C}H_2CH_2N$), 69.2 ($O\underline{C}H_2CH_2CH$), 87.9 ($NO_2\underline{C}H$); minor diastereomer observable: 23.6, 27.7, 56.6, 61.0, 66.3, 67.6, 84.9 ($NO_2\underline{C}H$); ***m/z*** (ESI^+) 201 ($[M+H]^+$); **HRMS** (ESI^+) exact mass calculated for $[M+H]^+$ ($C_9H_{17}N_2O_3^+$) requires *m/z* 201.1234 and 202.1267, found *m/z* 201.1233 and 202.1260.

3.6.6 Preparation and characterisation of (R*,S*)-1-nitrodecahydro-1*H*-azepino[1,2-*a*]azepine (5f)

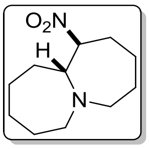

Prepared according to general procedure **F** using **3f** (0.100 g, 0.439 mmol). Purification by FCC to yield the title compound **5f** (0.061 g , 65%, d.r. 86:14) as a yellow oil. **IR** ν_{max}(film)/cm^{-1} 1539 (NO_2), 1358 (NO_2); **^{1}H NMR** (400 MHz, $CDCl_3$) δ_H 1.13 - 2.18 (m, 14H), 2.71 - 2.99 (m, 4H, 2 × $NC\underline{H_2}$), 3.17 - 3.31 (m, 1H, $C\underline{H}CHNO_2$), 4.35 ("dt", *J* = 9.5, 6.0, 1H, $C\underline{H}NO_2$); minor diastereomer observable: 1.13 - 2.18 (m, 13H), 2.24 - 2.38 (m, 1H), 2.71 - 2.99 (m, $NC\underline{H_2}$), 3.11 ("dt", *J* = 12.0, 3.5, 1H, $C\underline{H}CHNO_2$), 4.57 (ddd, *J* = 8.5, 5.5, 3.5, 1H, $C\underline{H}NO_2$); **^{13}C NMR** (125 MHz, $CDCl_3$) δ_C – major diastereomer: 22.6, 26.4, 28.3, 29.2, 29.5, 31.7, 33.0, 50.8 ($N\underline{C}H_2$), 54.6 ($N\underline{C}H_2$), 65.9 ($\underline{C}HCHNO_2$), 92.5 ($\underline{C}HNO_2$); minor diastereomer observable: 22.7, 26.9, 28.9, 30.8, 31.0, 31.7, 52.6 ($N\underline{C}H_2$), 56.3 ($N\underline{C}H_2$), 65.8 ($\underline{C}HCHNO_2$), 91.7 ($\underline{C}HNO_2$); ***m/z*** (ESI^+) 213 ($[M+H]^+$);

HRMS (ES^+) exact mass calculated for $[M+H]^+$ ($C_{11}H_{21}N_2O_2^+$) requires *m/z* 213.1598, found *m/z* 213.1604.

3.6.7 Preparation and characterisation of (R*,*S**)-9-nitrooctahydro-1*H*-pyrrolo[1,2-*a*]azepine (5g)

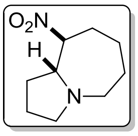

Prepared according to general procedure **F** using **3g** (0.100 g, 0.543 mmol). Purification by FCC to yield the title compound **5g** (0.044 g, 48%, d.r. 95:5) as a yellow oil. **IR** ν_{max}(film)/cm^{-1} 1544 (NO_2), 1378 (NO_2); **^{1}H NMR** (400 MHz, $CDCl_3$) – major diastereomer: δ_H 1.59 - 1.91 (m, 7H), 1.92 - 2.12 (m, 2H, $C\underline{H_a}H_bCHNO_2$, $C\underline{H_a}H_bCHCHNO_2$), 2.15 - 2.25 (m, 1H, $CH_a\underline{H_b}CHNO_2$), 2.40 - 2.54 (m, 2H, $NC\underline{H_a}H_b$, $NC\underline{H_c}H_d$), 2.96 - 3.07 (m, 2H, $NCH_a\underline{H_b}$, $NCH_c\underline{H_d}$), 3.13 ("td", *J* = 9.5, 4.0, 1H, $C\underline{H}CHNO_2$), 4.37 ("dt", *J* = 10.0, 6.0, 1H, $C\underline{H}NO_2$); minor diastereomer observable: 4.67 - 4.76 (m, 1H, $C\underline{H}NO_2$); **^{13}C NMR** (100 MHz, $CDCl_3$) – major diastereomer: δ_C 21.9, 23.5, 29.6, 30.3 ($\underline{C}H_2CHCHNO_2$), 33.5 ($\underline{C}H_2CHNO_2$), 55.1 ($N\underline{C}H_2$), 57.5 ($N\underline{C}H_2$), 65.3 ($\underline{C}HCHNO_2$), 92.0 ($\underline{C}HNO_2$); minor diastereomer observable: 22.9, 24.2, 29.0, 31.3, 56.6, 57.2, 64.9, 89.9; ***m/z*** (ESI^+) 185 ($[M+H]^+$); **HRMS** (ES^+) exact mass calculated for $[M+H]^+$ ($C_9H_{17}N_2O_2^+$) requires *m/z* 185.1285, found *m/z* 185.1277.

3.6.8 Preparation and characterisation of (R*,*S**)-1-nitrooctahydro-1*H*-pyrrolo[1,2-*a*]azepine (5h)

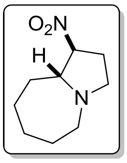

Prepared according to general procedure **F** using **3h** (0.81 g, 0.40 mmol). Purification by FCC to yield the title compound **5h** (0.049 g, 61%, d.r. 55:45) as a yellow oil. **IR** ν_{max}(film)/cm^{-1} 1546 (NO_2), 1376 (NO_2); **^{1}H NMR** (400 MHz, $CDCl_3$) – major diastereomer: δ_H 1.51 - 1.80 (m, 7H), 2.06 - 2.14 (m, 1H, NCH(C)C$\underline{H_a}$$H_b$), 2.22 - 2.44 (m, 3H, $NCH_2C\underline{H_a}\underline{H_b}CHNO_2$, $NC\underline{H_a}H_bCH_2CH_2$), 2.78 (ddd, *J* = 10.5, 9.5, 7.0, 1H, $NCH_a\underline{H_b}CH_2CHNO_2$), 2.90 (ddd, *J* = 10.5, 5.5, 3.0, 1H, $NC\underline{H}CHNO_2$), 3.01 – 3.14 (m, 2H, $NC\underline{H_a}H_bCH_2CHNO_2$, $NC\underline{H_a}H_bCH_2CH_2$), 4.58 (ddd, *J* = 8.5, 5.5, 3.0, 1H, $NO_2C\underline{H}$); minor diastereomer observable: 1.29 – 1.39 (m, 1H), 2.57 (“ddt”, *J* = 13.5, 9.0, 4.5, 1H), 2.65 (ddd, *J* = 10.0, 7.5, 2.5, 1H), 3.29 (“td”, *J* = 8.5, 2.5, 1H), 5.00 (“td”, *J* = 8.0, 4.5, 1H); **^{13}C NMR** (100 MHz, $CDCl_3$) major diastereomer: δ_C 25.4, 25.7, 28.2, 29.5 ($NO_2CH\underline{C}H_2CH_2N$), 34.1 (NCH(C)$\underline{C}H_2$), 54.8 ($N\underline{C}H_2CH_2CH_2$), 55.7 ($N\underline{C}H_2CH_2CHNO_2$), 70.8 ($N\underline{C}HCHNO_2$), 92.0 ($NO_2\underline{C}H$); minor diastereomer observable: 24.3, 26.2, 26.9, 28.3, 28.8, 54.3, 54.9, 69.0, 90.2; ***m/z*** (ESI$^+$) 185 ([M+H]$^+$); **HRMS** (ES$^+$) exact mass calculated for [M+H]$^+$ ($C_9H_{17}N_2O_2^+$) requires *m/z* 185.12845 and 186.13181, found *m/z* 185.12844 and 186.13177.

3.6.9 Preparation and characterisation of (R*,S*)-1-nitrodecahydropyrrolo[1,2-*a*]azocine (5i)

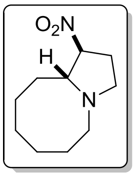

Prepared according to general procedure **F** using **3i** (0.084 g, 0.39 mmol). Purification by FCC to yield the title compound **5i** (0.048 g, 62%, d.r. 67:33)[*] as a yellow oil. **IR** ν_{max}(film)/cm^{-1} 1545 (NO_2), 1373 (NO_2); **^{1}H NMR** (400 MHz, $CDCl_3$) – major diastereomer: δ_H 1.44 - 1.85 (m, 10H), 2.16 – 2.27 (m, 1H, $CH_a\underline{H_b}CHNO_2$), 2.30 - 2.37 (m, 1H, $C\underline{H_a}H_bCHNO_2$), 2.55 (“dt”, *J* = 13.0, 5.0, 1H, $NCH_a\underline{H_b}CH_2CH_2$), 2.80 (ddd, *J* = 11.0, 9.0, 7.0, 1H, $NC\underline{H_a}H_bCH_2CH_2$), 2.87 (“dt”, *J* = 13.5, 6.5, 1H, $CH_a\underline{H_b}CH_2CHNO_2$), 3.14 (m, 2H, $C\underline{H_a}H_bCH_2CHNO_2$, $NC\underline{H}CHNO_2$), 4.57 (ddd, *J* = 8.0, 4.5, 2.5, 1H, $NO_2C\underline{H}$); minor diastereomer observable: 3.23 – 3.34 (m, 1H), 4.92 – 5.00 (m, 1H, $NO_2C\underline{H}$); **^{13}C NMR** (100 MHz, $CDCl_3$) δ_C – major diastereomer: 22.7, 25.8, 26.8, 27.6, 29.3 ($NCH_2\underline{C}H_2CHNO_2$), 33.6, 54.8 ($N\underline{C}H_2CH_2CHNO_2$), 55.5 ($N\underline{C}H_2CH_2CH_2$), 68.2 ($N\underline{C}HCHNO_2$), 91.8 ($\underline{C}HNO_2$); minor diastereomer observable: 23.4, 25.5, 26.9, 27.8, 28.1, 54.3, 65.3 ($N\underline{C}HCHNO_2$), 89.2 ($NO_2\underline{C}H$); ***m/z*** (ESI$^+$) 199 ([M+H]); **HRMS** (ES$^+$) exact mass calculated for $[M+H]^+$ ($C_{10}H_{19}N_2O_2^+$) requires *m/z* 199.14410 and 200.14746, found *m/z* 199.14400 and 200.14736.

3.6.10 Preparation and characterisation of (R*,S*)-11-nitro-1,3,4,6,11,11a-hexahydro-2*H*-pyrido[1,2-*b*]isoquinoline (5j)

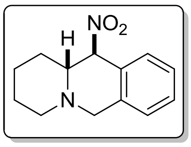

Prepared according to general procedure **F** using **3j** (0.84 g, 0.34 mmol). Purification by FCC to yield the title compound **5j** (0.062 g, 79%, d.r. >98:2) as a yellow oil. **IR** ν_{max}(film)/cm^{-1} 1544 (NO_2), 1351 (NO_2); **^{1}H NMR** (400 MHz, $CDCl_3$) δ_H 1.32 - 1.43 (m,

[*] d.r. represented in the spectra provided in the section below shows a higher d.r.. However, this is due to the minor diastereomer decomposing in the NMR tube.

1H, $NCH_2CH_2CH_a\underline{H_b}$), 1.49 - 1.58 (m, 1H, $NCH(C)CH_a\underline{H_b}$), 1.58 - 1.78 (m, 2H, $NCH_2C\underline{H_a}\underline{H_b}$), 1.80 - 1.88 (m, 1H, $NCH_2CH_2C\underline{H_a}H_b$), 1.95 – 2.02 (m, 1H, $NCH(C)C\underline{H_a}H_b$), 2.30 (t, *J* = 11.5, 1H, $NCH_a\underline{H_b}CH_2CH_2$), 2.88 – 2.93 (m, 1H, $NC\underline{H}CHNO_2$), 3.05 – 3.10 (m, 1H, $NC\underline{H_a}H_bCH_2CH_2$), 3.62 (d, *J* = 15.5, 1H, $NCH_a\underline{H_b}Ar$), 3.81 (d, *J* = 15.5, 1H, $NC\underline{H_a}H_bAr$), 5.65 (d, *J* = 9.0, 1H, $NO_2C\underline{H}$), 7.13 (d, *J* = 7.5, 1H, $Ar\underline{H}$), 7.19 (d, *J* = 7.5, 1H, $Ar\underline{H}$), 7.24 (t, *J* = 7.5, 1H, $Ar\underline{H}$), 7.30 (t, *J* = 7.5, 1H, $Ar\underline{H}$); **^{13}C NMR** (100 MHz, $CDCl_3$) δ_C 23.1 ($NCH_2CH_2\underline{C}H_2$), 25.1 ($NCH_2\underline{C}H_2$), 30.6 ($NCH(C)\underline{C}H_2$), 55.6 ($N\underline{C}H_2CH_2CH_2$), 57.6 ($N\underline{C}H_2Ar$), 62.0 ($N\underline{C}H(C)$), 91.6 ($NO_2\underline{C}H$), 126.0 ($Ar\underline{C}H$), 126.4 ($Ar\underline{C}H$), 127.3 ($Ar\underline{C}H$), 127.8 ($Ar\underline{C}_{quat}$), 129.0 ($Ar\underline{C}H$), 135.1 ($Ar\underline{C}_{quat}$); ***m/z*** ($ESI^+$) 233 ($[M+H]^+$), **HRMS** ($ES^+$) exact mass calculated for $[M+Na]^+$ ($C_{13}H_{16}N_2NaO_2^+$) requires *m/z* 255.1104 and 256.1137 found *m/z* 255.1104 and 256.1138.

3.6.11 Preparation and characterisation of (R*,S*)-12-nitro-5,7,8,9,10,11,11a,12-octahydroazepino[1,2-*b*]isoquinoline (5k)

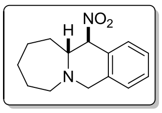

Prepared according to general procedure F using **3k** (0.115 g, 0.440 mmol). Purification by FCC to yield the title compound **5k** (0.087 g, 81%, d.r. >98:2) as a yellow oil. **IR** ν_{max}(film)/cm^{-1} 1547 (NO_2), 1360 (NO_2); **^{1}H NMR** (400 MHz, $CDCl_3$) δ_H 1.45 - 1.77 (m, 8H), 2.80 – 2.95 (m, 2H, $NC\underline{H_2}CH_2$), 3.44 – 3.51 (m, 1H, $NC\underline{H}CHNO_2$), 3.84 (d, *J* = 15.5, 1H, $NC\underline{H_a}H_bAr$), 3.92 (d, *J* = 15.5, 1H, $NCH_a\underline{H_b}Ar$), 5.50 (d, *J* = 7.0, 1H, $NO_2C\underline{H}$), 7.06 (d, *J* = 7.5, 1H, $Ar\underline{H}$), 7.11 – 7.30 (m, 3H, $Ar\underline{H}$); **^{13}C NMR** (100 MHz, $CDCl_3$) δ_C 25.0, 28.1, 28.3, 29.4, 54.9 ($N\underline{C}H_2$), 55.1 ($N\underline{C}H_2$), 63.0 ($NO_2CH\underline{C}H$), 89.9 ($NO_2\underline{C}H$), 126.5 ($Ar\underline{C}H$), 126.9 ($Ar\underline{C}H$), 128.1 ($Ar\underline{C}H$), 128.1 ($Ar\underline{C}_{quat}$), 128.9 ($Ar\underline{C}H$), 136.7

(Ar$\underline{C}_{quat}$); ***m/z*** (ESI$^+$) 247 ([M+H]$^+$); **HRMS** (ES$^+$) exact mass calculated for [M+H]$^+$ ($C_{14}H_{19}N_2O_2^+$) requires *m/z* 247.1441 and 248.1474, found *m/z* 247.1443 and 248.1472.

3.6.12 Preparation and characterisation of (R*,S*)-13-nitro-7,8,9,10,11,12,12a,13-octahydro-5*H*-azocino[1,2-*b*]isoquinoline (5l)

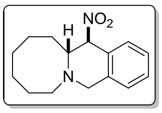

Prepared according to general procedure **F** using **3l** (0.100 g, 0.36 mmol). Purification by FCC to yield the title compound **5l** (0.049 g, 52%, d.r. >96:4) as a yellow oil. **IR** ν_{max}(film)/cm^{-1} 1544 (NO_2), 1363 (NO_2); **^{1}H NMR** (500 MHz, C_6D_6) δ_H 1.16 - 1.59 (m, 10H), 2.36 – 2.50 (m, 2H, NC$\underline{H_2}$CH$_2$), 3.44 (d, *J* = 16.0, 1H, NCHCH$_a\underline{H_b}$Ar), 3.66 (d, *J* = 16.0, 1H, NCHC$\underline{H_a}$H$_b$Ar), 3.71 (ddd, *J* = 9.5, 6.5, 3.5, 1H, NC$\underline{H}$CH$_2$Ar), 5.19 (d, *J* = 6.5, 1H, NO$_2$C$\underline{H}$), 6.74 (d, *J* = 7.5, 1H, Ar$\underline{H}$), 6.92 (t, *J* = 7.5, 1H, Ar$\underline{H}$), 6.98 – 7.03 (m, 2H, ArH); **^{13}C NMR** (125 MHz, $CDCl_3$) δ_C 26.6, 26.7, 27.1, 27.2, 28.9, 51.7 (N$\underline{C}$H$_2$CH$_2$), 53.2 (N$\underline{C}$H$_2$Ar), 61.0 (NC$\underline{H}$CHNO$_2$), 89.1 (NO$_2\underline{C}$H), 126.6 (Ar$\underline{C}$H), 126.8 (Ar$\underline{C}$H), 128.2 (Ar$\underline{C}_{quat}$), 128.7 (Ar$\underline{C}$H), 129.1 (Ar$\underline{C}$H), 136.4 (Ar$\underline{C}_{quat}$); ***m/z*** (ESI$^+$) 261 ([M+H]$^+$); **HRMS** (ESI$^+$) exact mass calculated for [M+H]$^+$ ($C_{15}H_{21}N_2O_2^+$) requires *m/z* 261.15975 and 262.16311, found *m/z* 261.15953 and 262.16293.

3.7 Preparation and characterisation of 1-(4-nitrobutyl)-2,3,4,5-tetrahydro-1*H*-azepine (7)

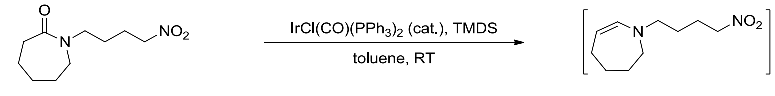

To a solution of **3a** (1.0 eq., 0.023 mmol, 0.0050 g) in toluene-d$_8$ (0.04 M) at room temperature was added TMDS (2.0 eq., 0.047 mmol, 0.0080 mL) and IrCl(CO)(PPh$_3$)$_2$ (0.05 eq., 1.2 µmol, 0.0010 g) in an NMR tube. After approximately 5 min the reaction

was observed to be at full conversion to the title compound **7** by NMR analysis. **^{1}H NMR** (500 MHz, toluene-d$_8$) δ_H 0.89 - 1.03 (m, 2H), 1.29 - 1.52 (m, 8H), 2.37 (t, *J* = 7.0, 2H, $C\underline{H_2}(CH_2)_3NO_2$), 2.48 - 2.60 (m, 2H, $NCHCH(CH_2)_3C\underline{H_2}$), 3.49 (t, *J* = 7.0, 2H, $C\underline{H_2}NO_2$), 4.39 - 4.49 (m, 1H, $NCHC\underline{H}$), 5.58 (d, *J* = 9.5, 1H, $NC\underline{H}$); **^{13}C NMR** (125 MHz, toluene-d$_8$) δ_C 25.2, 26.0, 28.5, 29.1, 31.5, 54.9 ($NCHCH(CH_2)_3\underline{C}H_2$), 57.7 ($\underline{C}H_2(CH_2)_3NO_2$), 75.2 ($\underline{C}H_2NO_2$), 103.0 ($NCH\underline{C}H$), 140.2 ($N\underline{C}H$).

3.8 Preparation and characterisation of 1-(4-nitrobutyl)-3,4,5,6-tetrahydro-2*H*-azepinium chloride (8)

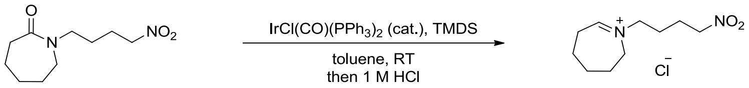

To a solution of **3a** (1.0 eq., 0.467 mmol, 0.100 g) in toluene (0.01 M) at room temperature under an inert atmosphere of argon was added TMDS (2.0 eq., 0.935 mmol, 0.165 mL) and $IrCl(CO)(PPh_3)_2$ (0.005 eq., 0.002 mmol, 0.002 g). After 5 min the reaction was quenched with 1 M HCl (6 mL). The aqueous layer was separated and the organic layer was extracted (1 M HCl, 3 × 6 mL). The combined aqueous extracts were washed (Et_2O, 3 × 15 mL) and concentrated *in vacuo* to yield the title compound **8** (~100%) as a green oil. **IR** ν_{max}(film)/cm^{-1} 1680 (C=N), 1545 (NO_2), 1377 (NO_2); **^{1}H NMR** (400 MHz, D_2O) δ_H 1.41 - 1.60 (m, 4H, $NCH_2C\underline{H_2}$, $NCHCH_2C\underline{H_2}$), 1.62 - 1.90 (m, 6H, $N(CH_2)_2C\underline{H_2}$, $C\underline{H_2}CH_2NO_2$, $C\underline{H_2}(CH_2)_2NO_2$), 2.68 (“d”, *J* = 4.5, 2H, $NCHC\underline{H_2}$), 3.72 - 3.90 (m, 4H, $C\underline{H_2}(CH_2)_3NO_2$, $NC\underline{H_2}$), 4.38 (t, *J* = 6.5, 2H, $C\underline{H_2}NO_2$), 8.57 (t, *J* = 5.5, 1H, $NC\underline{H}$); **^{13}C NMR** (100 MHz, D_2O) δ_C 20.7, 23.4, 23.7, 24.1, 29.2, 30.8 ($NCH\underline{C}H_2$), 55.9 ($N\underline{C}H_2$), 63.3 ($\underline{C}H_2(CH_2)_3NO_2$), 74.9 ($\underline{C}H_2NO_2$), 185.1 ($N\underline{C}H$); ***m/z*** (ESI$^+$) 199 ([M]$^+$) **HRMS** (ES$^+$) exact mass calculated for [M]$^+$ ($C_{10}H_{19}N_2O_2^+$) requires *m/z* 199.1441, found *m/z* 199.1437.

3.9 Preparation and characterisation of *N*-(octahydro-2*H*-quinolizin-1-yl)acetamide ((±)-*epi*-epiquinamide)

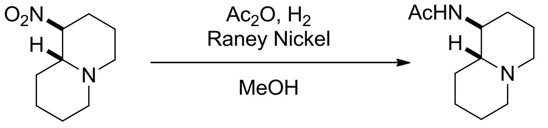

(±)-epi-epiquinamide

To **3a** (90 mg, 0.49 mmol) dissolved in methanol (5 ml) was added acetic anhydride (230 µl, 2.45 mmol) followed by an Raney 2400 nickel slurry in water (420 µl) under nitrogen. The atmosphere was then exchanged for hydrogen (purging 3 times) and the reaction was stirred for 40 h. The reaction atmosphere was changed back to nitrogen the reaction mixture was filtered through celite, washing with methanol. Concentration of the filtrate *in vacuo* afforded crude (±)-*epi*-epiquinamide, which was purified by FCC (20-50% MeOH in EtOAc) giving the title compound (±)-*epi*-epiquinamide (72 mg, 75%, d.r. >98:2) as a white solid (m.p. 172-174 °C), literature m.p. 170-175 ºC.[3] **^{1}H NMR** (400 MHz, $CDCl_3$) δ_H 1.06 - 1.35 (m, 3H), 1.47 - 1.88 (m, 7H), 1.94 - 2.06 (m, 6H), 2.78 (d, *J* = 11.0, 1H, $NC\underline{H}_aH_b$), 2.87 (d, *J* = 11.5, 1H, $NC\underline{H}_cH_d$), 3.64 - 3.84 (m, 1H, C<u>H</u>NHAc), 5.13 (br. s., 1H, N<u>H</u>Ac); **^{13}C NMR** (100 MHz, $CDCl_3$) δ_C 23.4 (Ac), 23.9, 24.3, 25.5, 29.0, 32.0, 51.0 (<u>C</u>HNHAc), 55.7 ($N\underline{C}H_2$), 56.4 ($N\underline{C}H_2$), 67.3 (N<u>C</u>H), 169.4 (<u>C</u>O). ***m/z*** (ESI^+) 197 ($[M+H]^+$); Data in agreement with that reported previously in the literature.[3]

4. ^{1}HNMR and ^{13}CNMR Spectra

4.1 Nitroalkane Spectra

4.1.1 ^{1}HNMR spectrum of 1-(4-Nitrobutyl)pyrrolidin-2-one (3b)

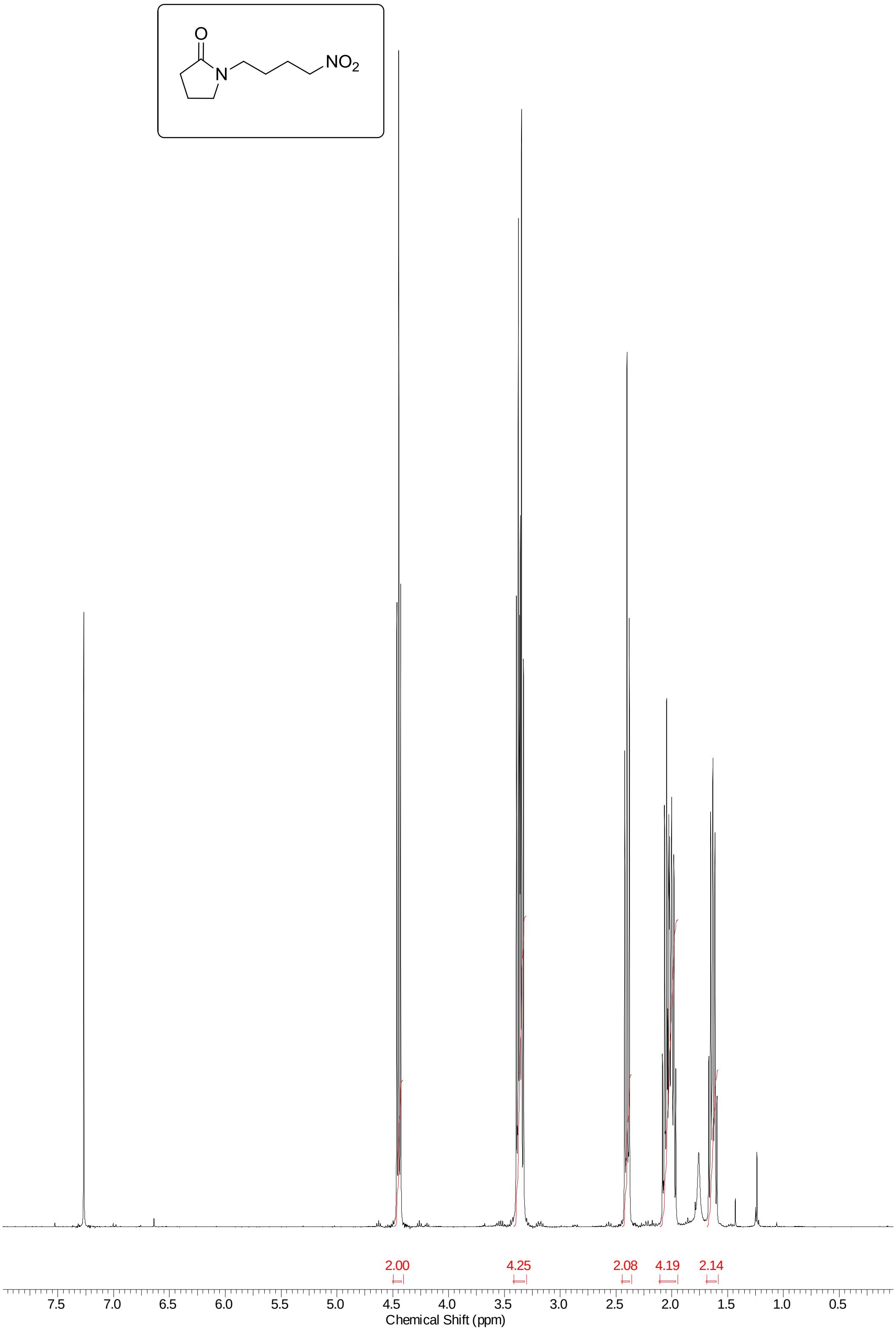

4.1.2 ^{13}CNMR spectrum of 1-(4-Nitrobutyl)pyrrolidin-2-one (3b)

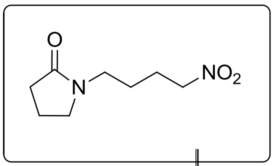

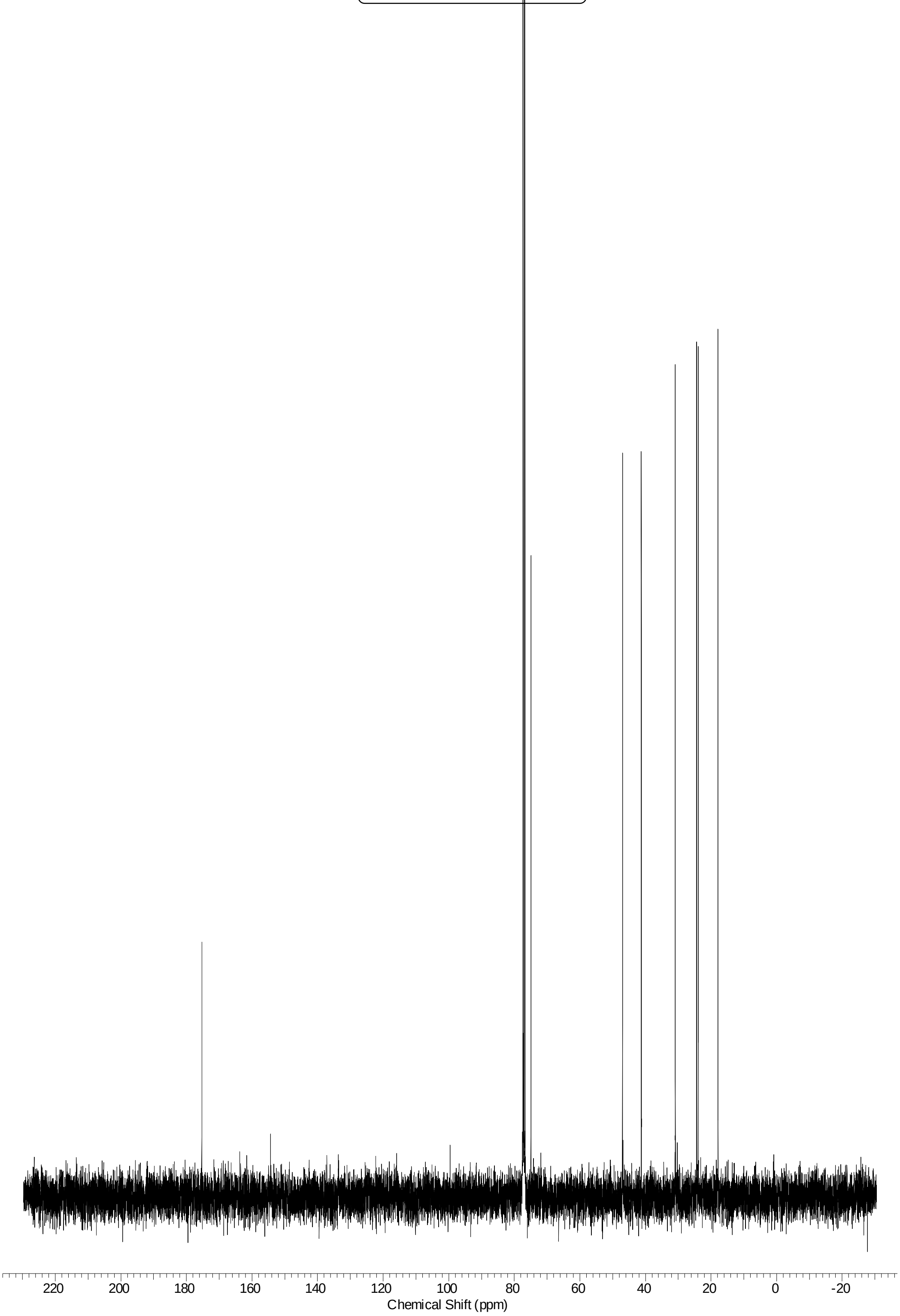

4.1.3 ^{1}HNMR spectrum of 1-(4-Nitrobutyl)piperidin-2-one (3c)

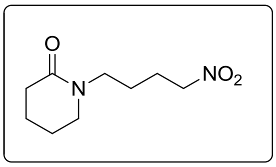

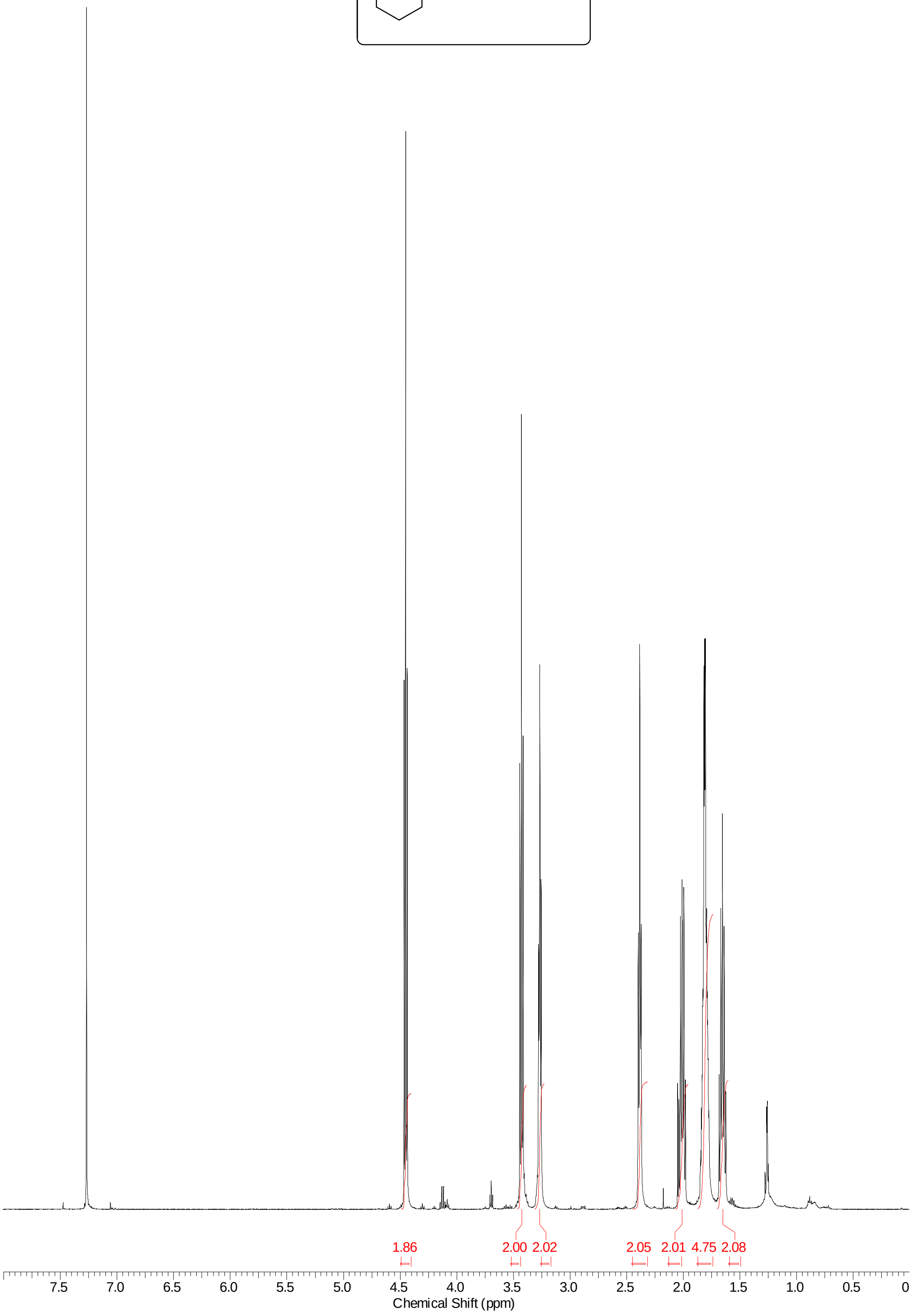

4.1.4 ^{13}CNMR spectrum of 1-(4-Nitrobutyl)piperidin-2-one (3c)

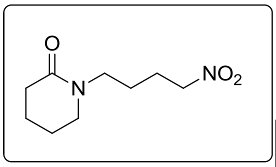

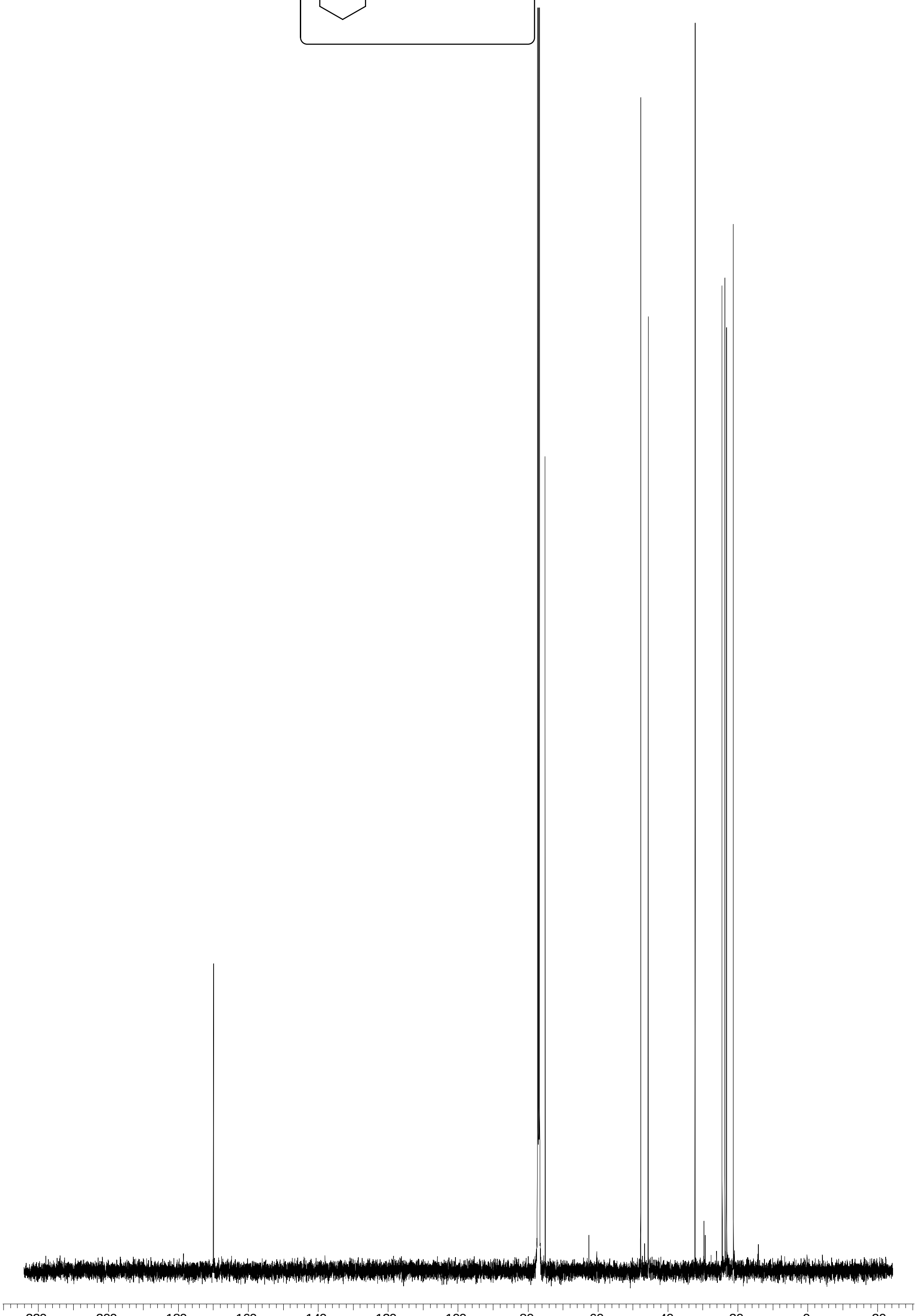

4.1.5 ^{1}HNMR spectrum of 1-(4-Nitrobutyl)azepan-2-one (3a)

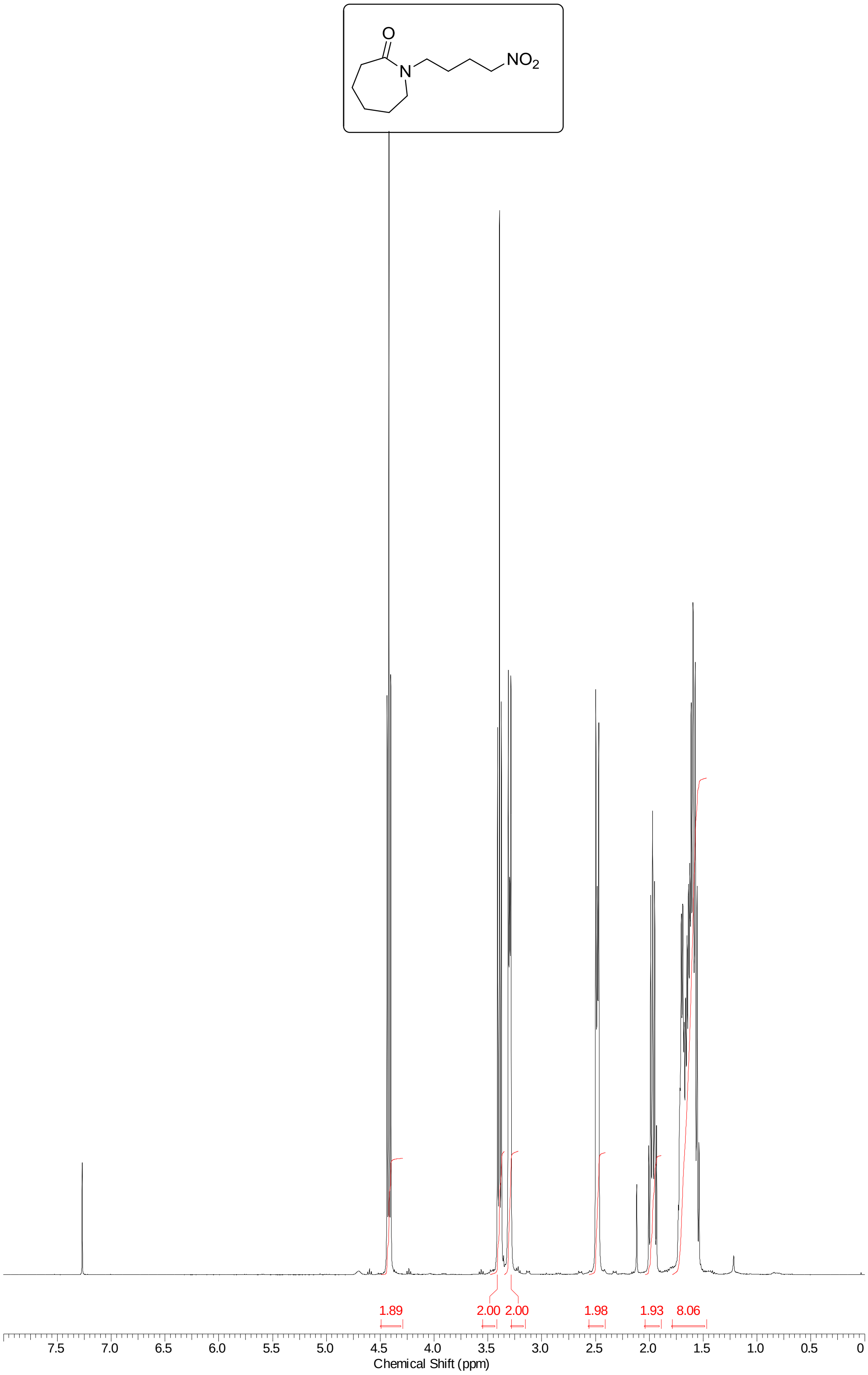

4.1.6 ^{13}CNMR spectrum of 1-(4-Nitrobutyl)azepan-2-one (3a)

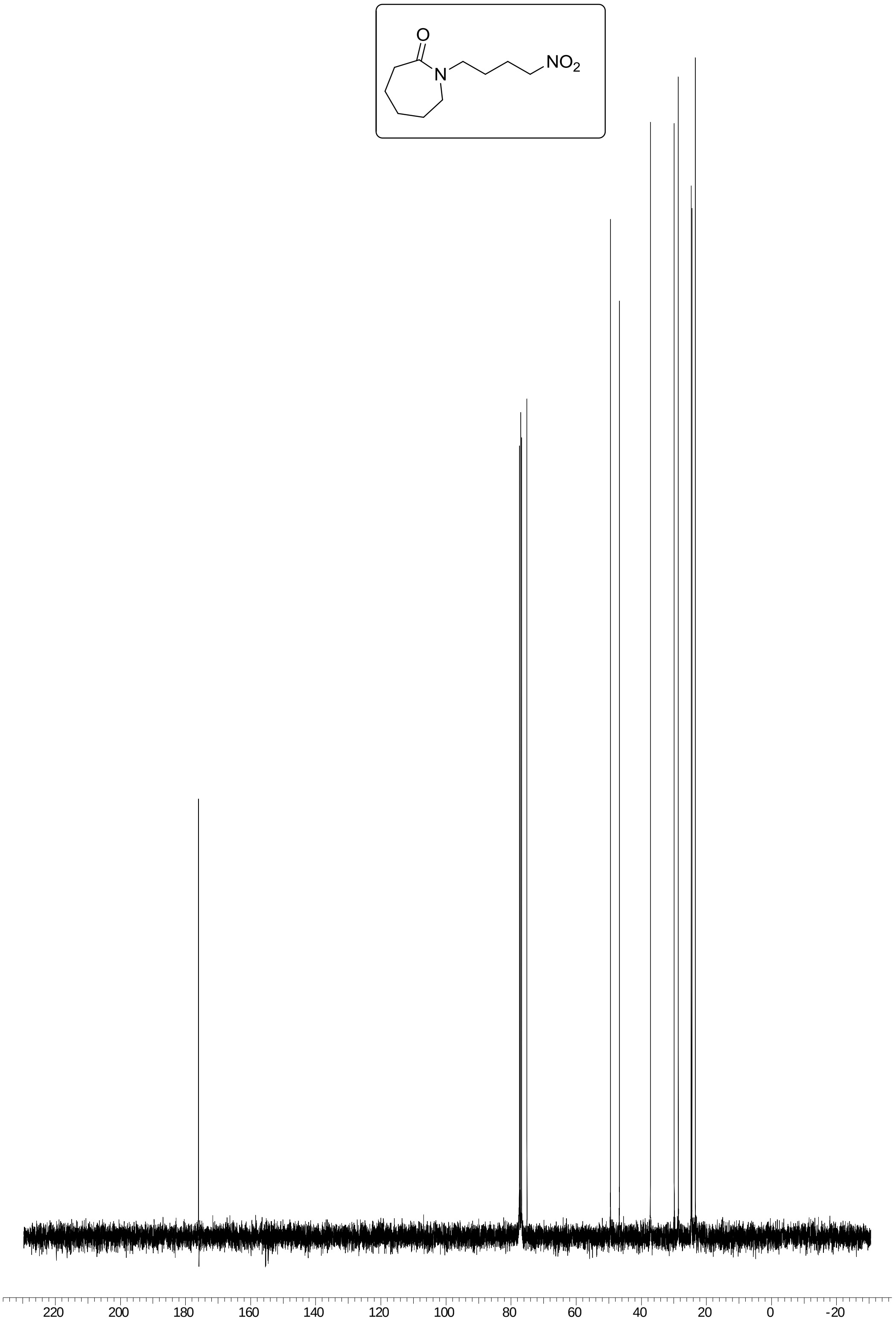

4.1.7 ^{1}HNMR spectrum of 1-(4-Nitrobutyl)azocan-2-one (3d)

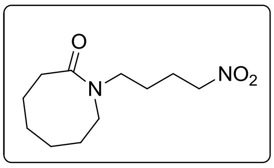

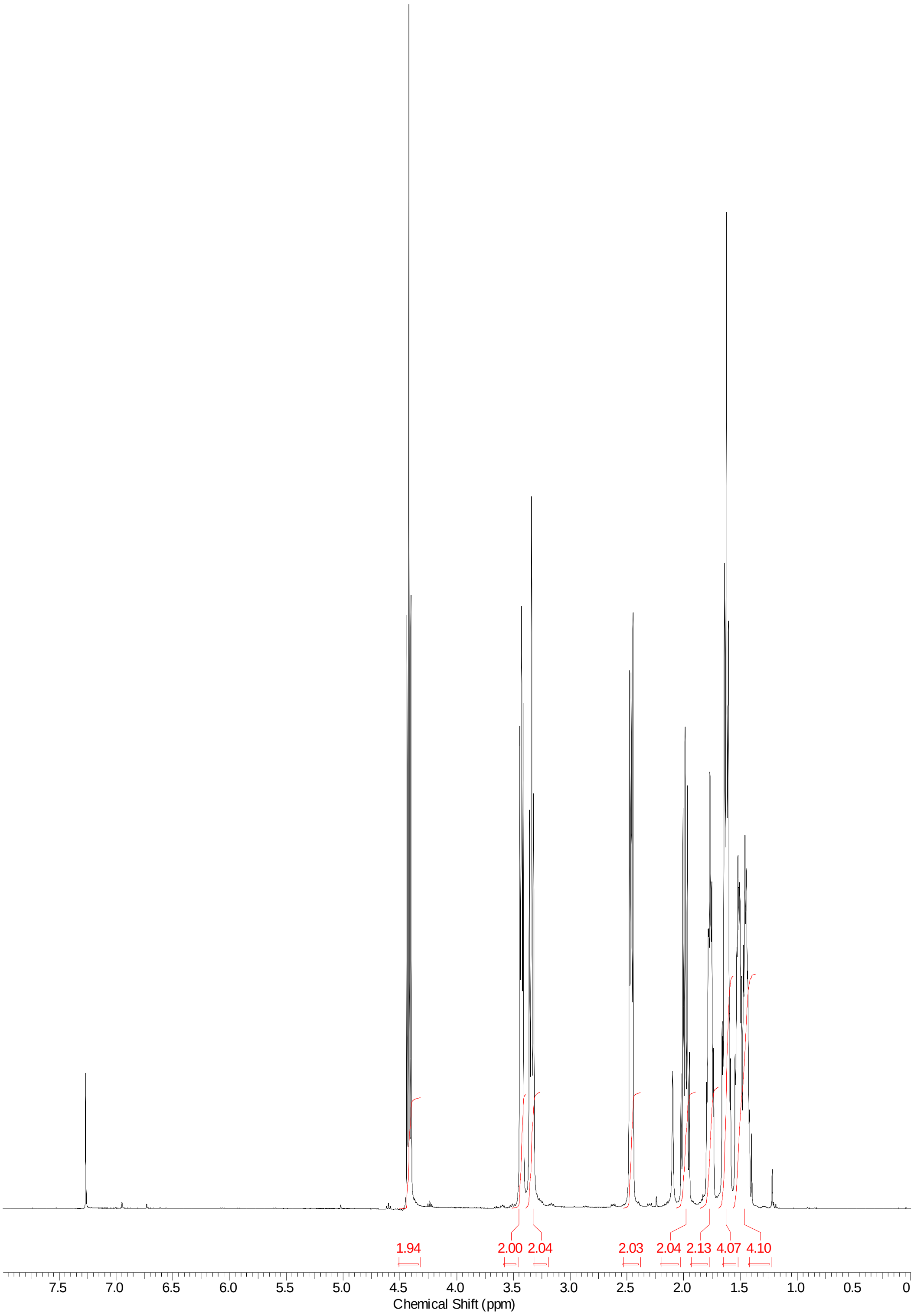

4.1.8 ^{13}CNMR spectrum of 1-(4-Nitrobutyl)azocan-2-one (3d)

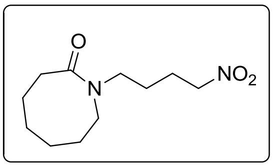

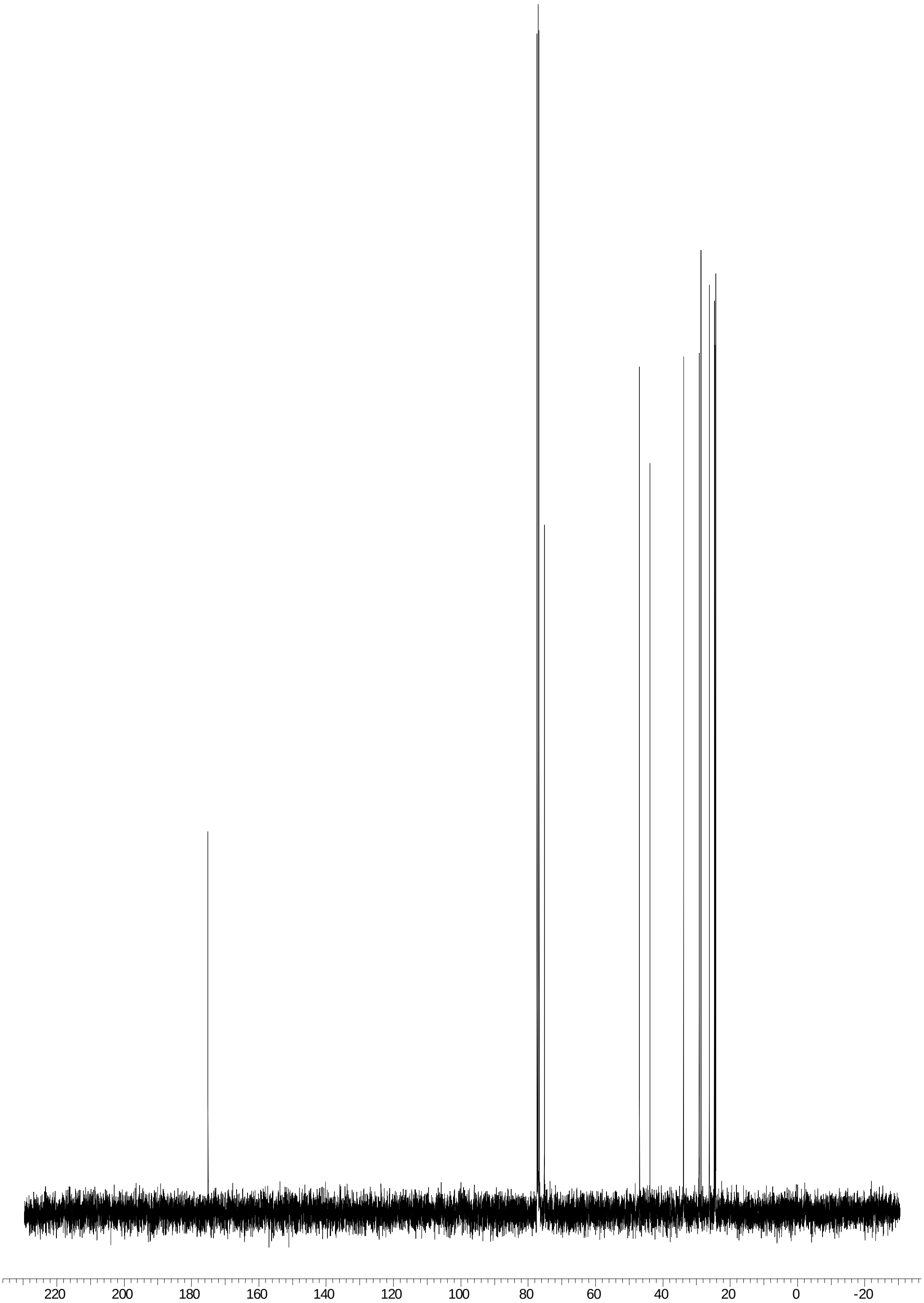

4.1.9 ^{1}HNMR spectrum of 4-(4-nitrobutyl)-1,4-oxazepan-5-one (3e)

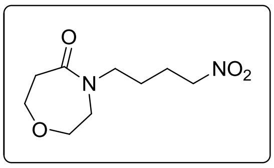

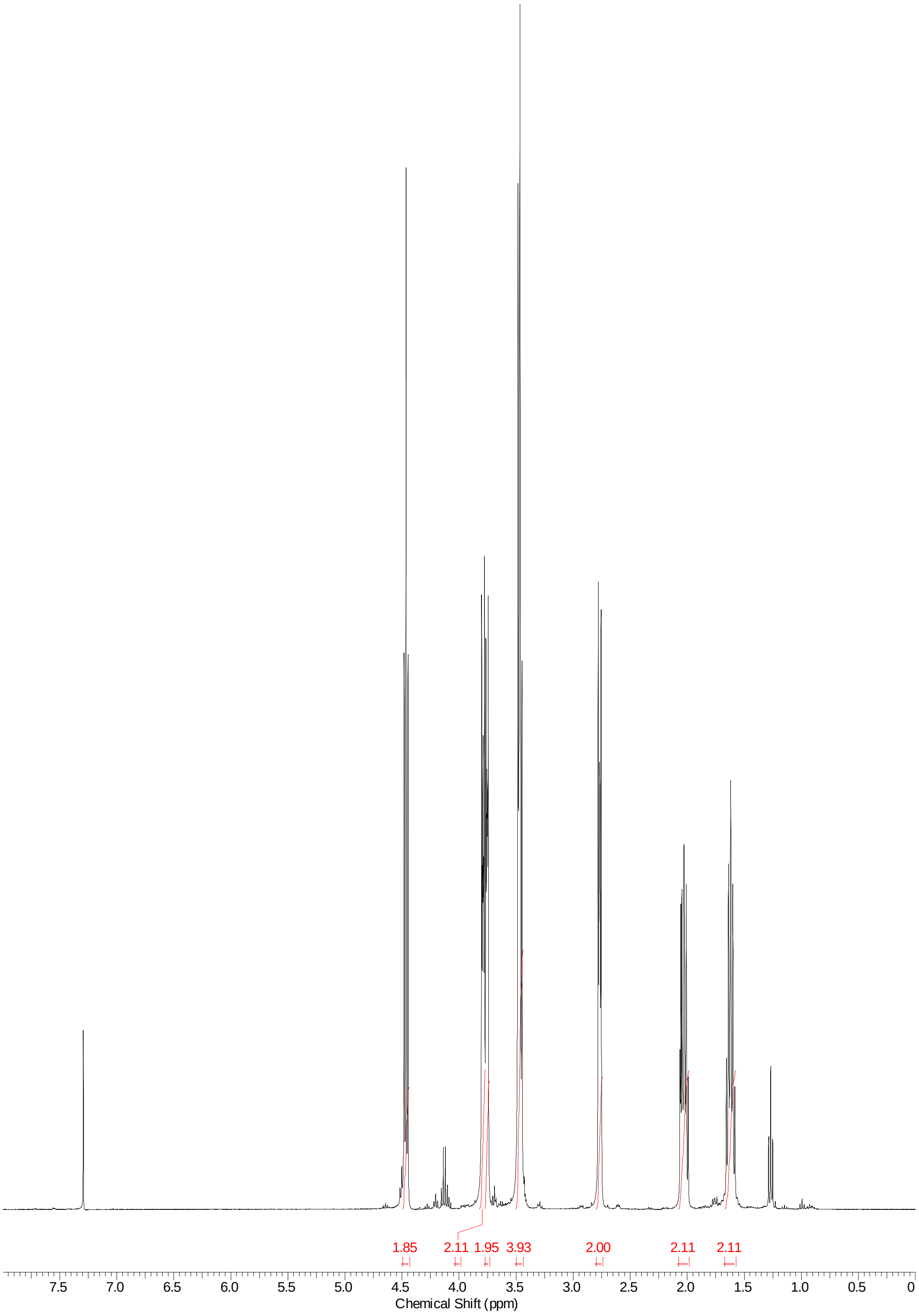

4.1.10 ^{13}CNMR spectrum of 4-(4-nitrobutyl)-1,4-oxazepan-5-one (3e)

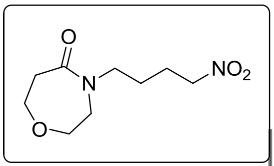

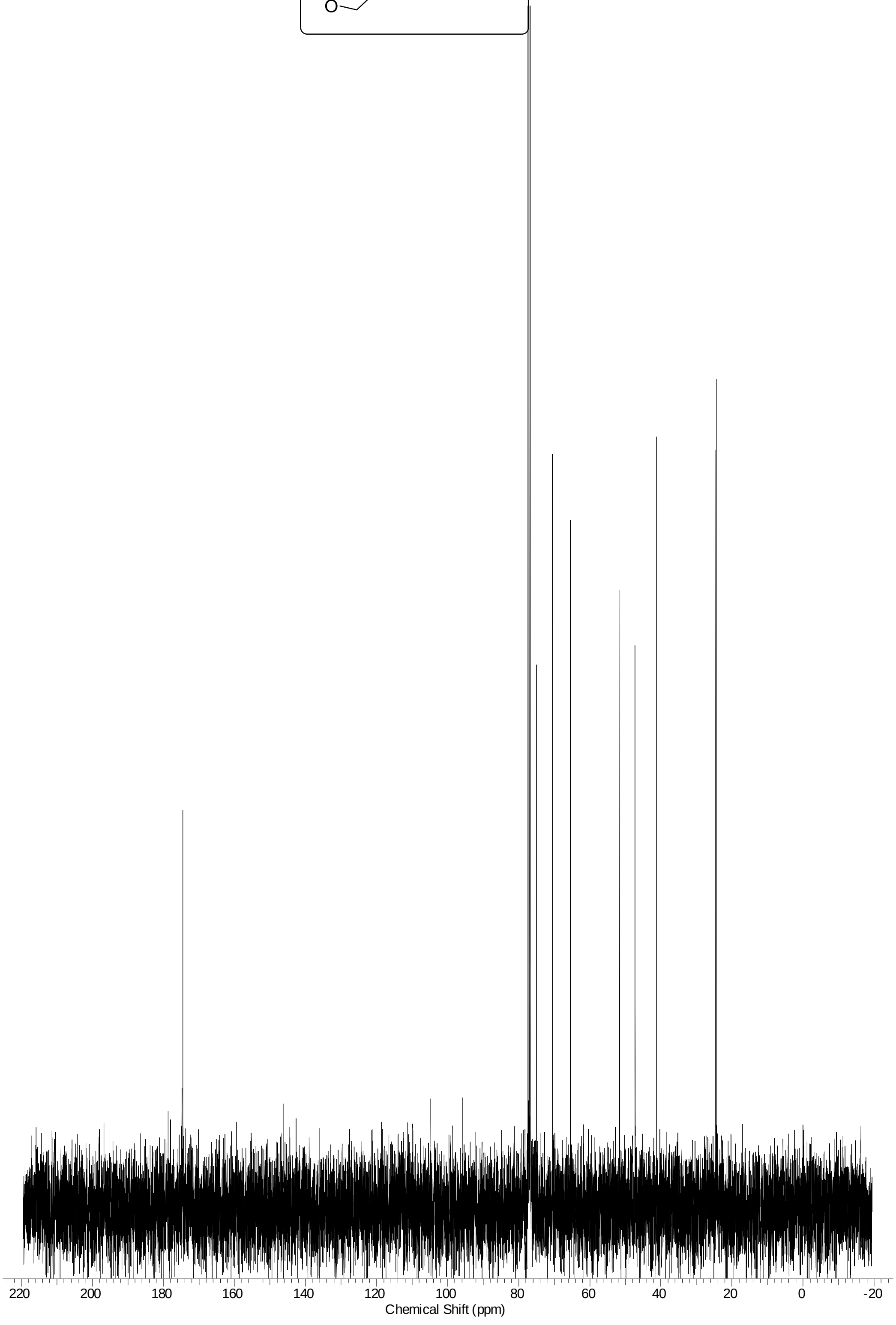

4.1.11 ^{1}HNMR spectrum of 1-(5-Nitropentyl)azepan-2-one (3f)

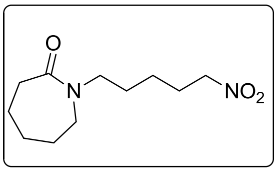

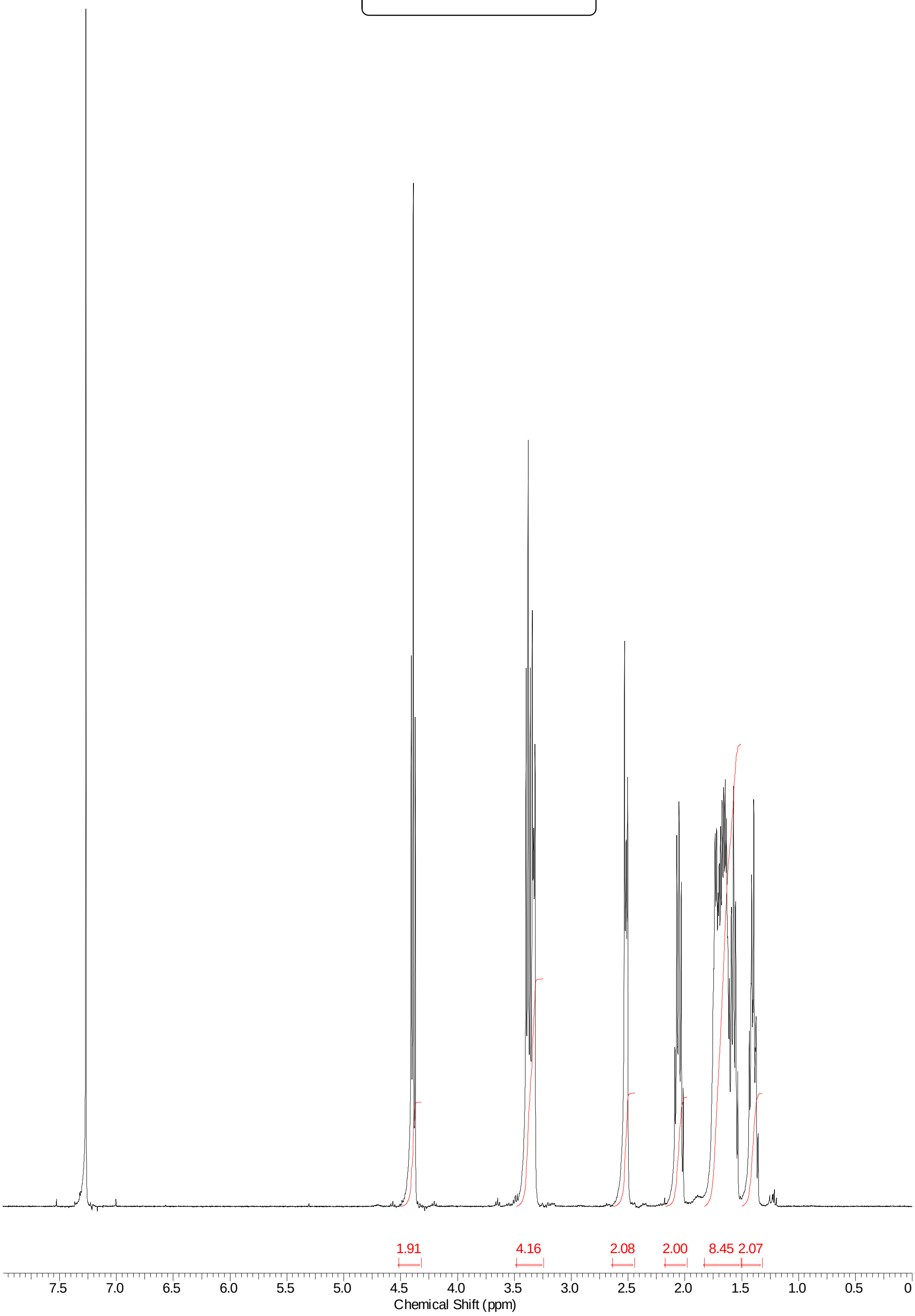

4.1.12 ^{13}CNMR spectrum of 1-(5-Nitropentyl)azepan-2-one (3f)

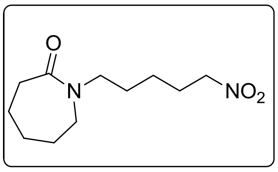

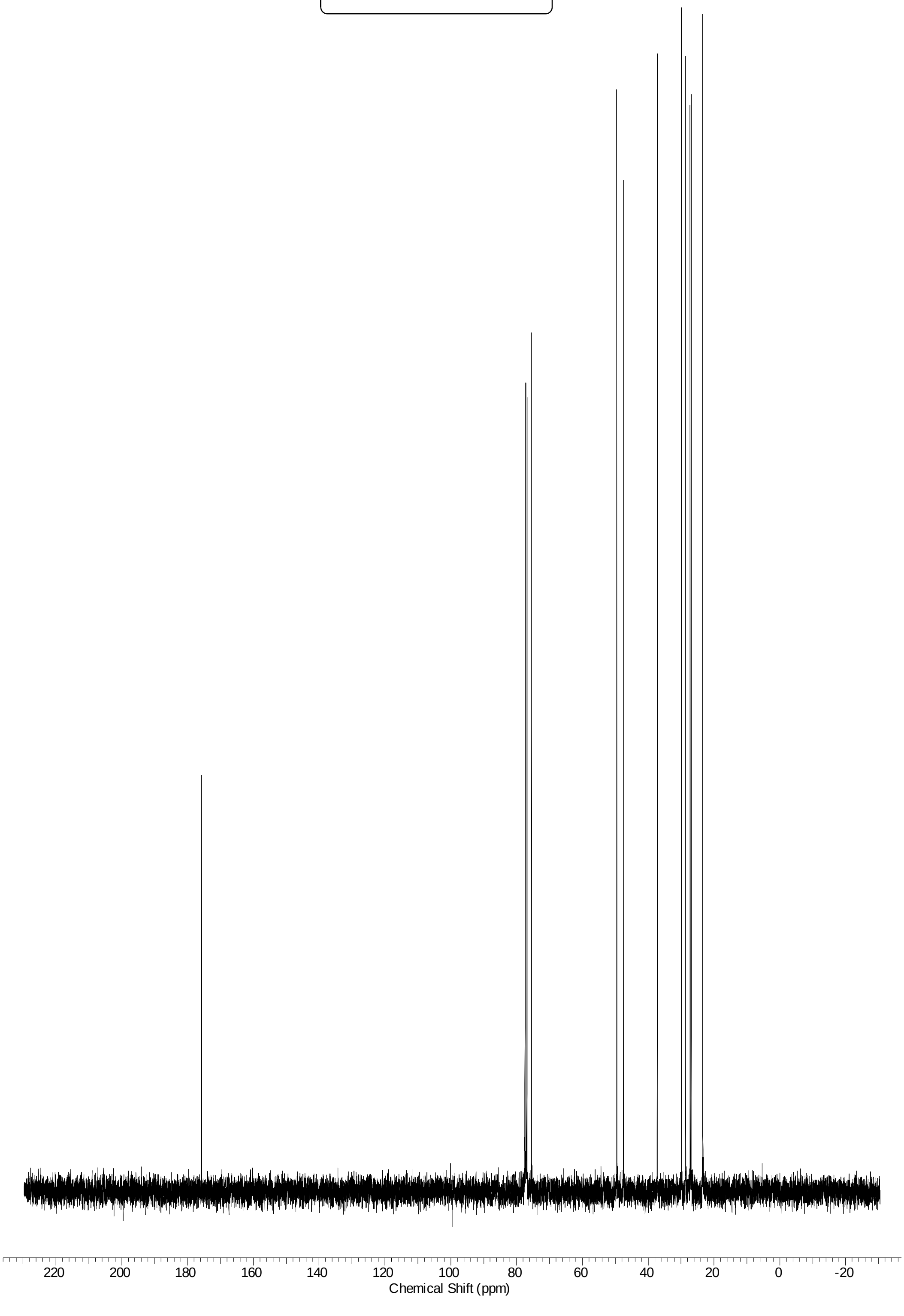

4.1.13 ^{1}HNMR spectrum of 1-(5-Nitropentyl)pyrrolidin-2-one (3g)

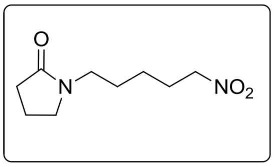

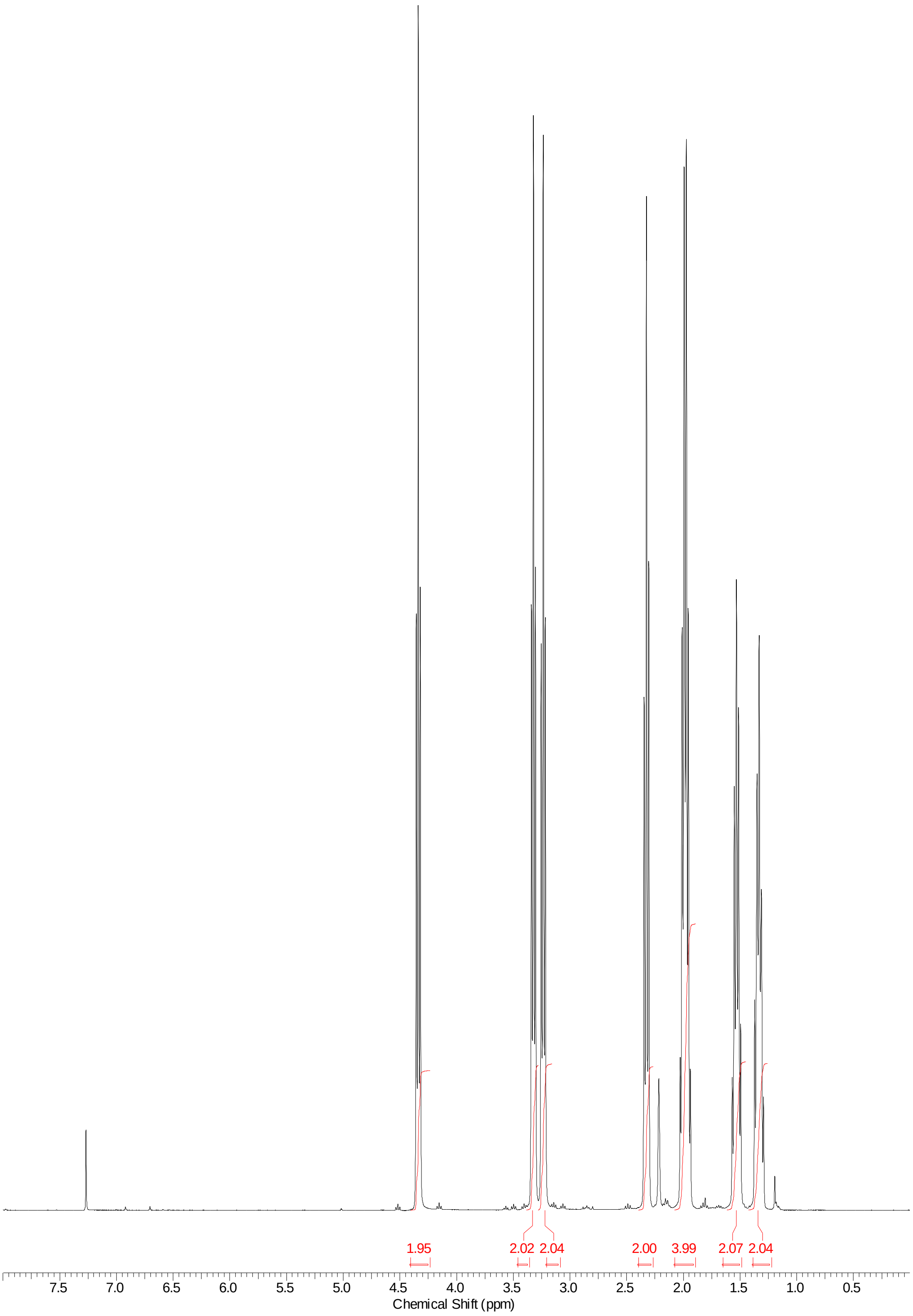

4.1.14 ^{13}CNMR spectrum of 1-(5-Nitropentyl)pyrrolidin-2-one (3g)

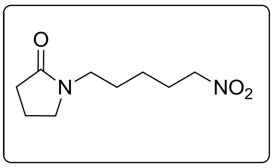

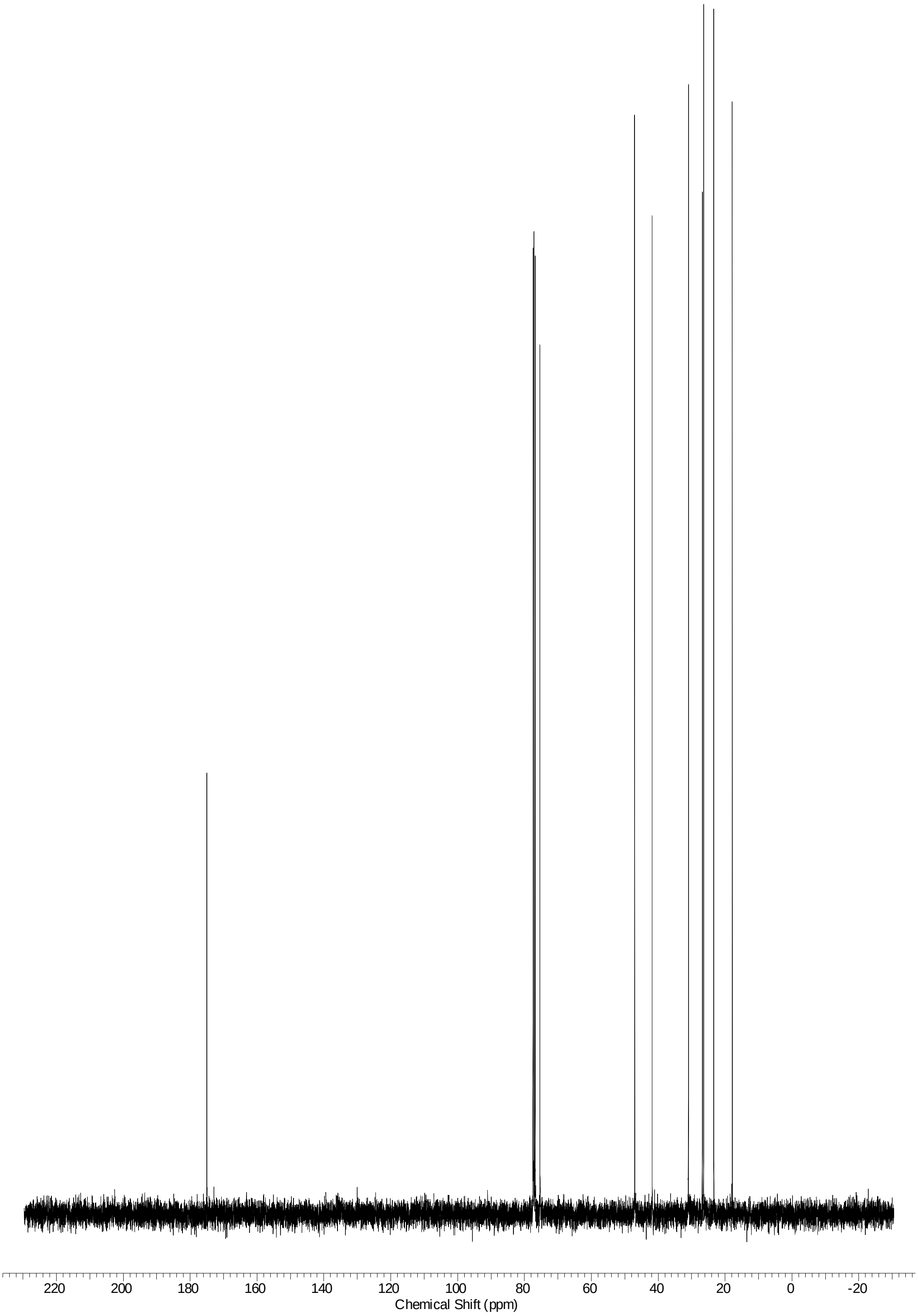

4.1.15 ^{1}HNMR spectrum of 1-(3-nitropropyl)azepan-2-one (3h)

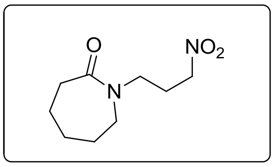

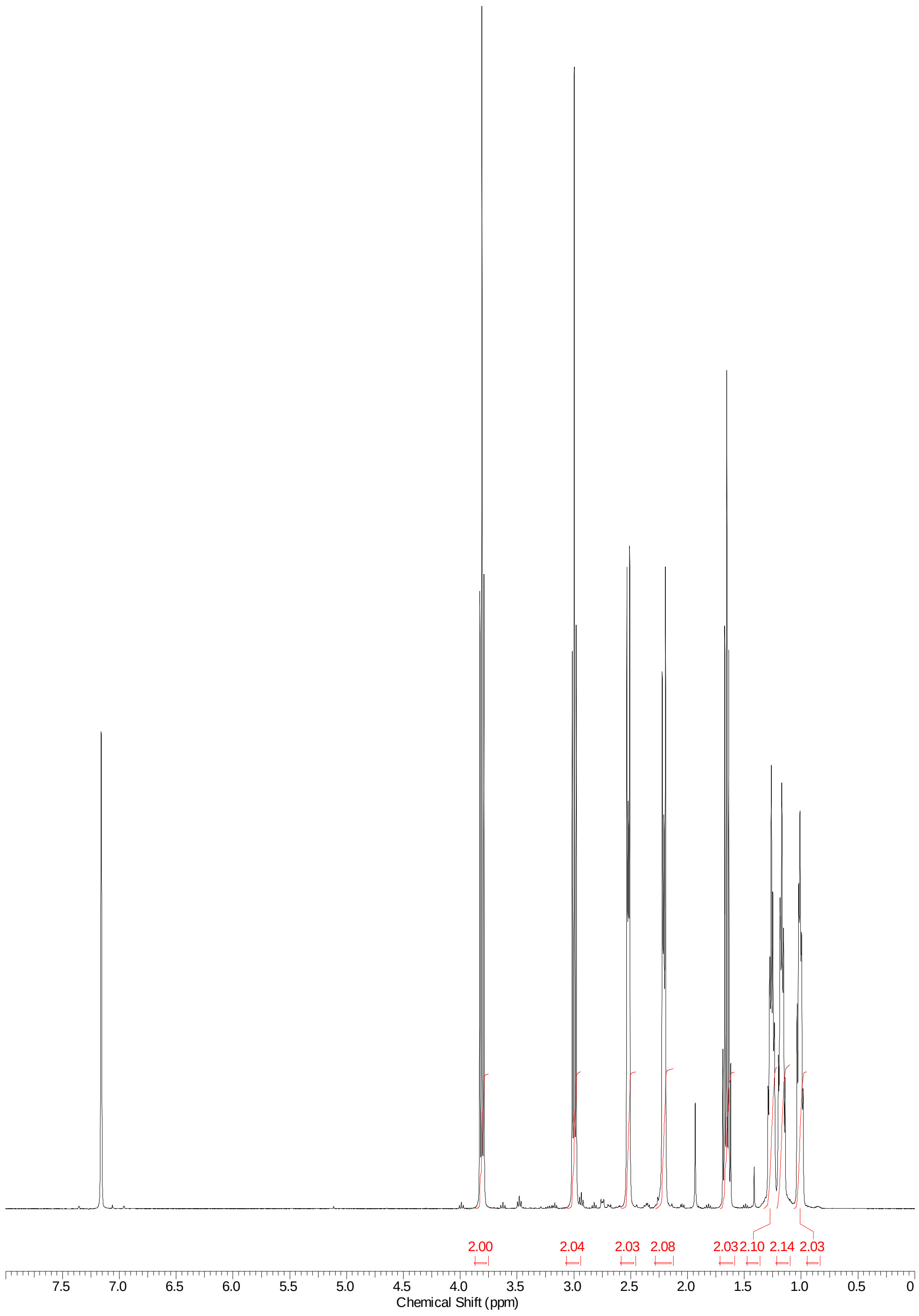

4.1.16 ^{13}CNMR spectrum of 1-(3-nitropropyl)azepan-2-one (3h)

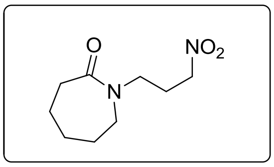

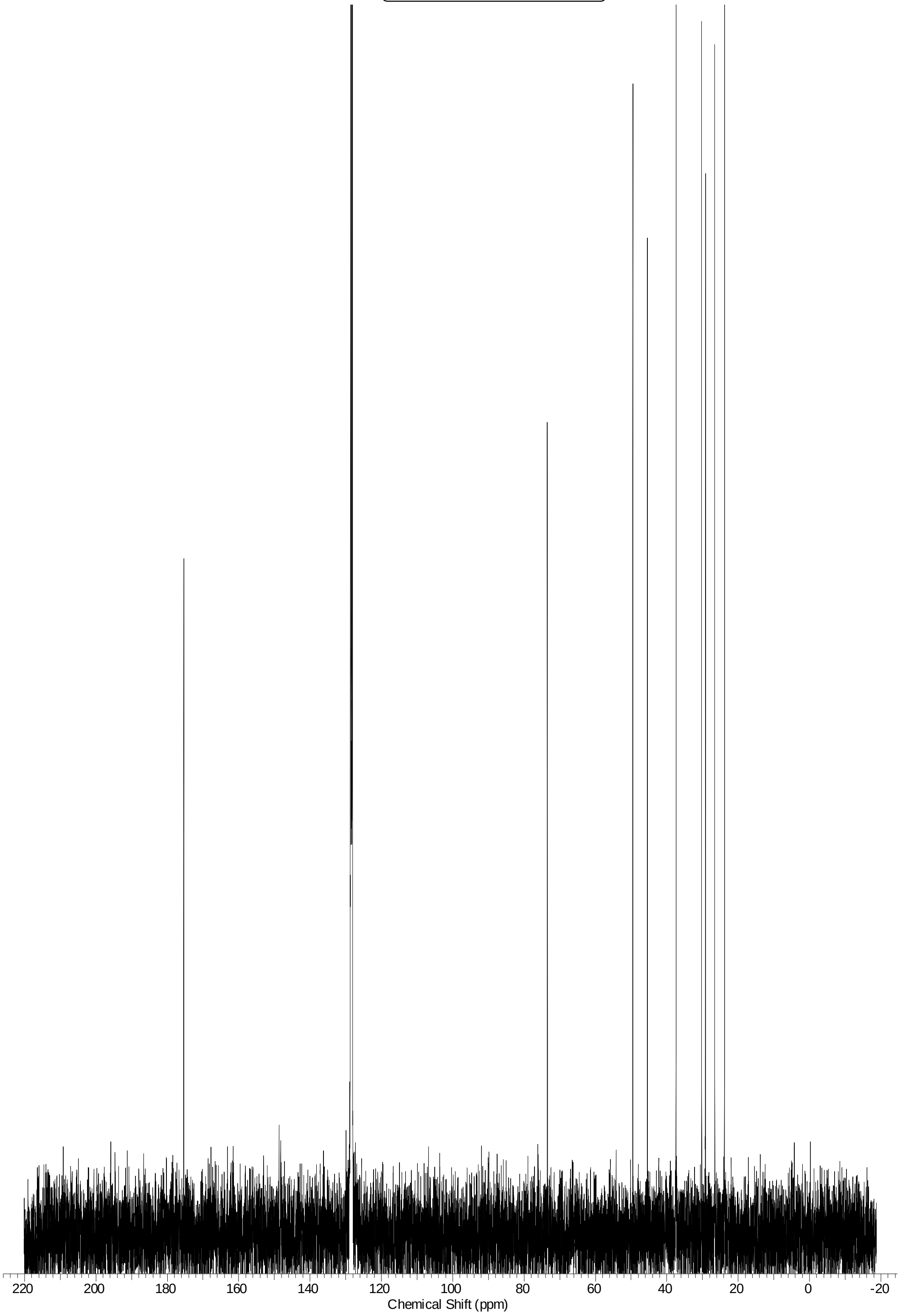

4.1.17 ^{1}HNMR spectrum of 1-(3-nitropropyl)azocan-2-one (3i)

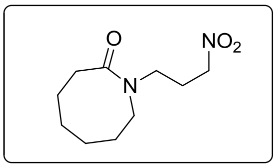

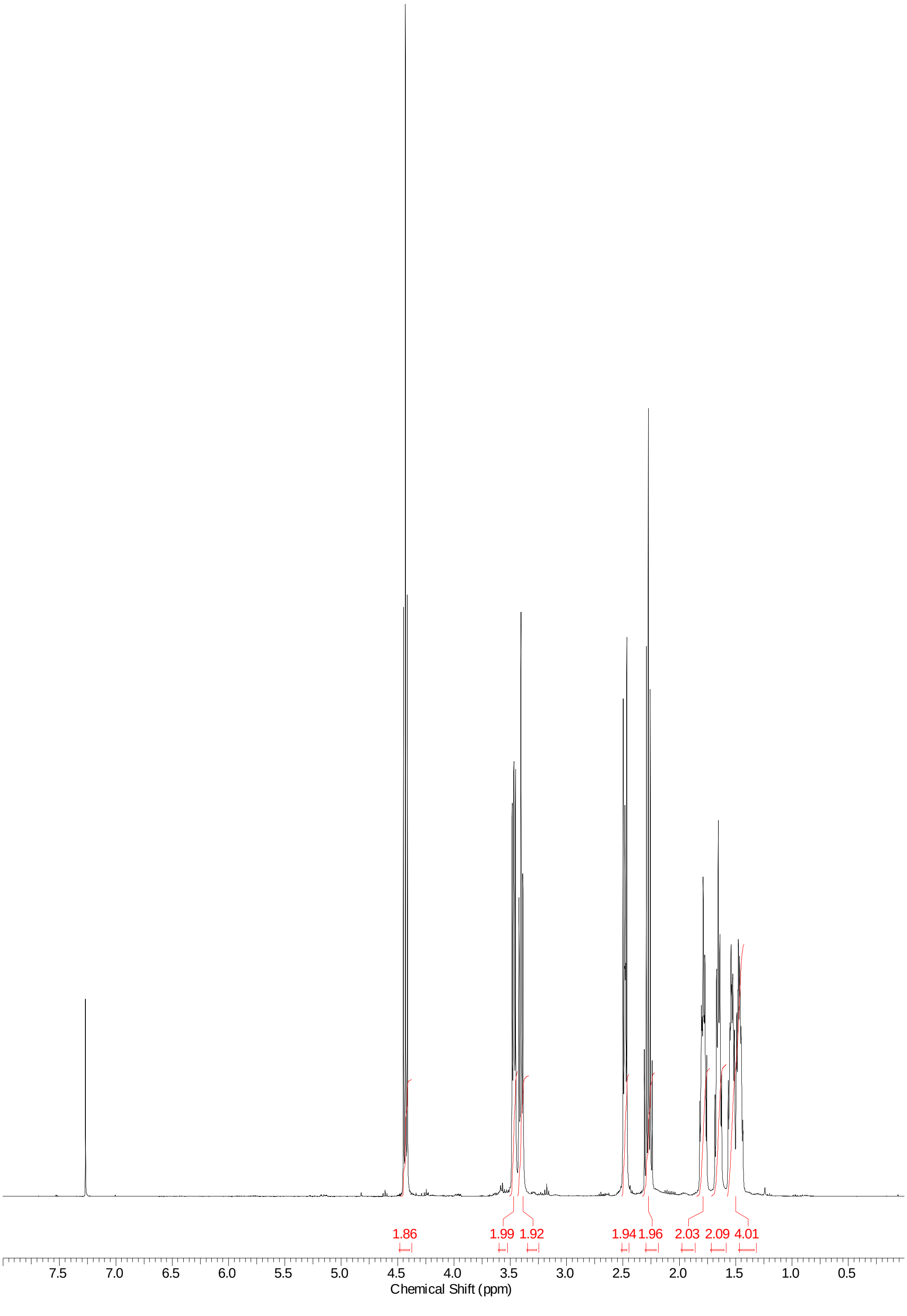

4.1.18 ^{13}CNMR spectrum of 1-(3-nitropropyl)azocan-2-one (3i)

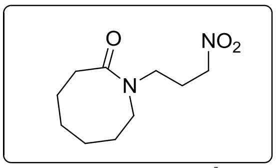

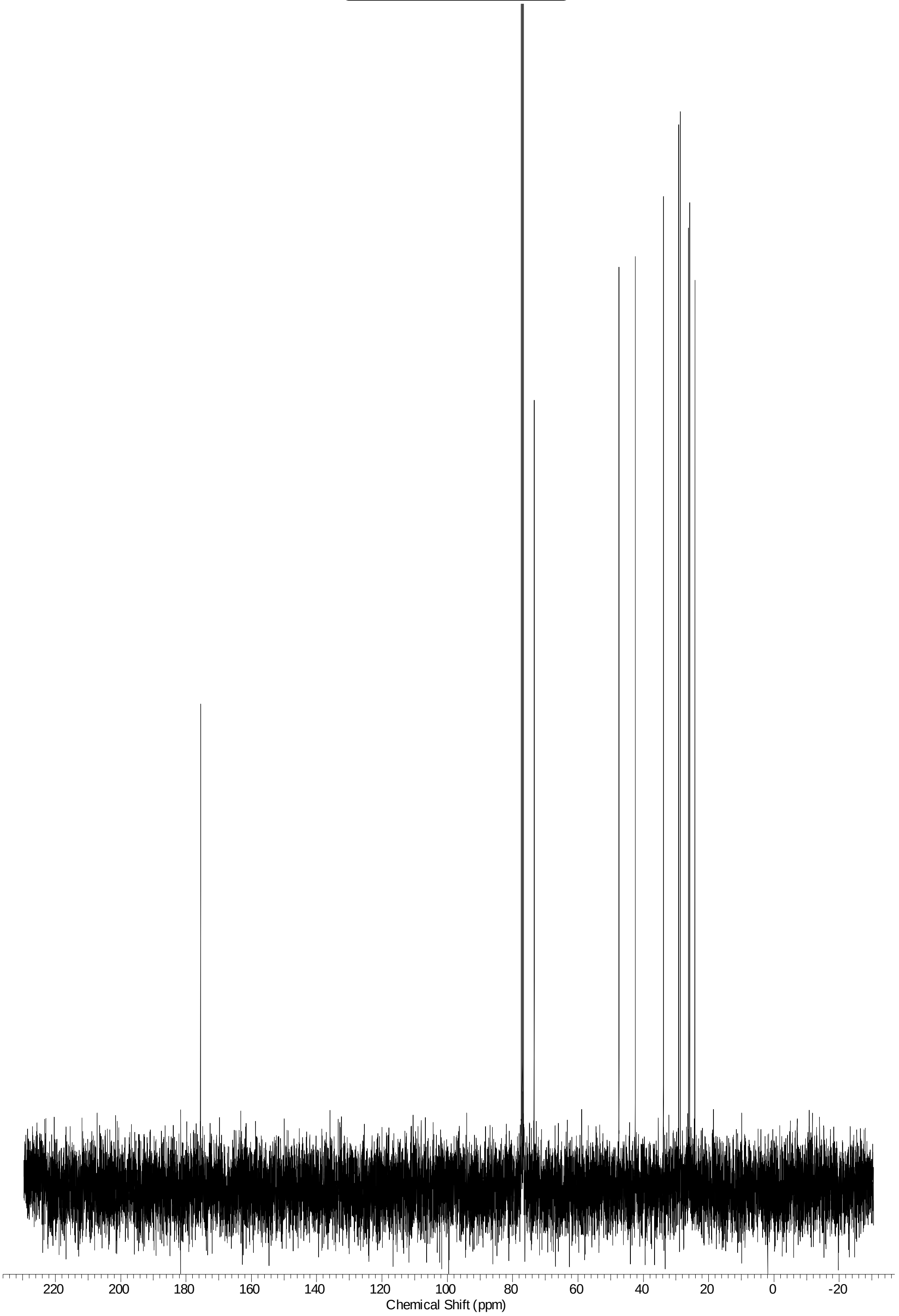

4.1.19 ^{1}HNMR spectrum of 1-[2-(nitromethyl)benzyl]piperidin-2-one (3j)

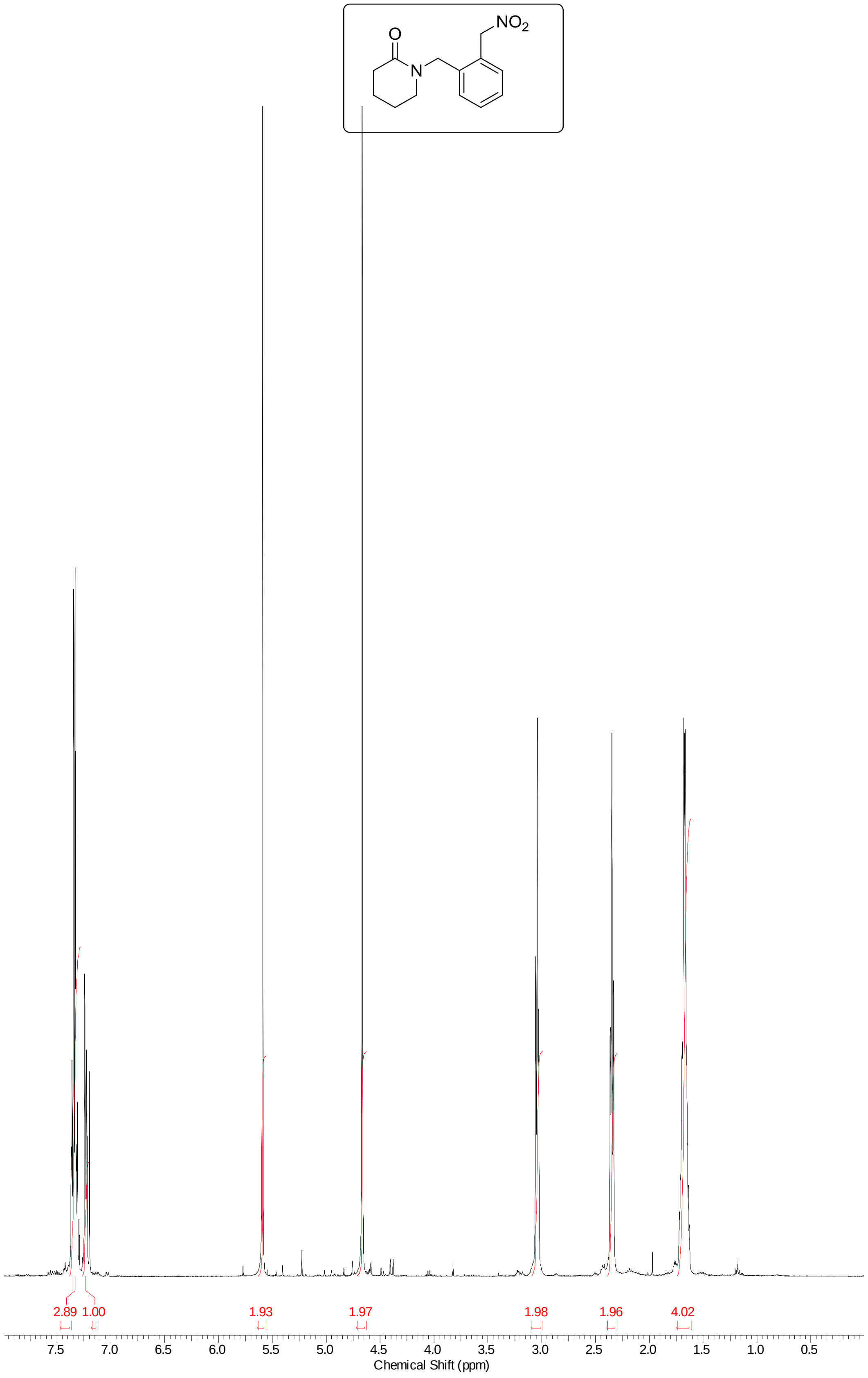

4.1.20 ^{13}CNMR spectrum of 1-[2-(nitromethyl)benzyl]piperidin-2-one (3j)

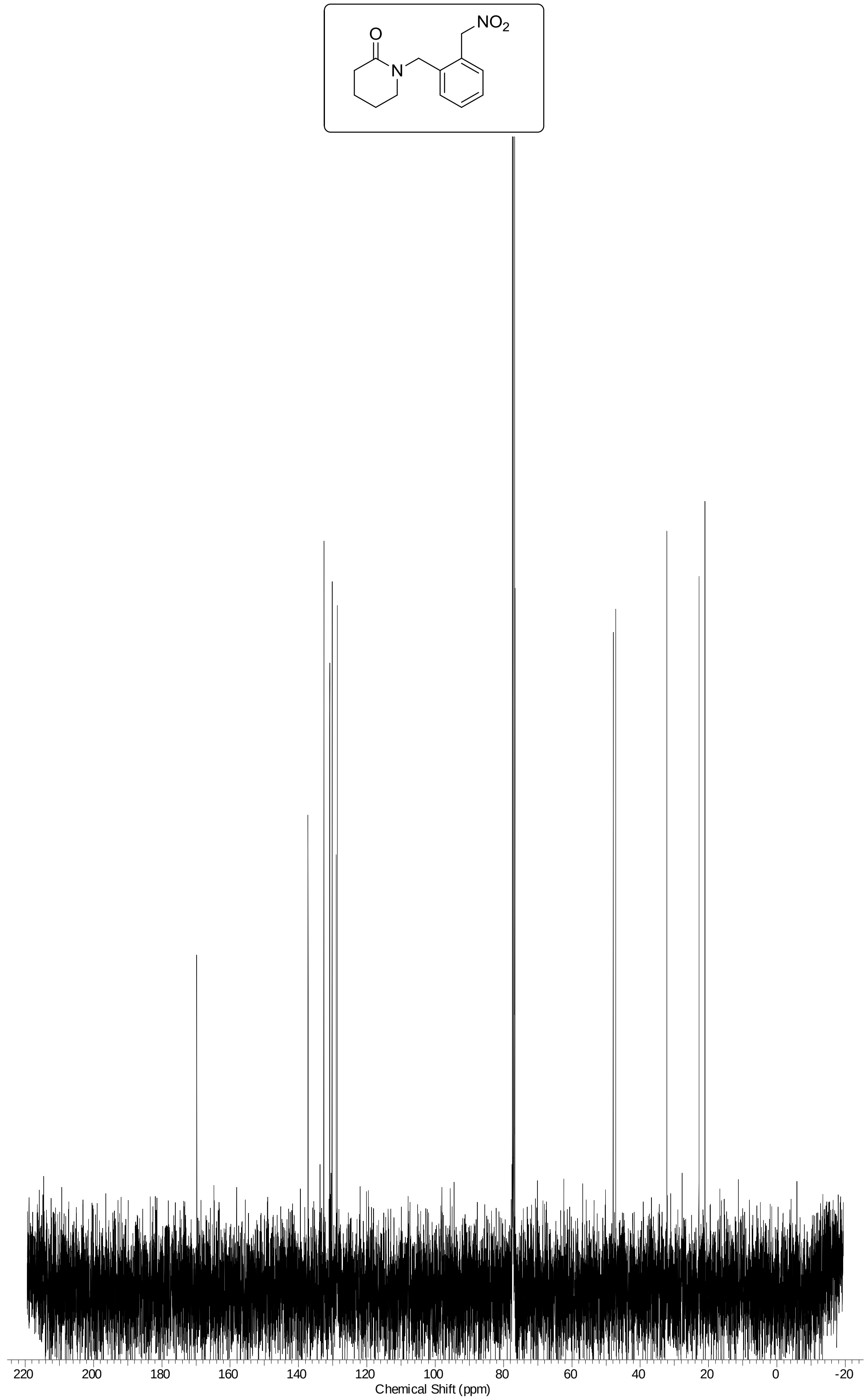

4.1.21 ^{1}HNMR spectrum of 1-[2-(nitromethyl)benzyl]azepan-2-one (3k)

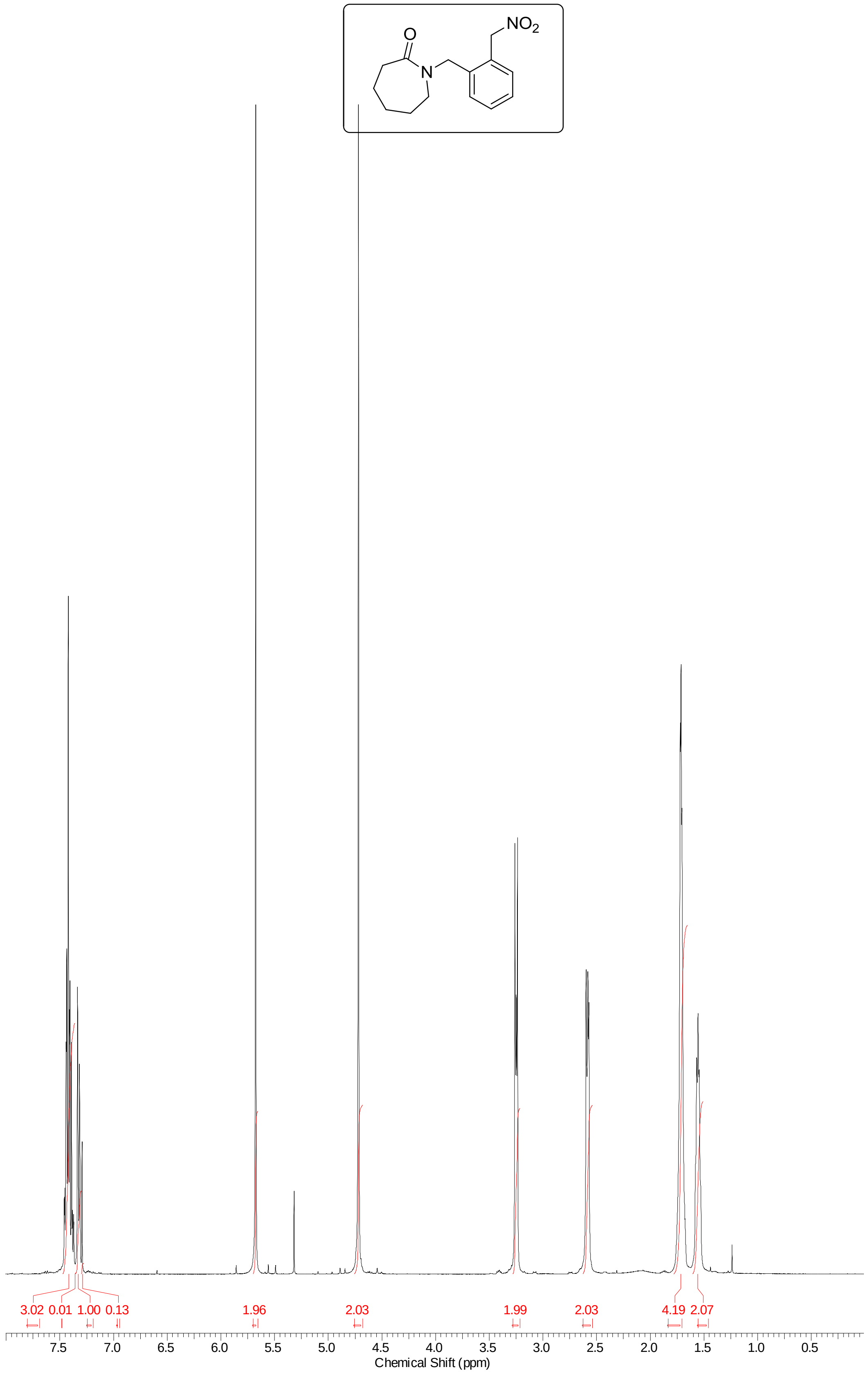

4.1.22 1HNMR spectrum of 1-[2-(nitromethyl)benzyl]azepan-2-one (3k)

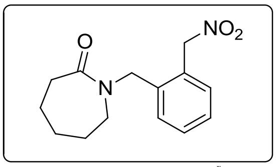

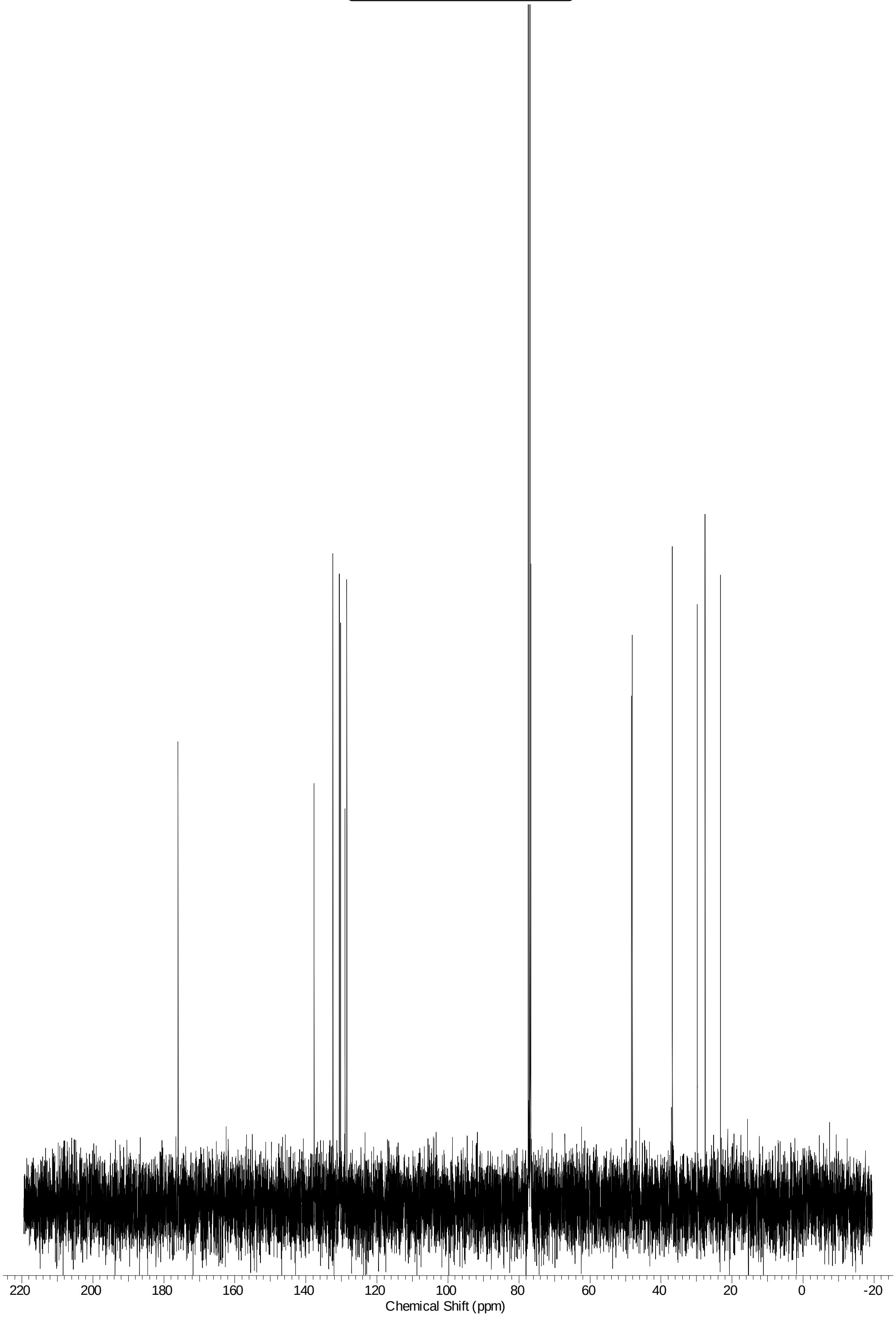

4.1.23 ^{1}HNMR spectrum of 1-[2-(nitromethyl)benzyl]azocan-2-one (3l)

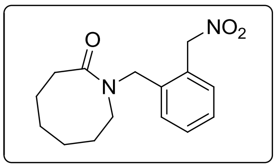

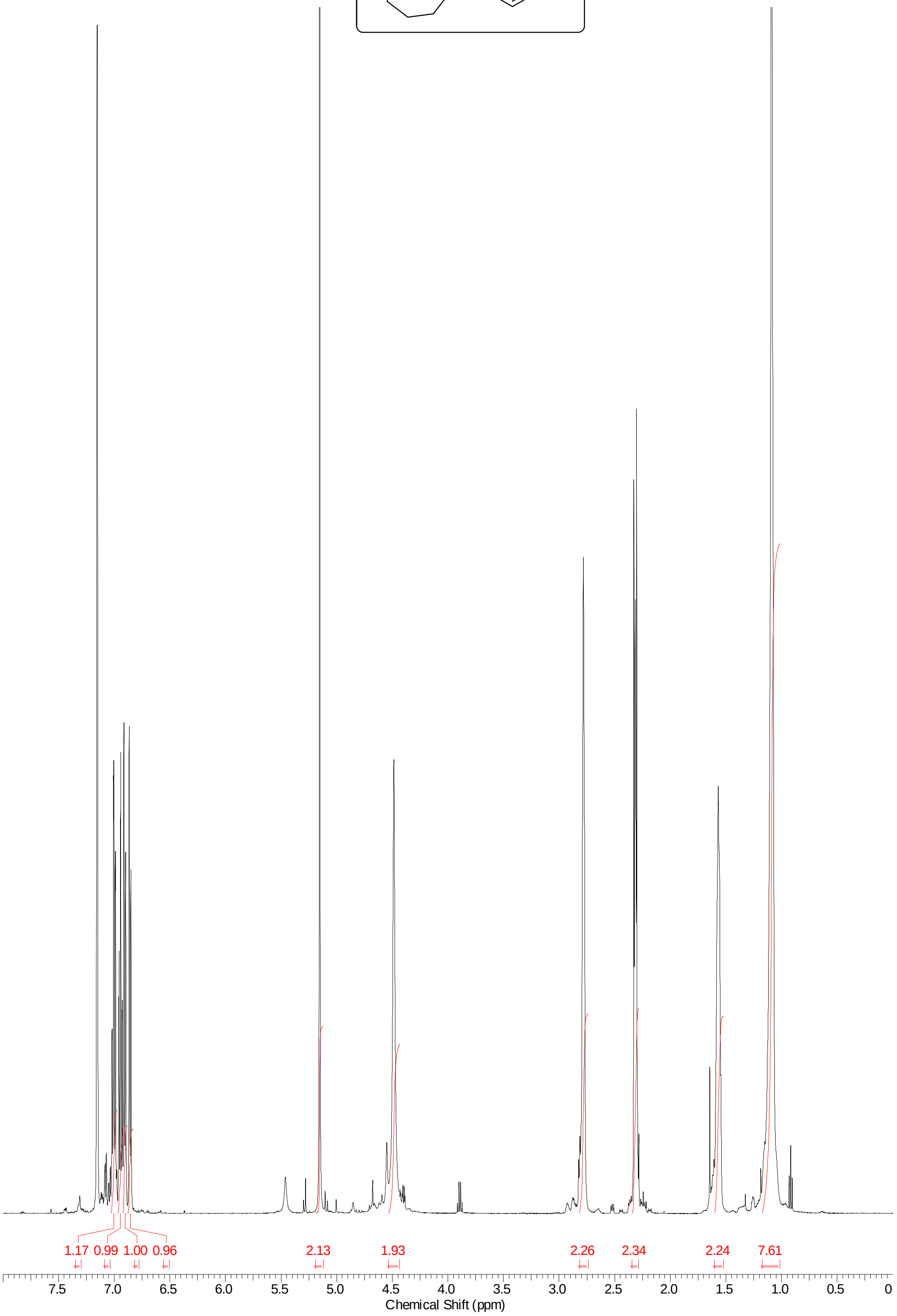

4.1.24 ^{13}CNMR spectrum of 1-[2-(nitromethyl)benzyl]azocan-2-one (3l)

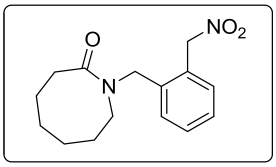

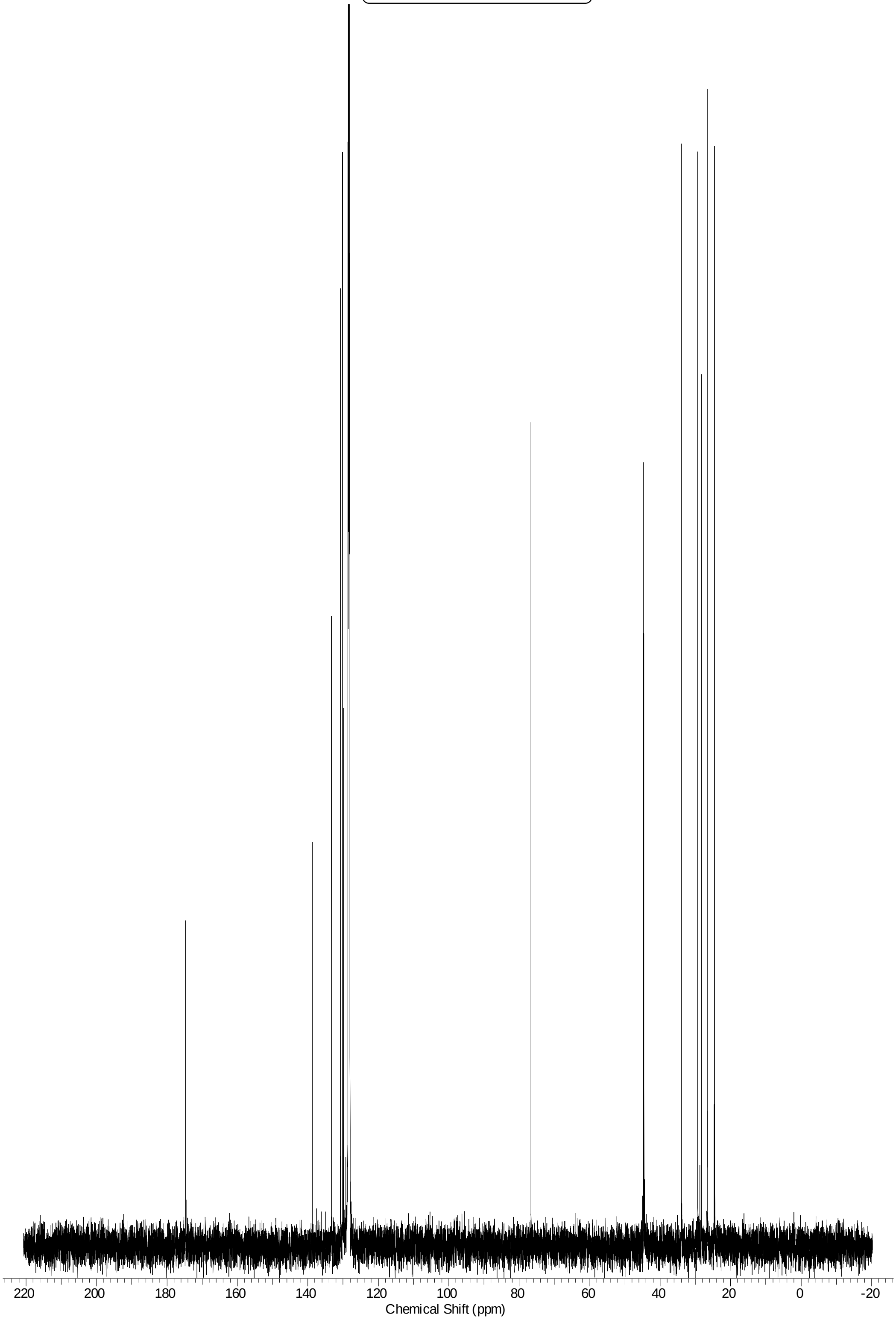

4.2 Cyclized Nitro-Mannich Product Spectra

4.2.1 ^{1}HNMR spectrum of 8-Nitrooctahydroindolizine (5b)

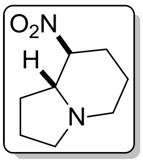

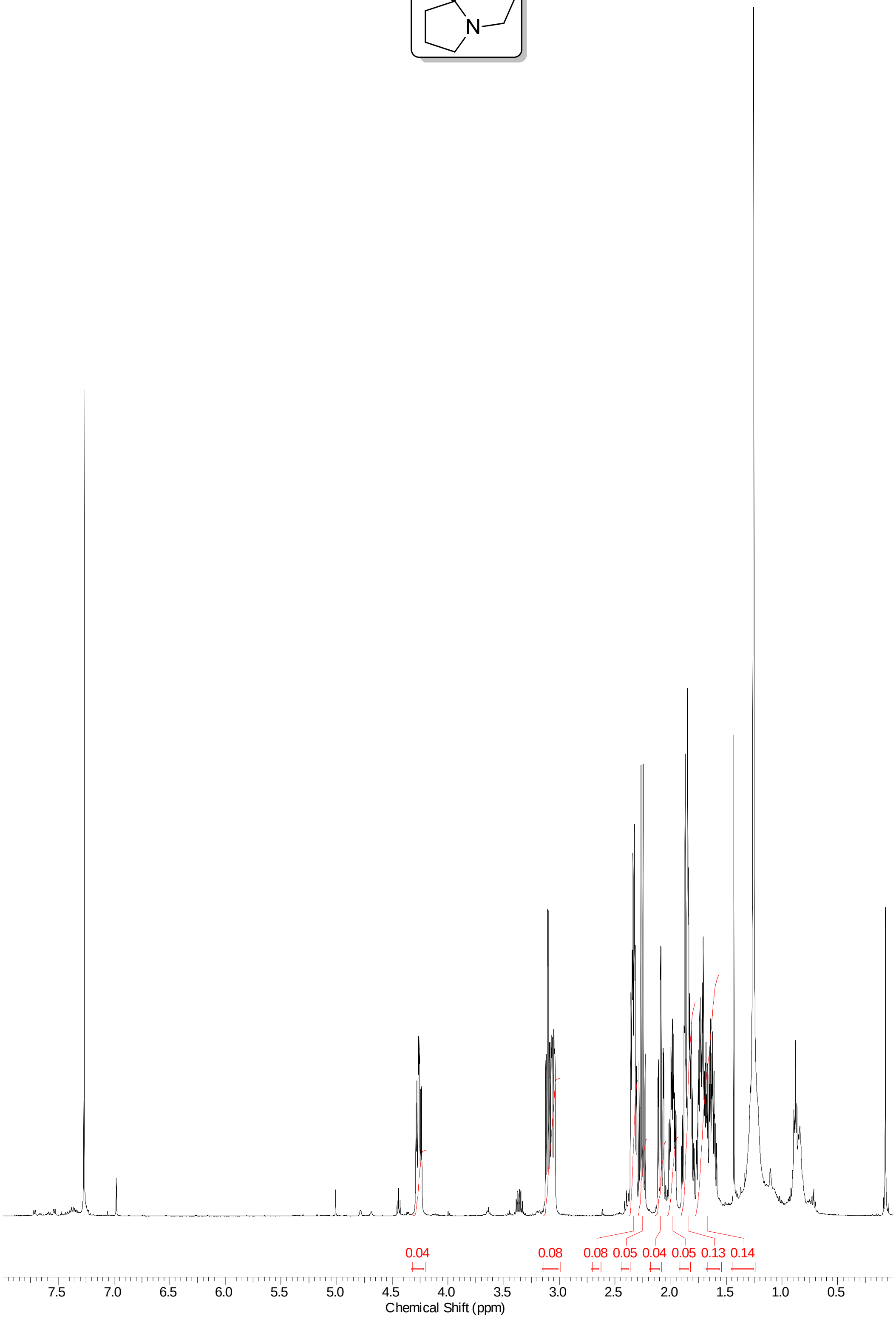

4.2.2 ^{13}CNMR spectrum of 8-Nitrooctahydroindolizine (5b)

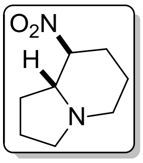

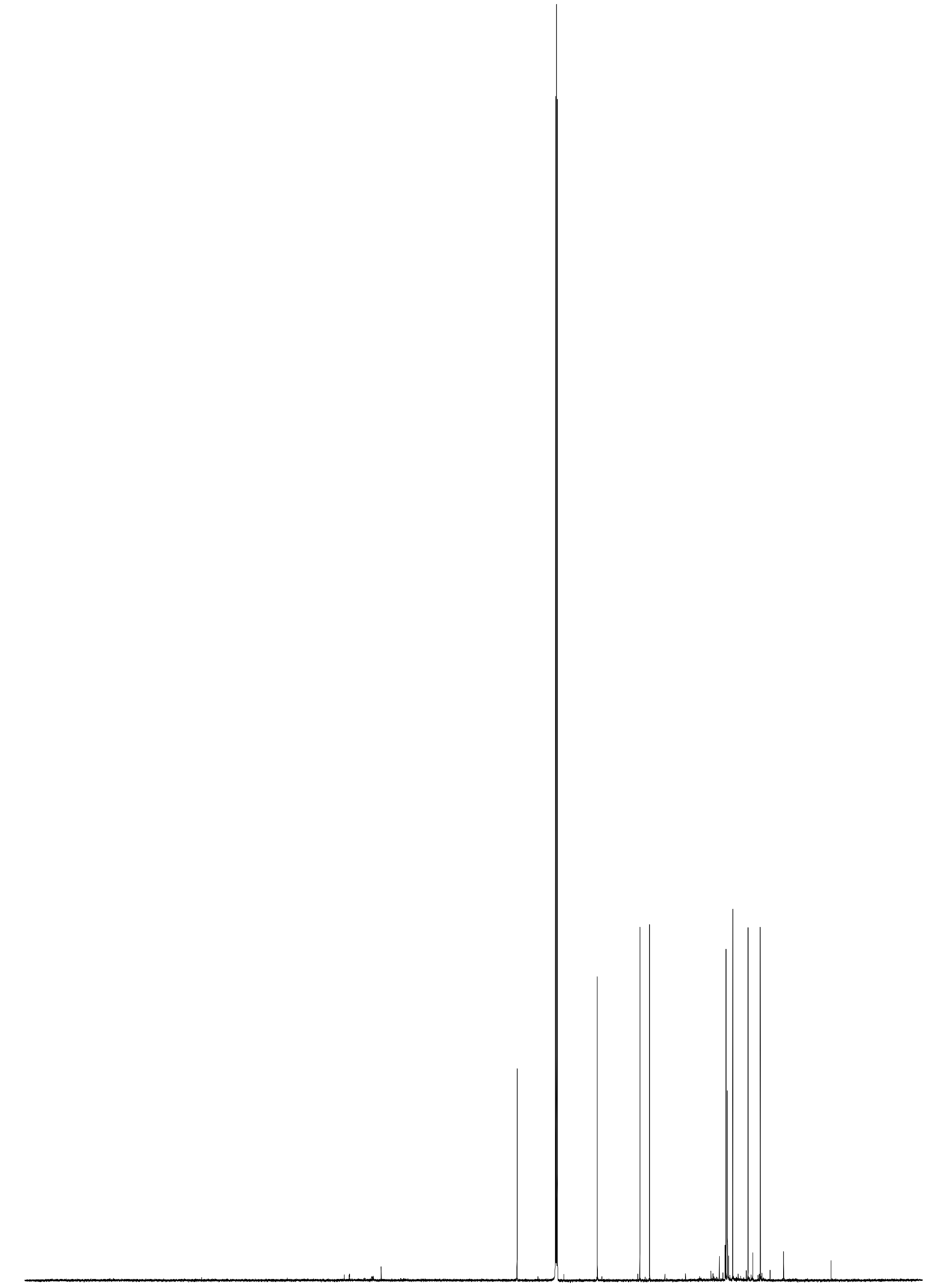

4.2.3 ^{1}HNMR spectrum of 1-Nitrooctahydro-2H-quinolizine (5b)

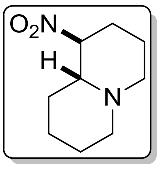

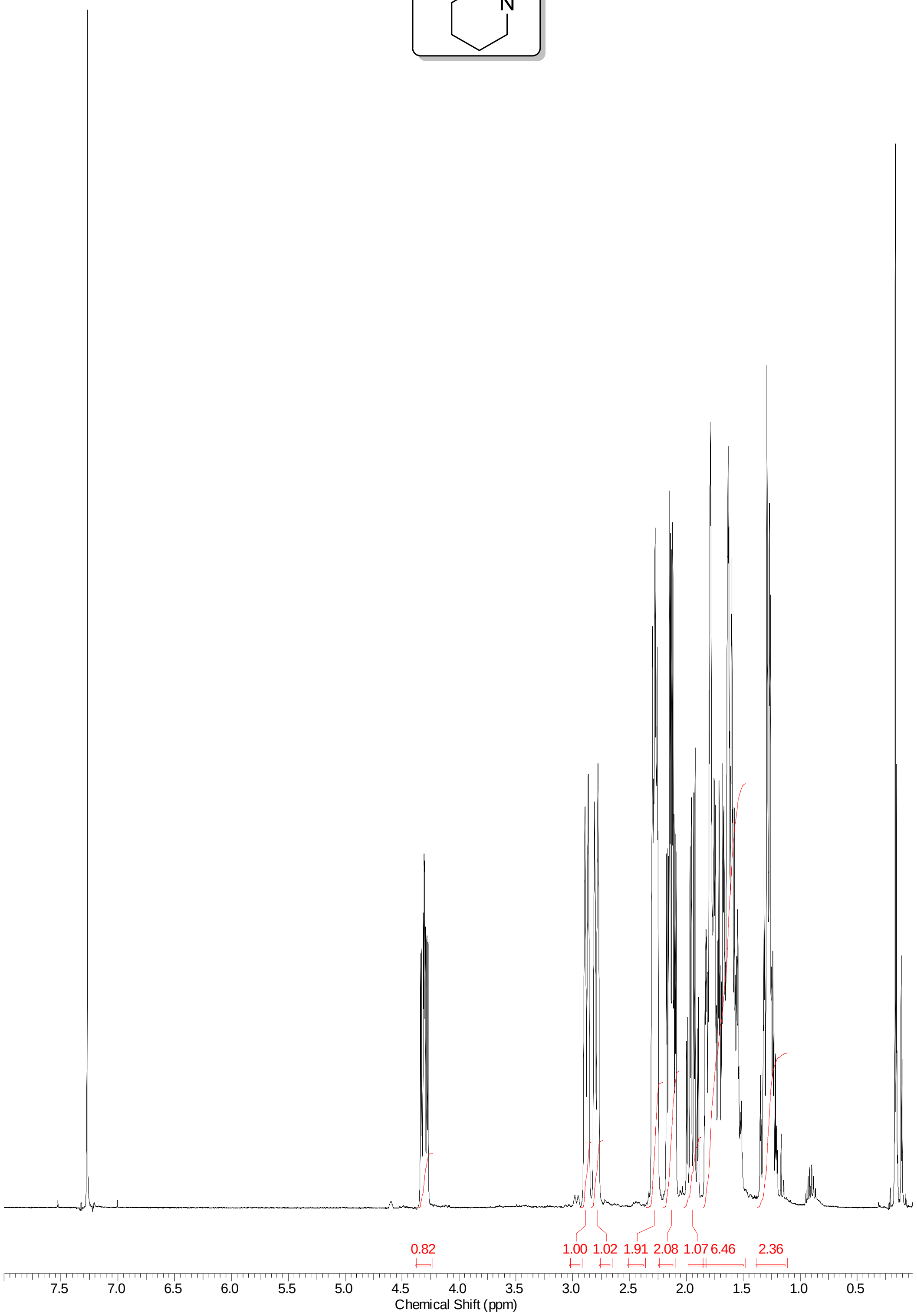

4.2.4 ^{13}CNMR spectrum of 1-Nitrooctahydro-2H-quinolizine (5c)

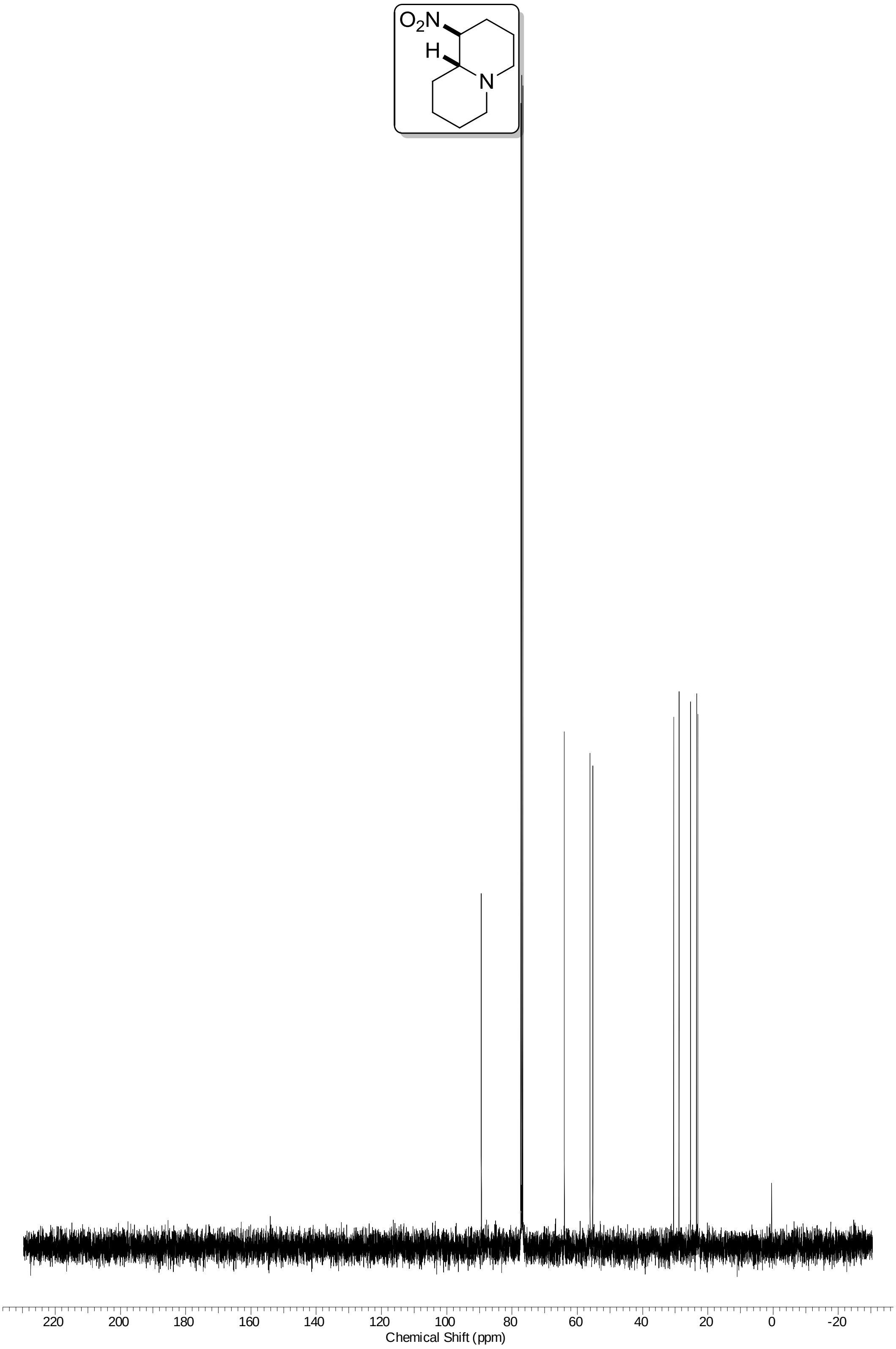

4.2.5 ^{1}HNMR spectrum of 1-Nitrodecahydropyrido[1,2-*a*]azepine (5a)

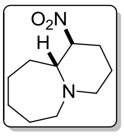

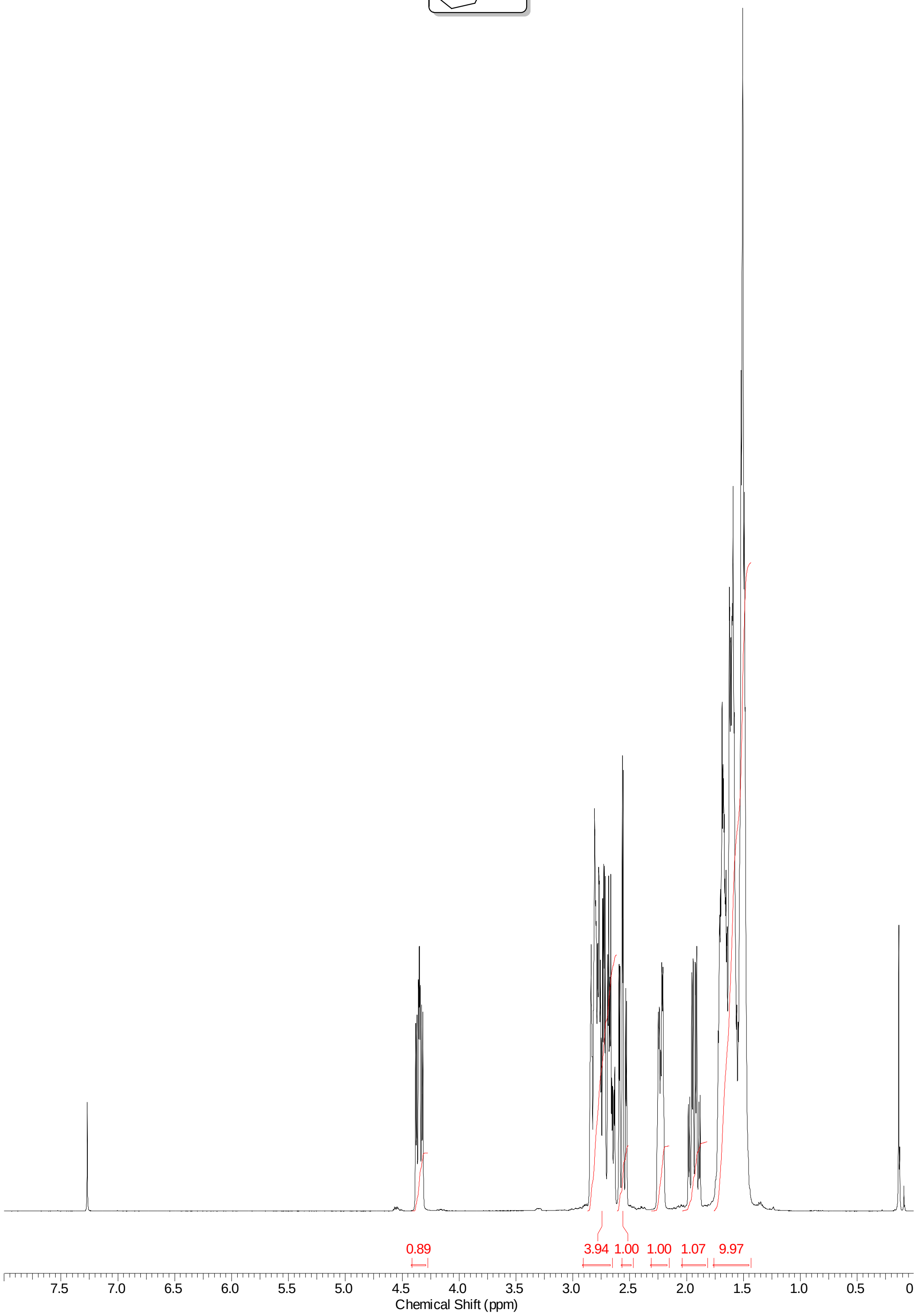

4.2.6 ^{13}CNMR spectrum of 1-Nitrodecahydropyrido[1,2-*a*]azepine (5a)

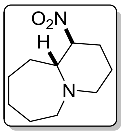

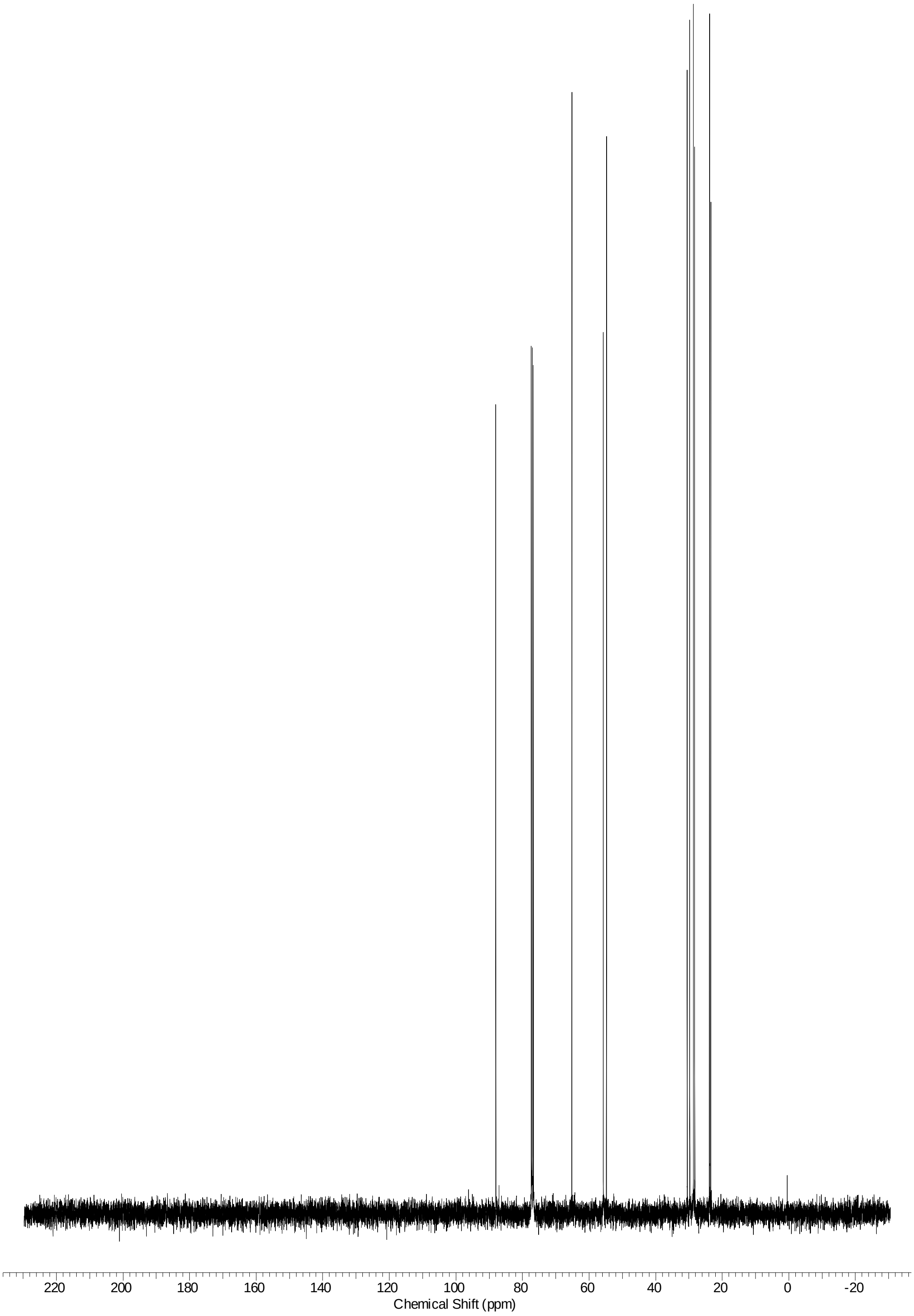

4.2.7 ^{1}HNMR spectrum of 1-Nitrodecahydro-2*H*-pyrido[1,2-*a*]azocine (5d)

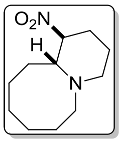

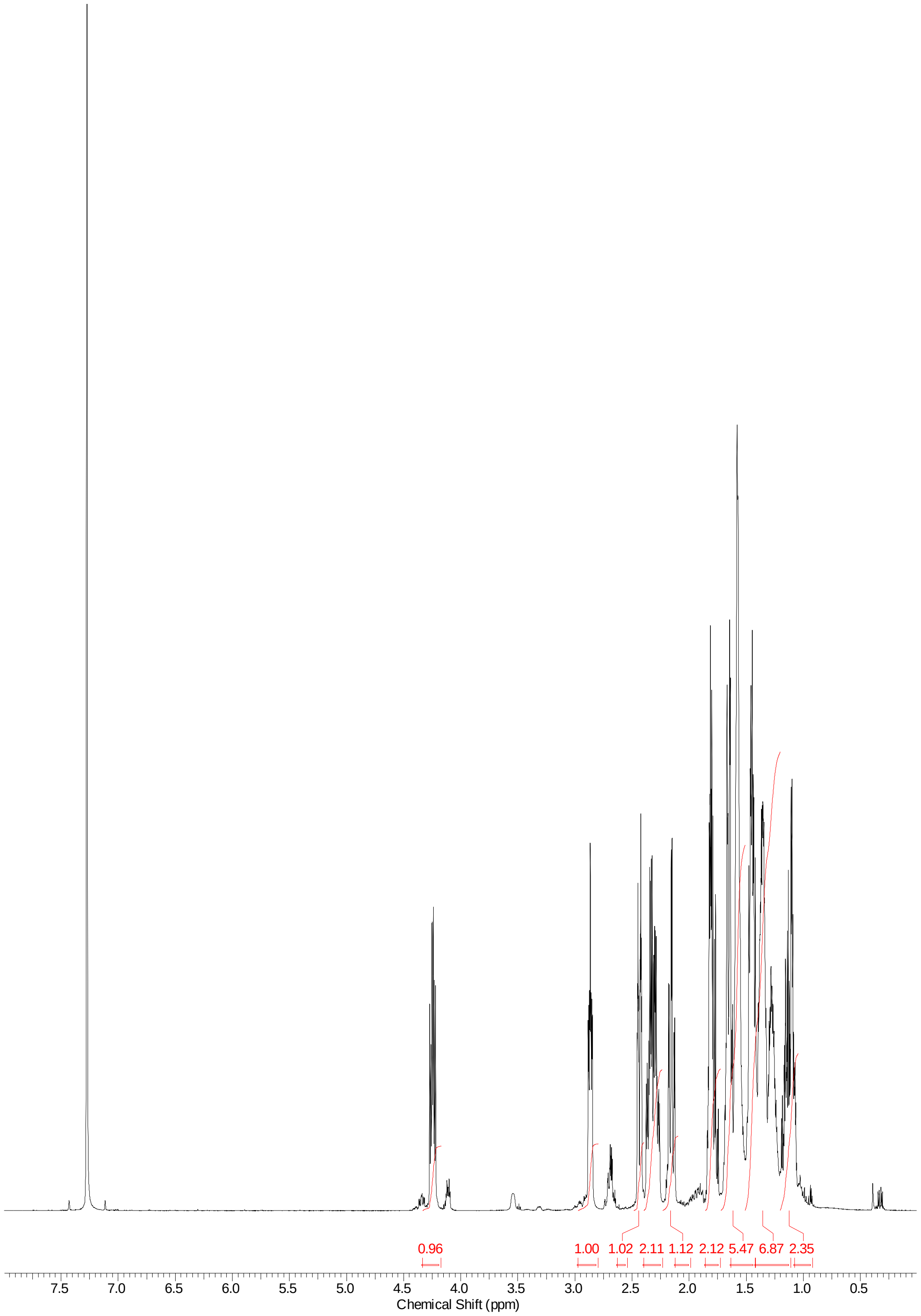

4.2.8 ^{13}CNMR spectrum of 1-Nitrodecahydro-2*H*-pyrido[1,2-*a*]azocine (5d)

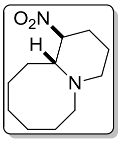

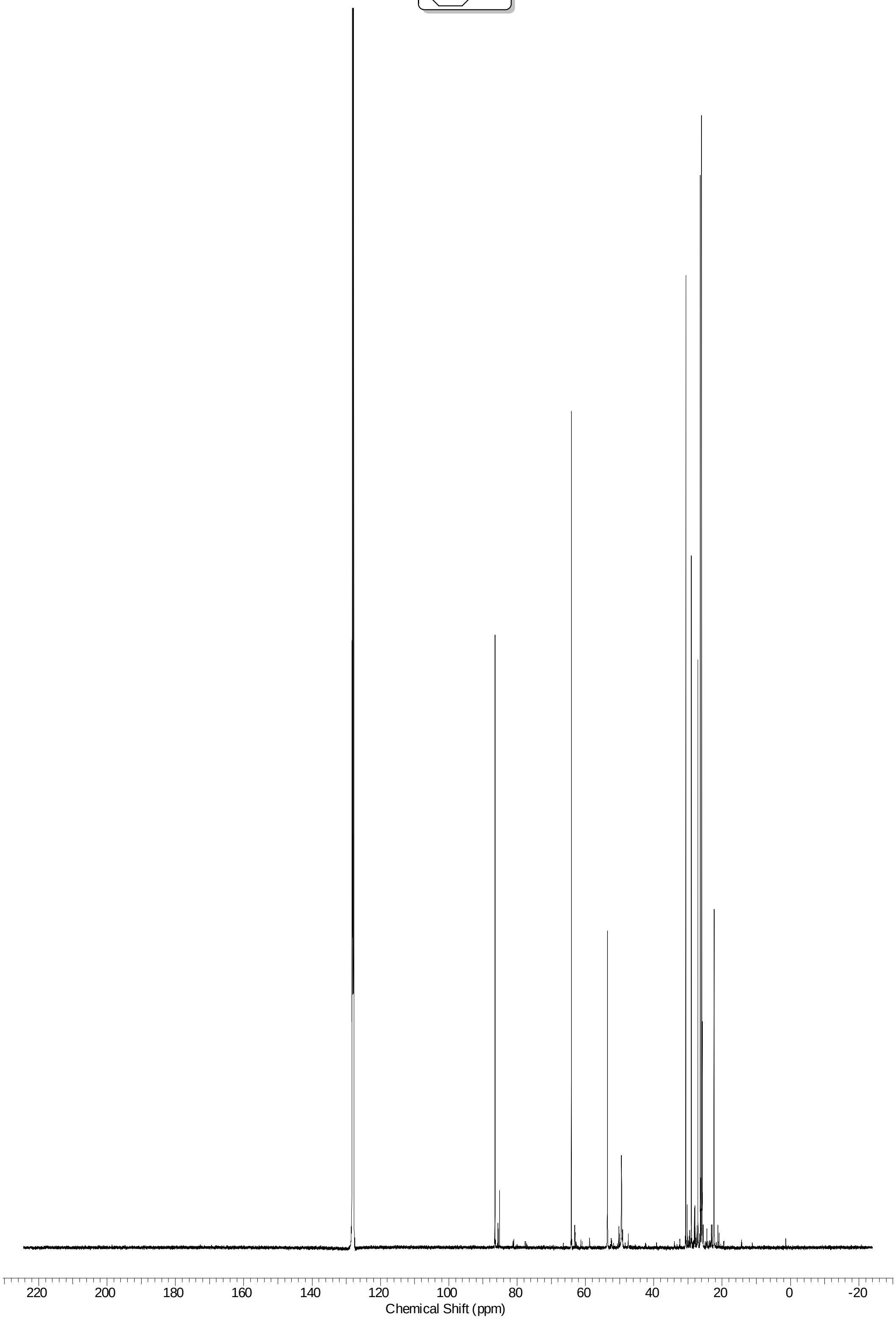

4.2.9 ^{1}HNMR spectrum of 10-nitrooctahydro-2*H*-pyrido[1,2-*d*][1,4]oxazepine (5e)

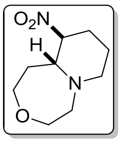

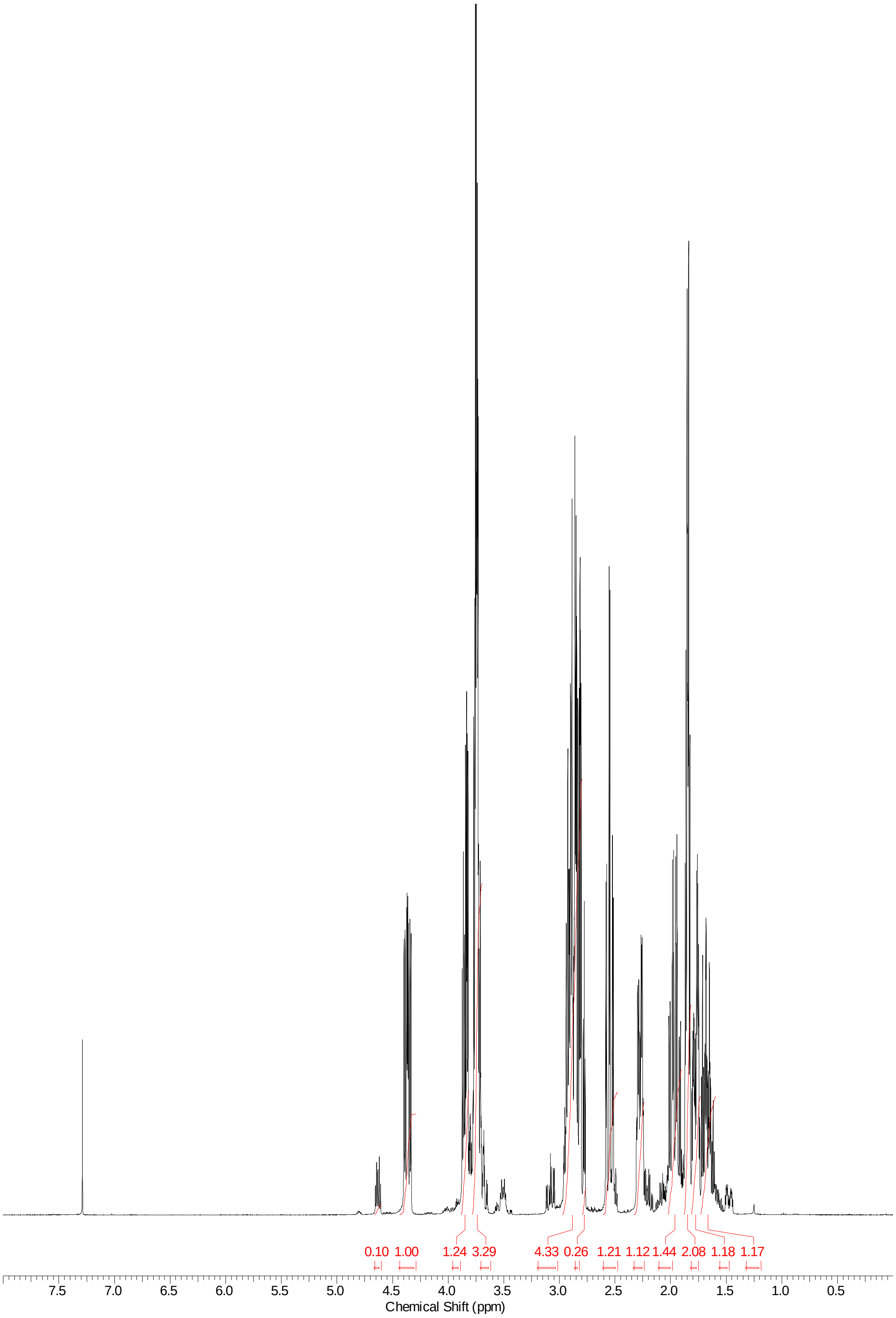

4.2.10 ^{13}CNMR spectrum of 10-nitrooctahydro-2*H*-pyrido[1,2-*d*][1,4]oxazepine (5e)

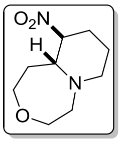

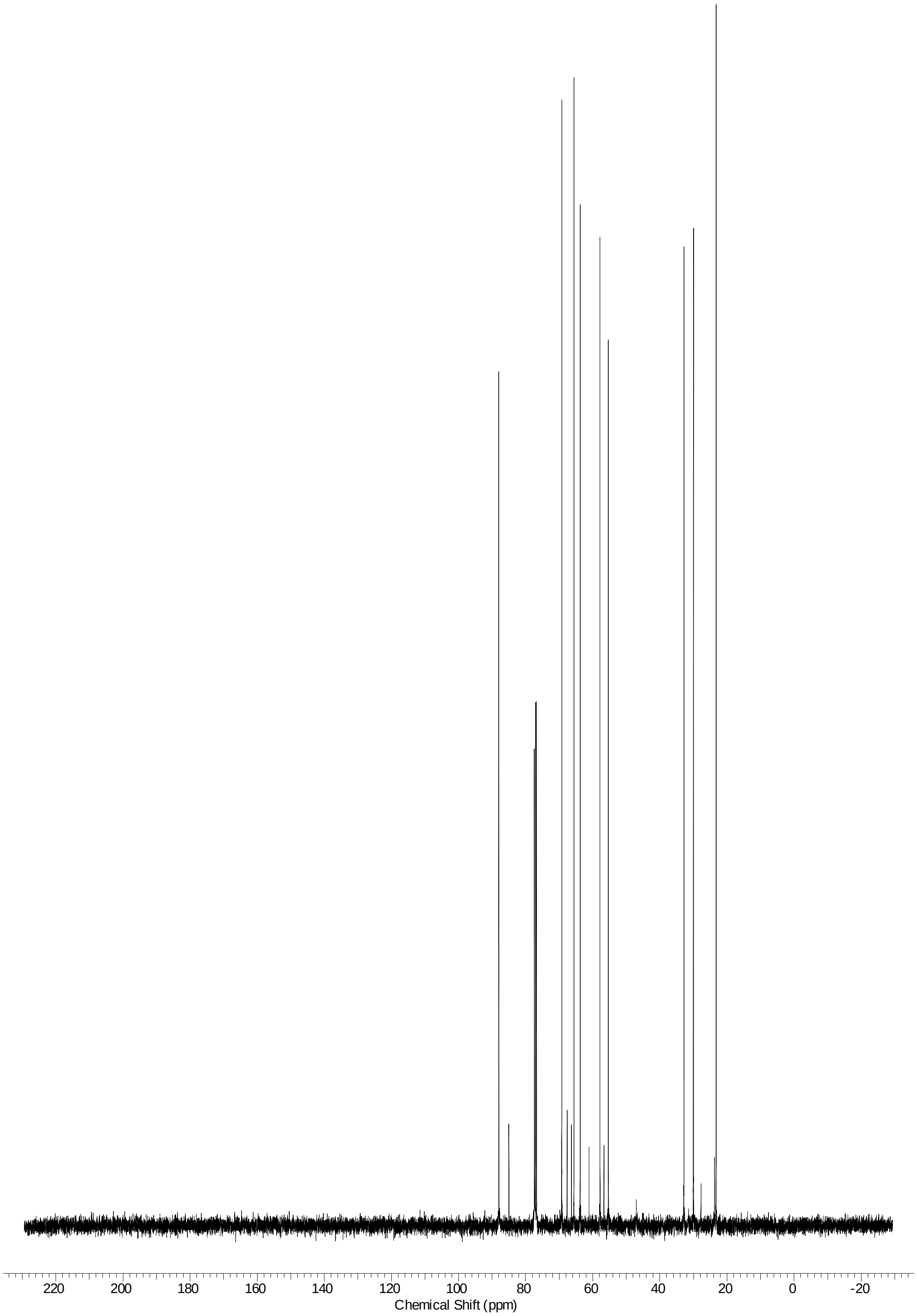

4.2.11 ^{1}HNMR spectrum of 1-Nitrodecahydro-1*H*-azepino[1,2-*a*]azepine (5f)

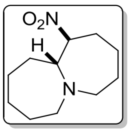

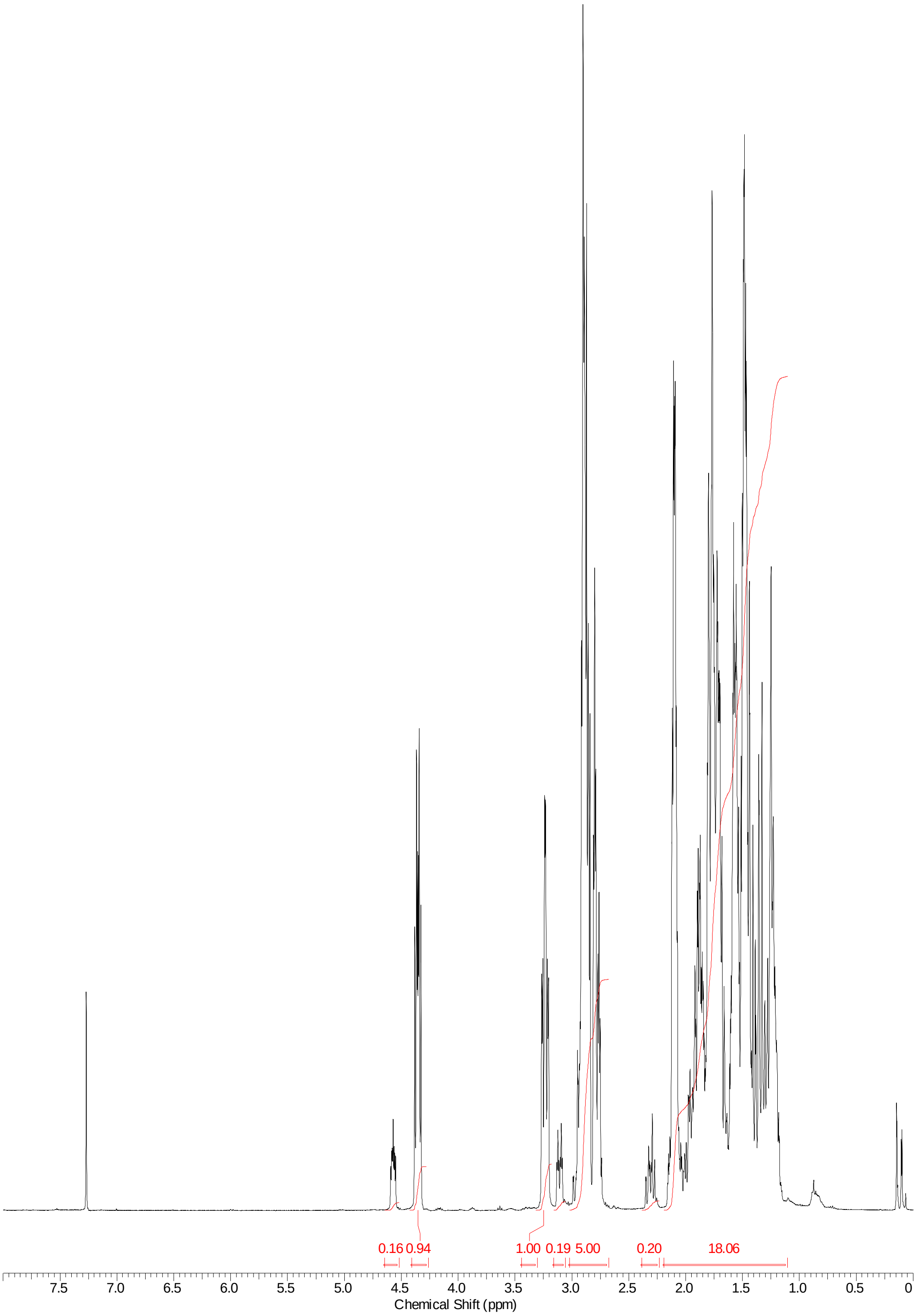

4.2.12 ^{13}CNMR spectrum of 1-Nitrodecahydro-1*H*-azepino[1,2-*a*]azepine (5f)

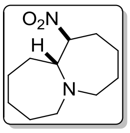

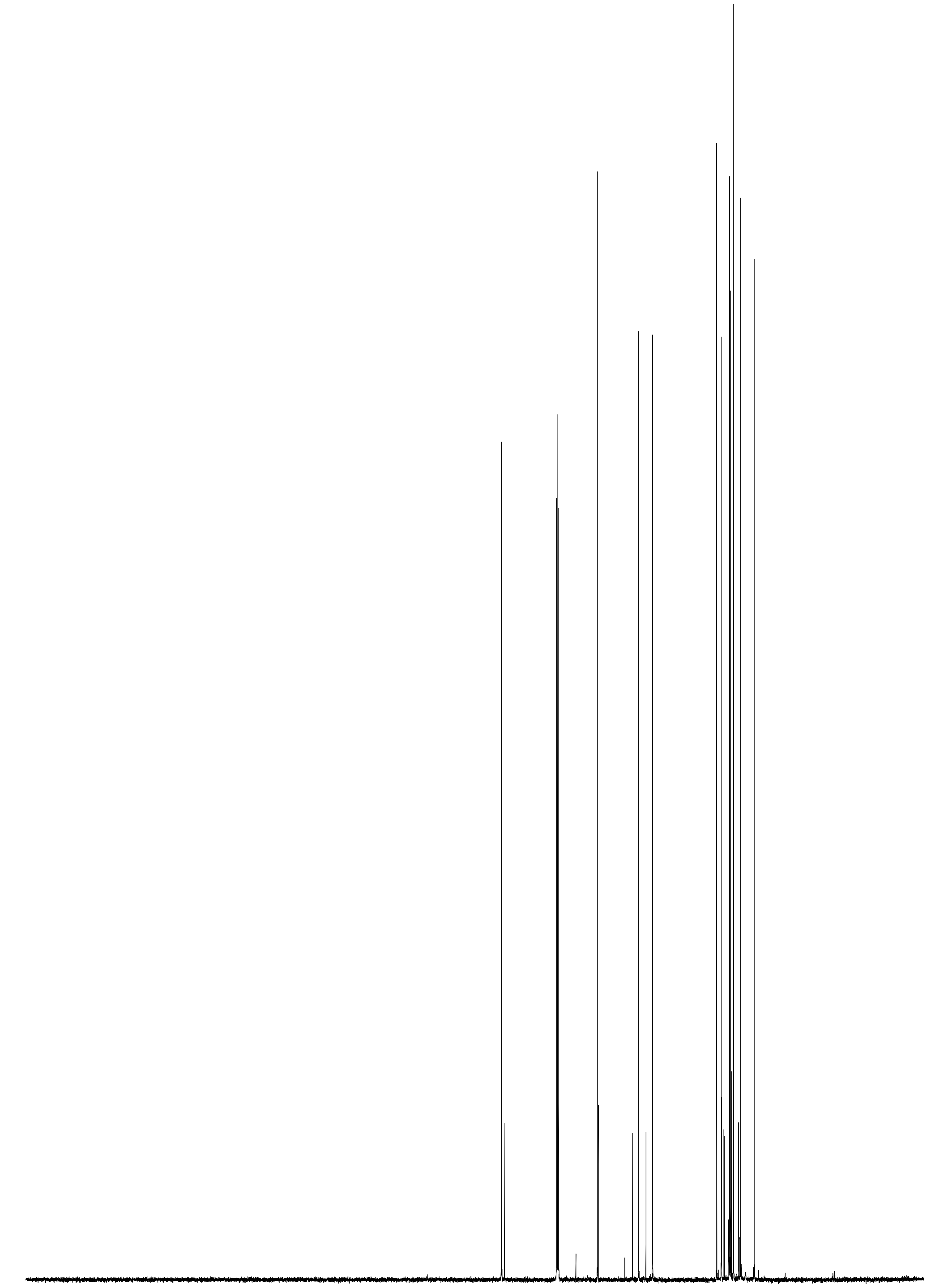

4.2.13 NOE spectrum of 1-Nitrodecahydro-1*H*-azepino[1,2-*a*]azepine (5f)

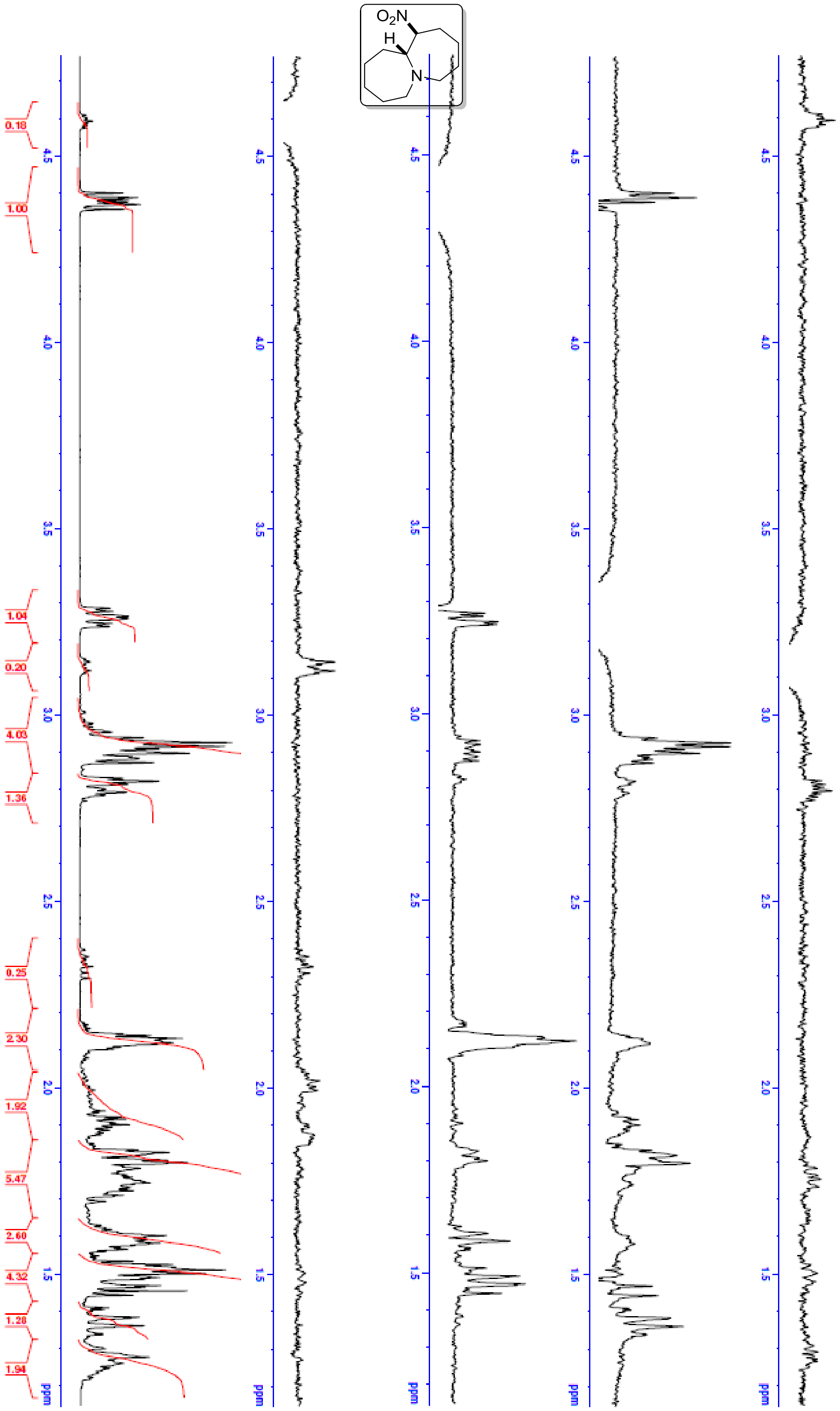

4.2.14 NOESY spectrum of 1-Nitrodecahydro-1*H*-azepino[1,2-*a*]azepine (5f)

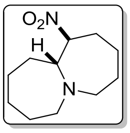

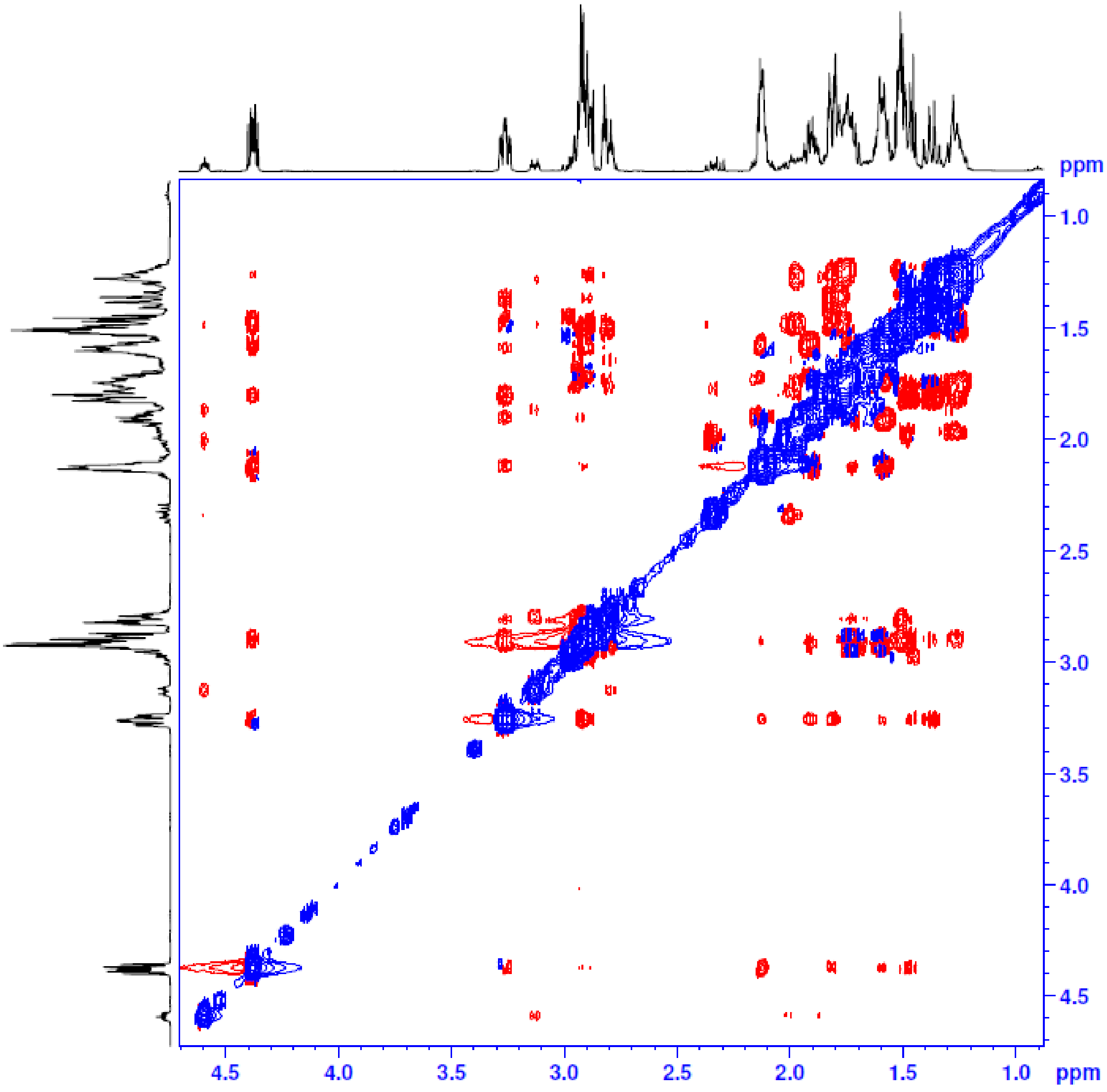

4.2.15 ^{1}HNMR spectrum of 9-Nitrooctahydro-1H-pyrrolo[1,2-*a*]azepine (5g)

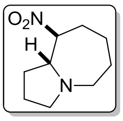

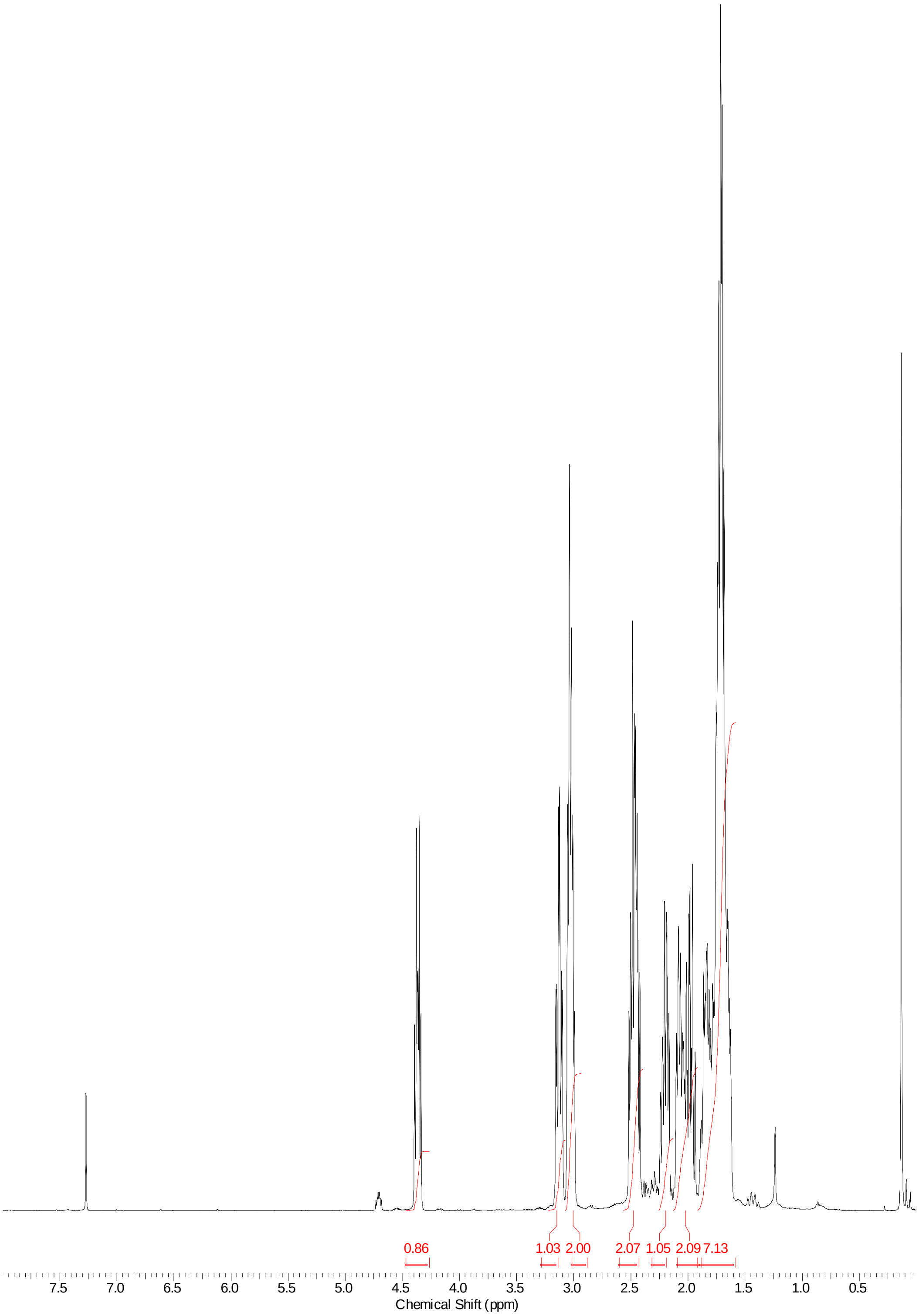

4.2.16 ^{13}CNMR spectrum of 9-Nitrooctahydro-1*H*-pyrrolo[1,2-*a*]azepine (5g)

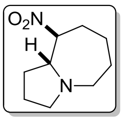

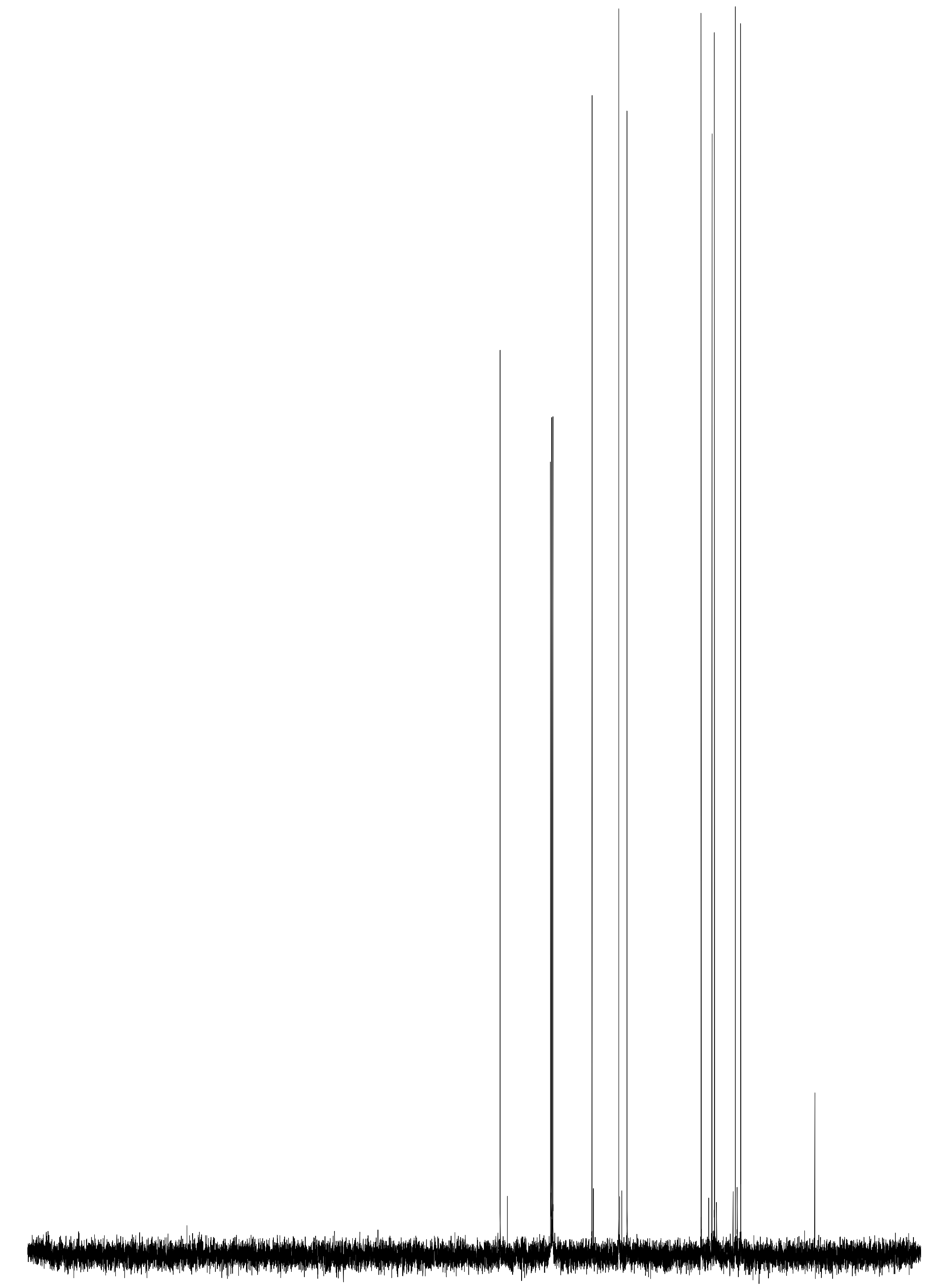

4.2.17 ^{1}HNMR spectrum of 1-nitrooctahydro-1*H*-pyrrolo[1,2-*a*]azepine (5h)

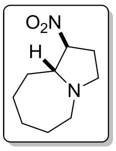

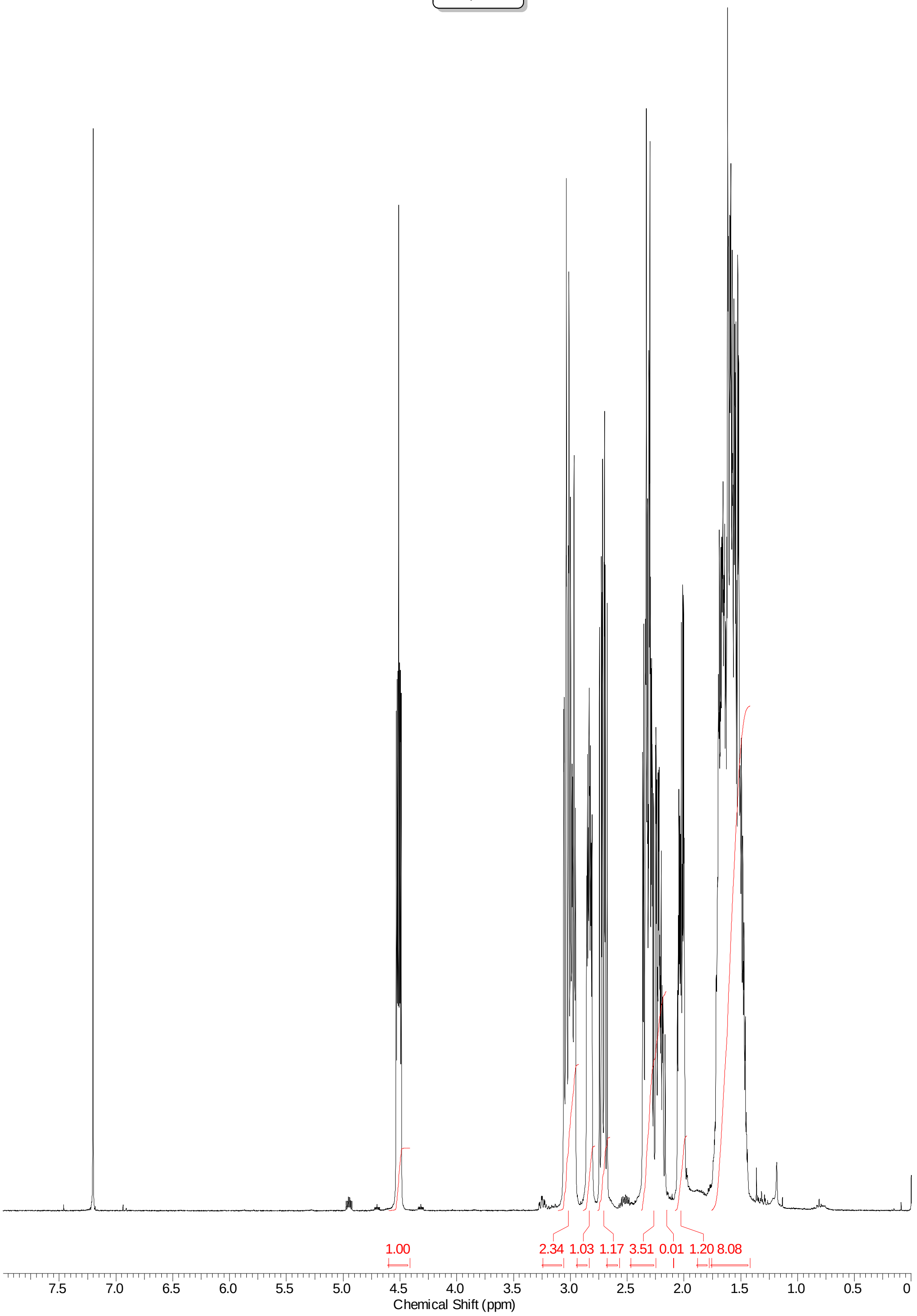

4.2.18 ^{13}CNMR spectrum of 1-nitrooctahydro-1*H*-pyrrolo[1,2-*a*]azepine (5h)

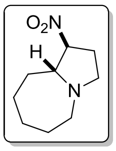

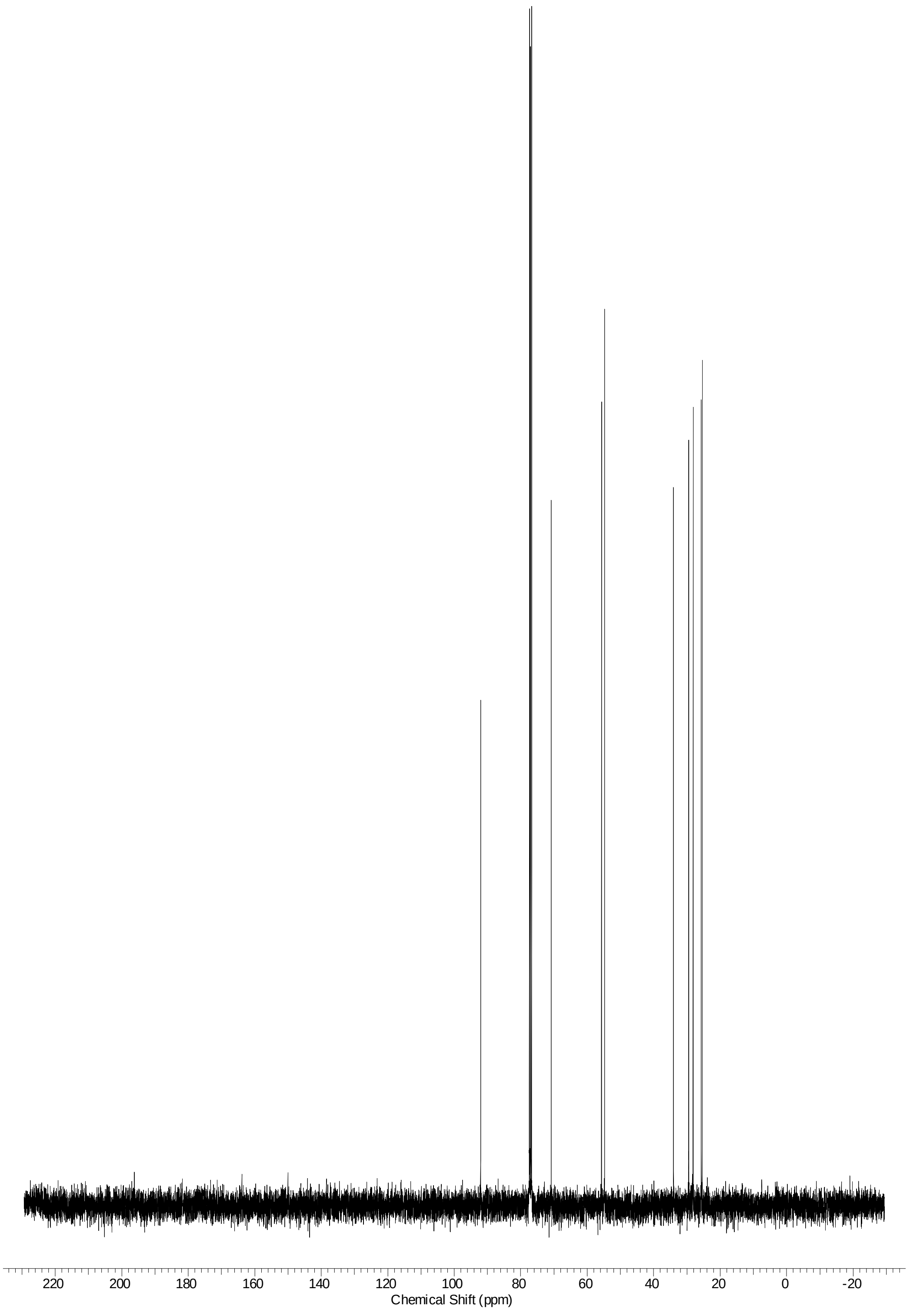

4.2.19 ^{1}HNMR spectrum of 1-nitrooctahydro-1*H*-pyrrolo[1,2-*a*]azepine mixed diastereomers (5h)

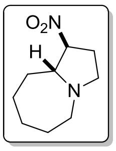

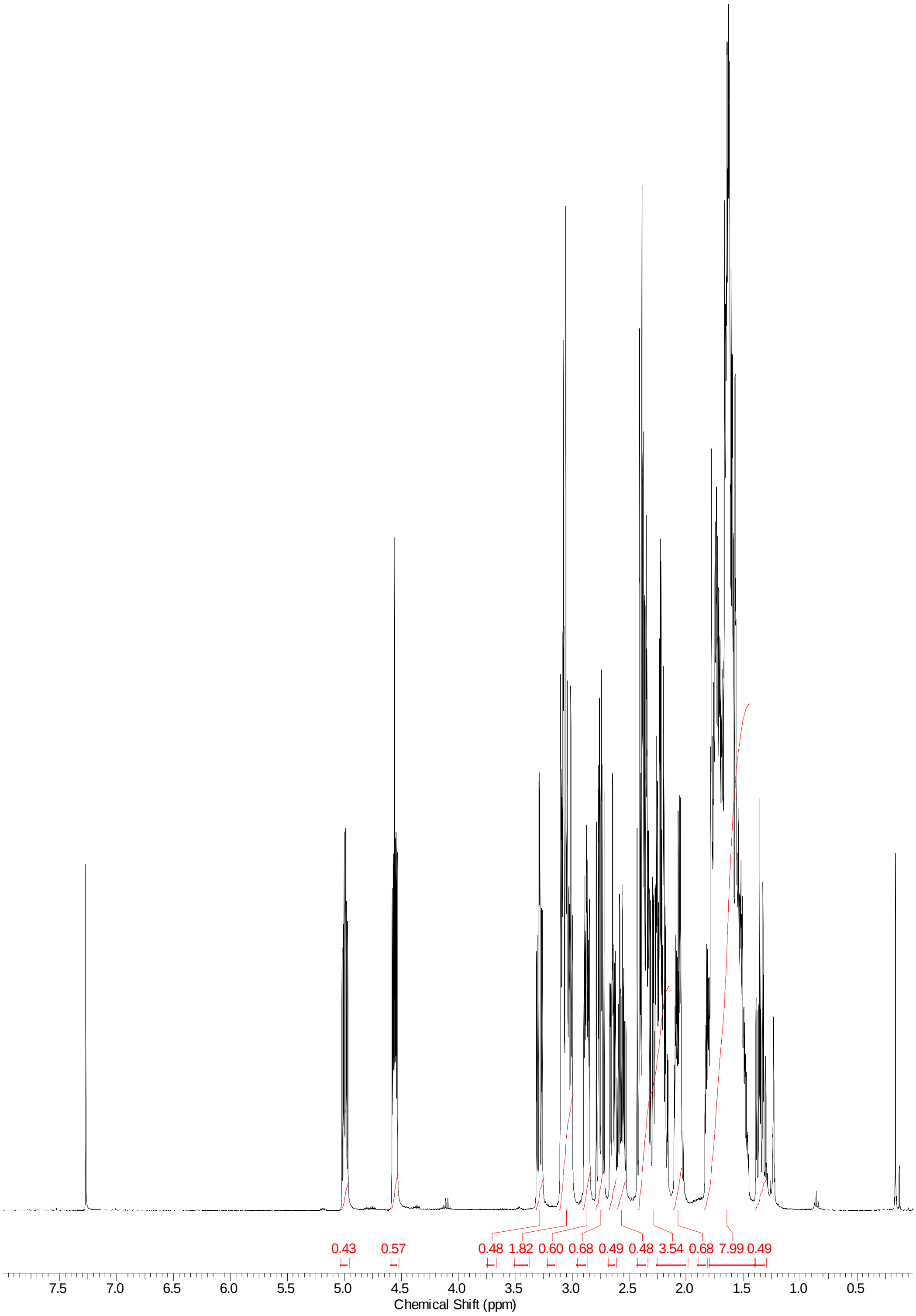

4.2.20 ^{13}CNMR spectrum of 1-nitrooctahydro-1*H*-pyrrolo[1,2-*a*]azepine mixed diastereomers (5h)

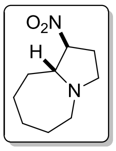

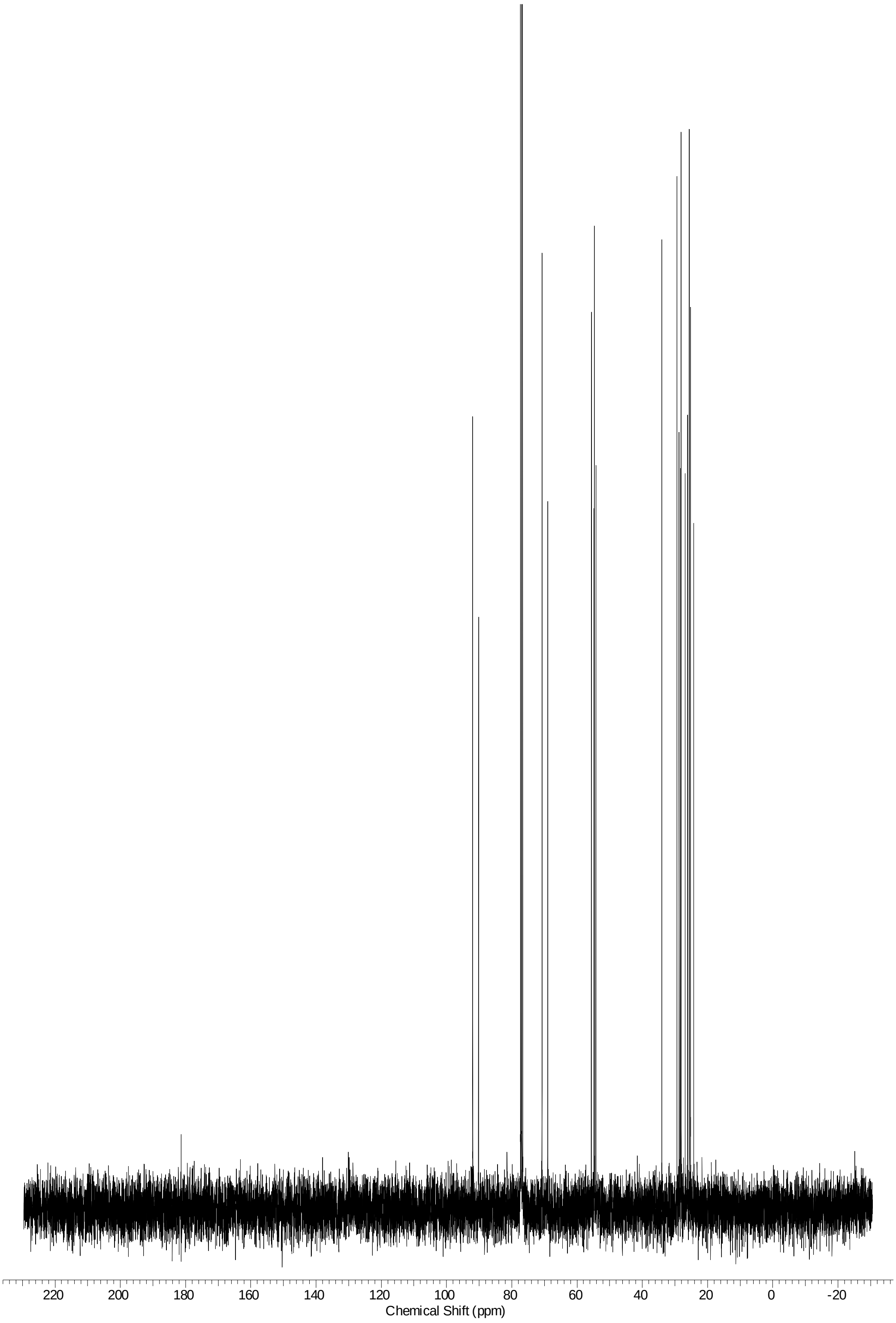

4.2.21 ^{1}HNMR spectrum of 1-nitrodecahydropyrrolo[1,2-*a*]azocine (5i)

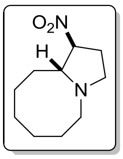

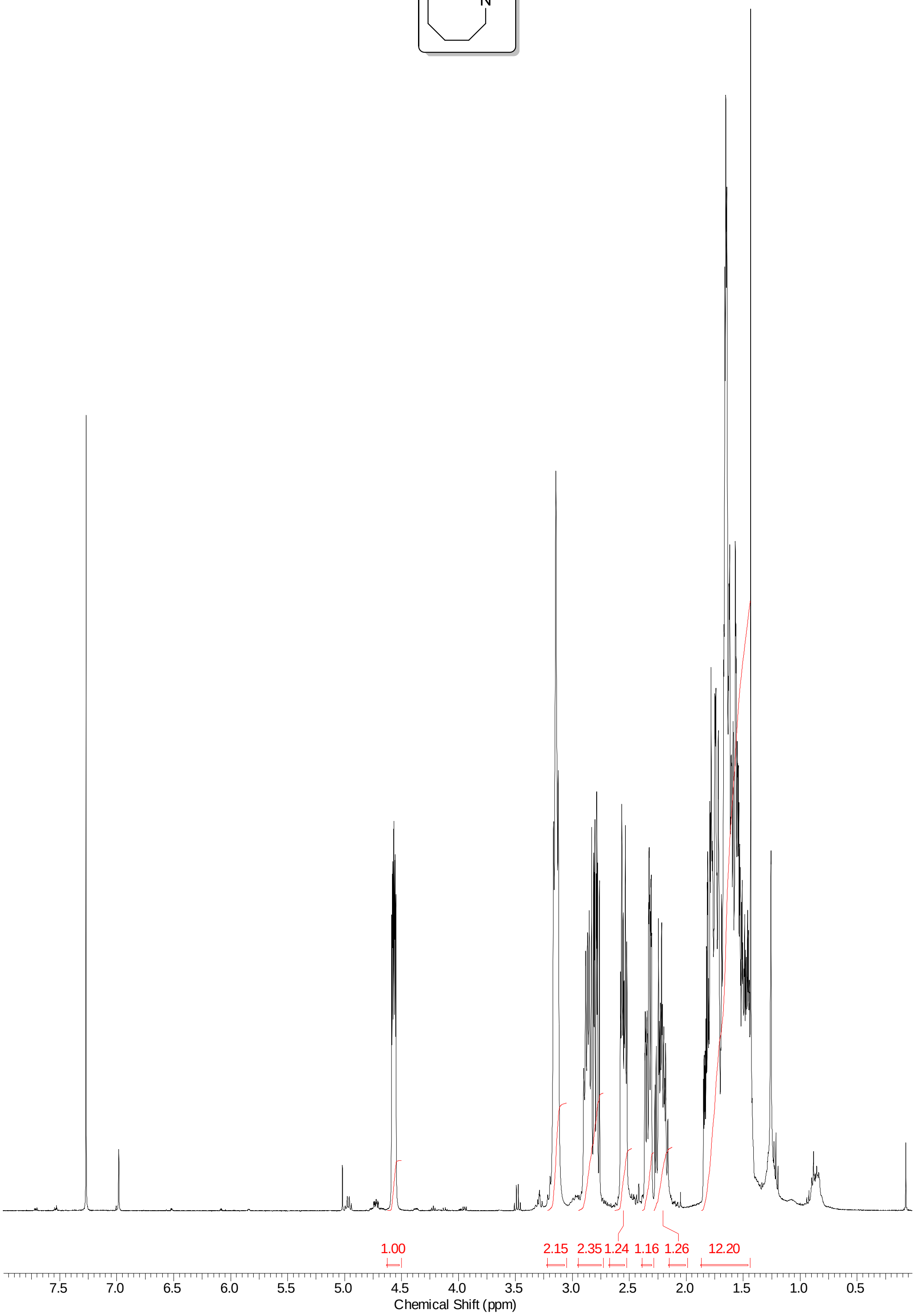

4.2.22 ^{13}CNMR spectrum of 1-nitrodecahydropyrrolo[1,2-*a*]azocine (5i)

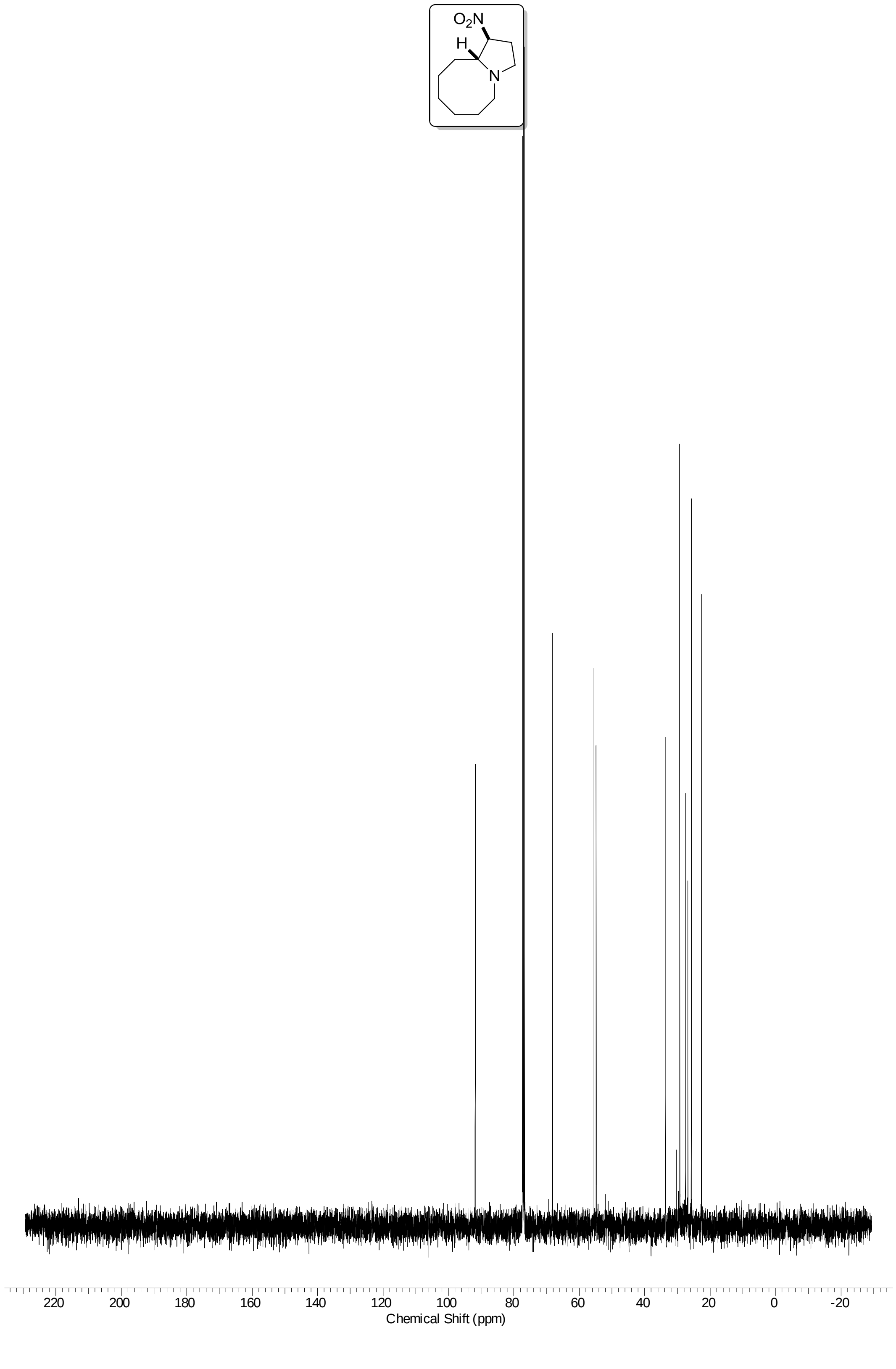

4.2.23 ^{1}HNMR spectrum of 1-nitrodecahydropyrrolo[1,2-*a*]azocine mixed diastereomer (5i)

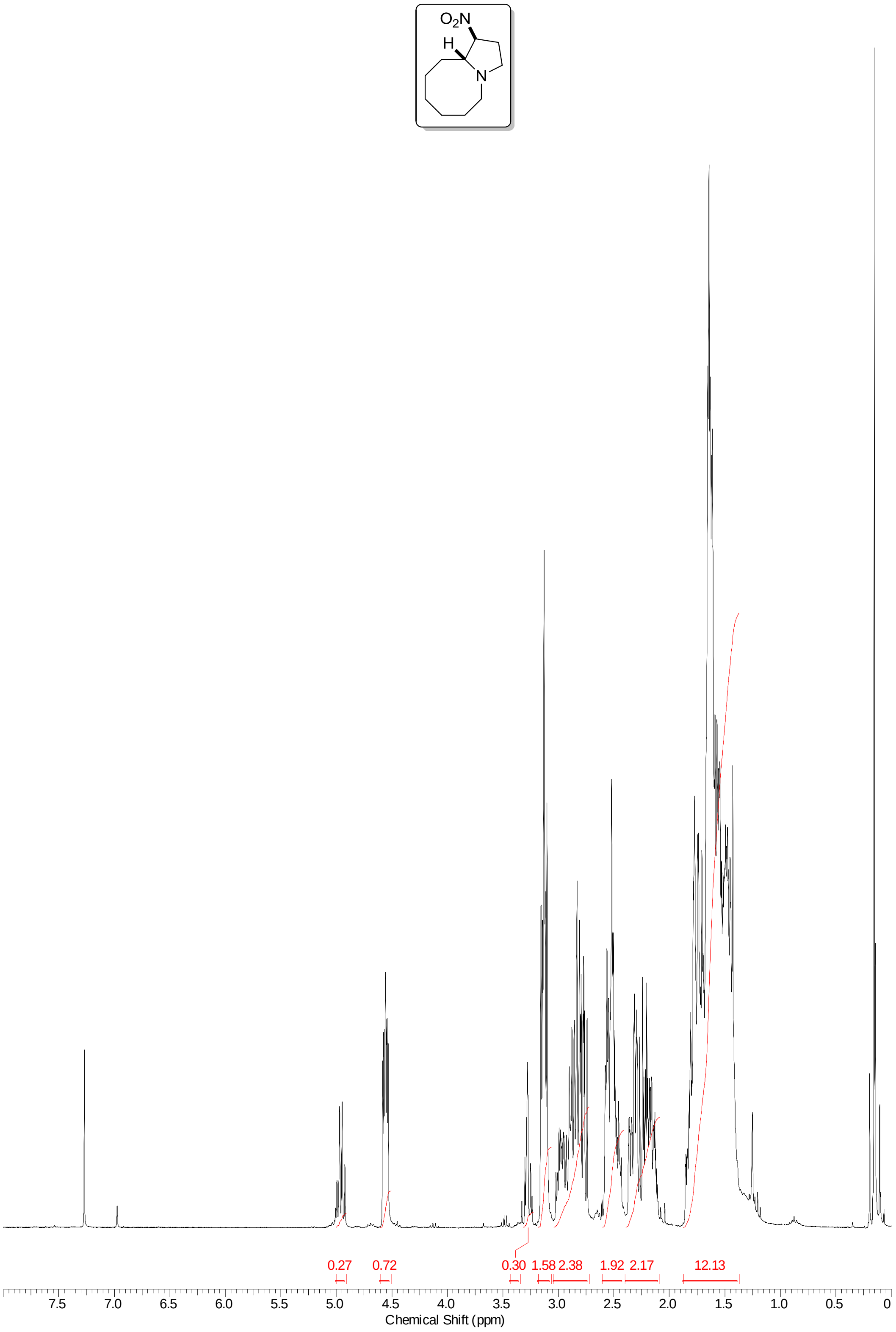

4.2.24 ^{13}CNMR spectrum of 1-nitrodecahydropyrrolo[1,2-*a*]azocine mixed diastereomer (5i)

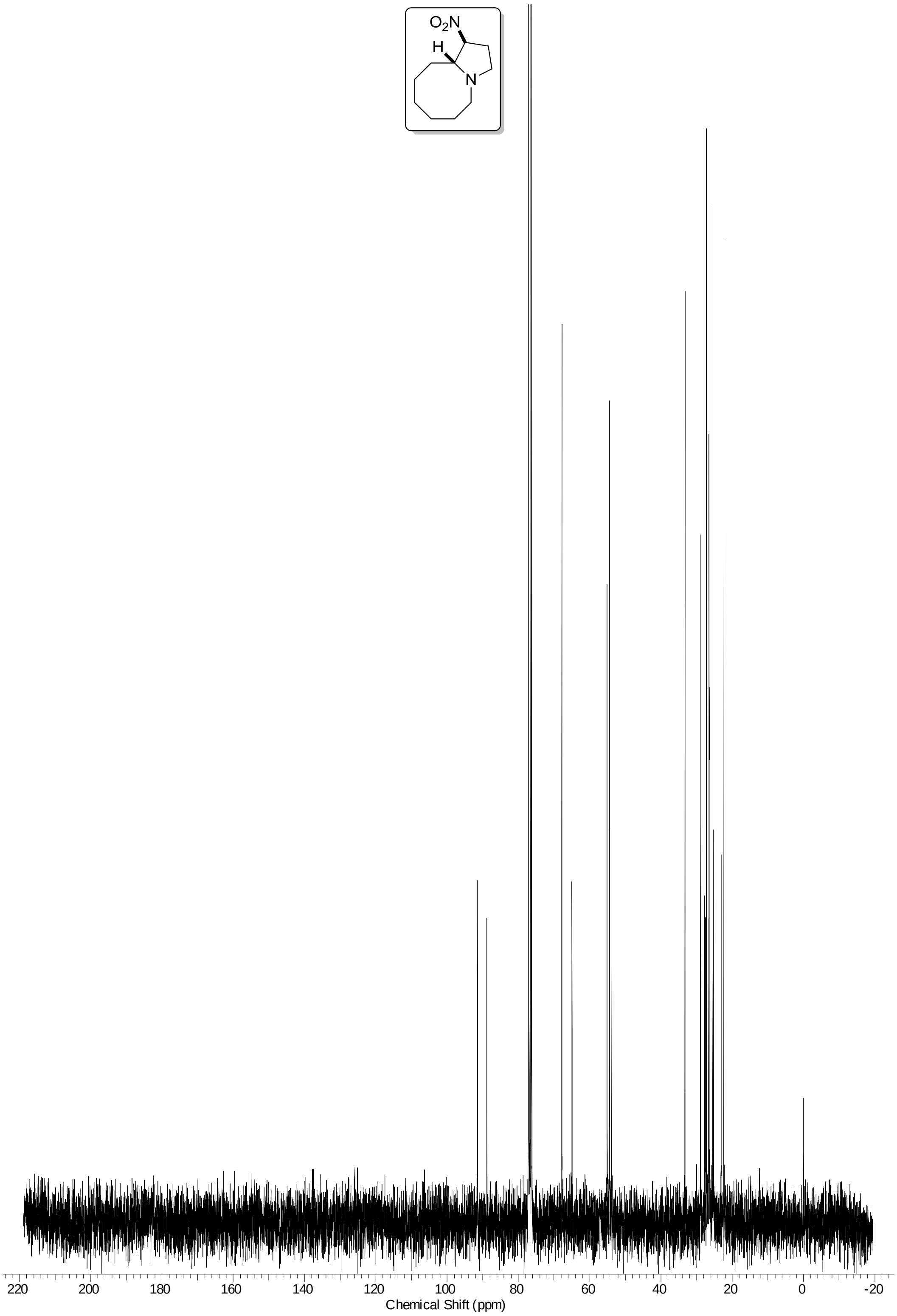

4.2.25 ^{1}HNMR spectrum of 11-nitro-1,3,4,6,11,11a-hexahydro-2*H*-pyrido[1,2-*b*]isoquinoline (5j)

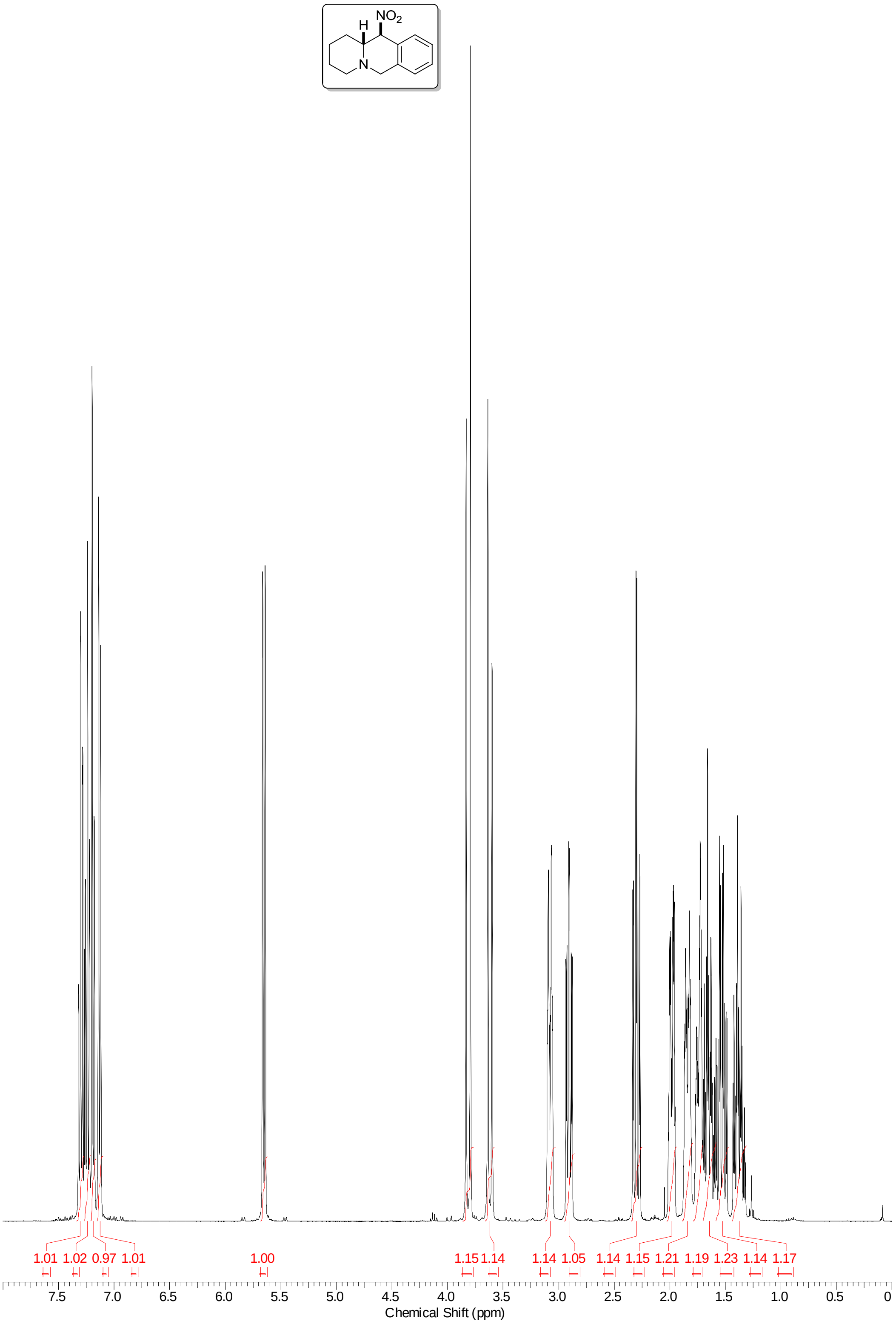

4.2.26 ^{1}HNMR spectrum of 11-nitro-1,3,4,6,11,11a-hexahydro-2*H*-pyrido[1,2-*b*]isoquinoline (5j)

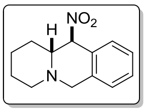

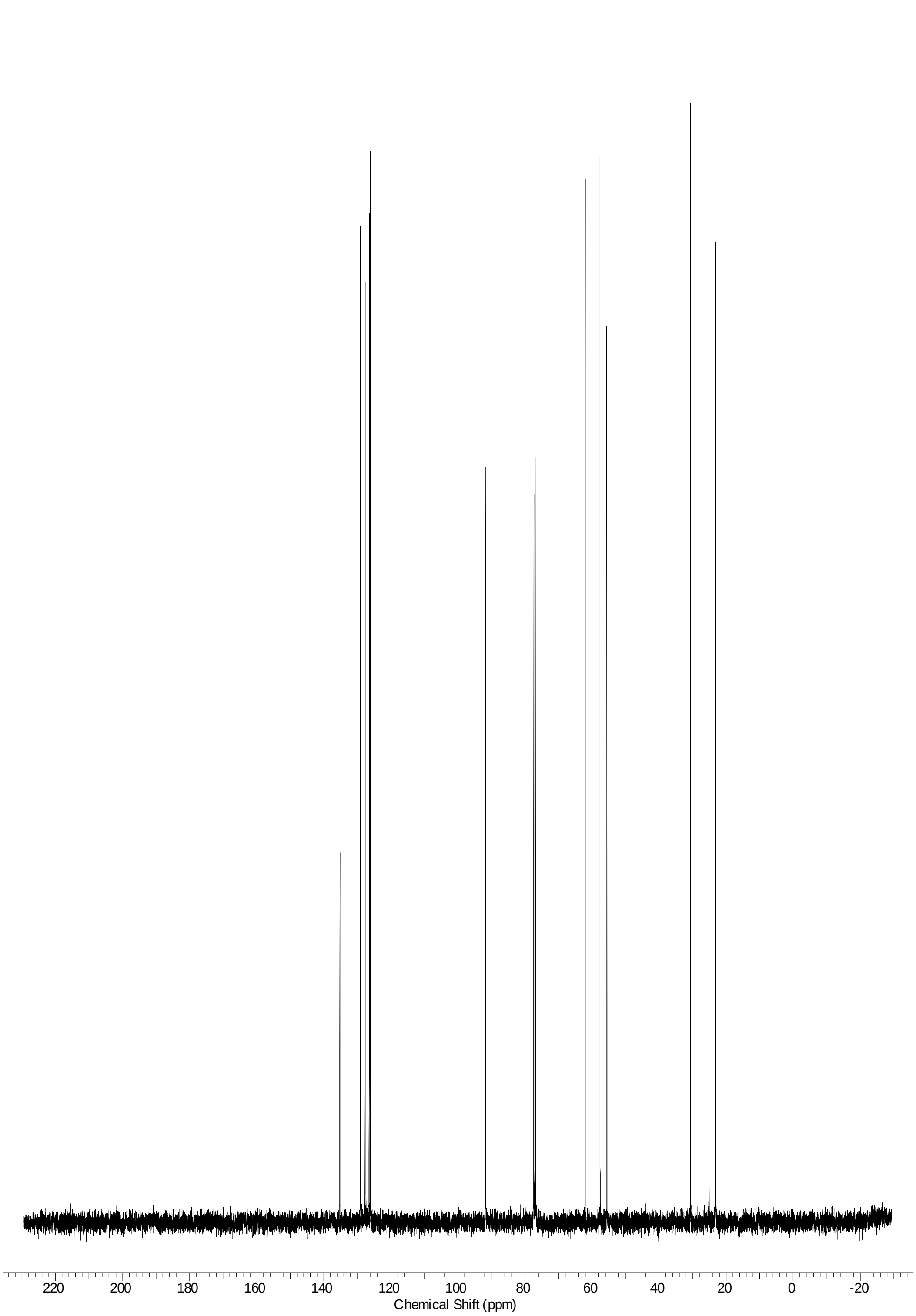

4.2.27 ^{1}HNMR spectrum of 12-nitro-5,7,8,9,10,11,11a,12-octahydroazepino[1,2-*b*]isoquinoline (5k)*

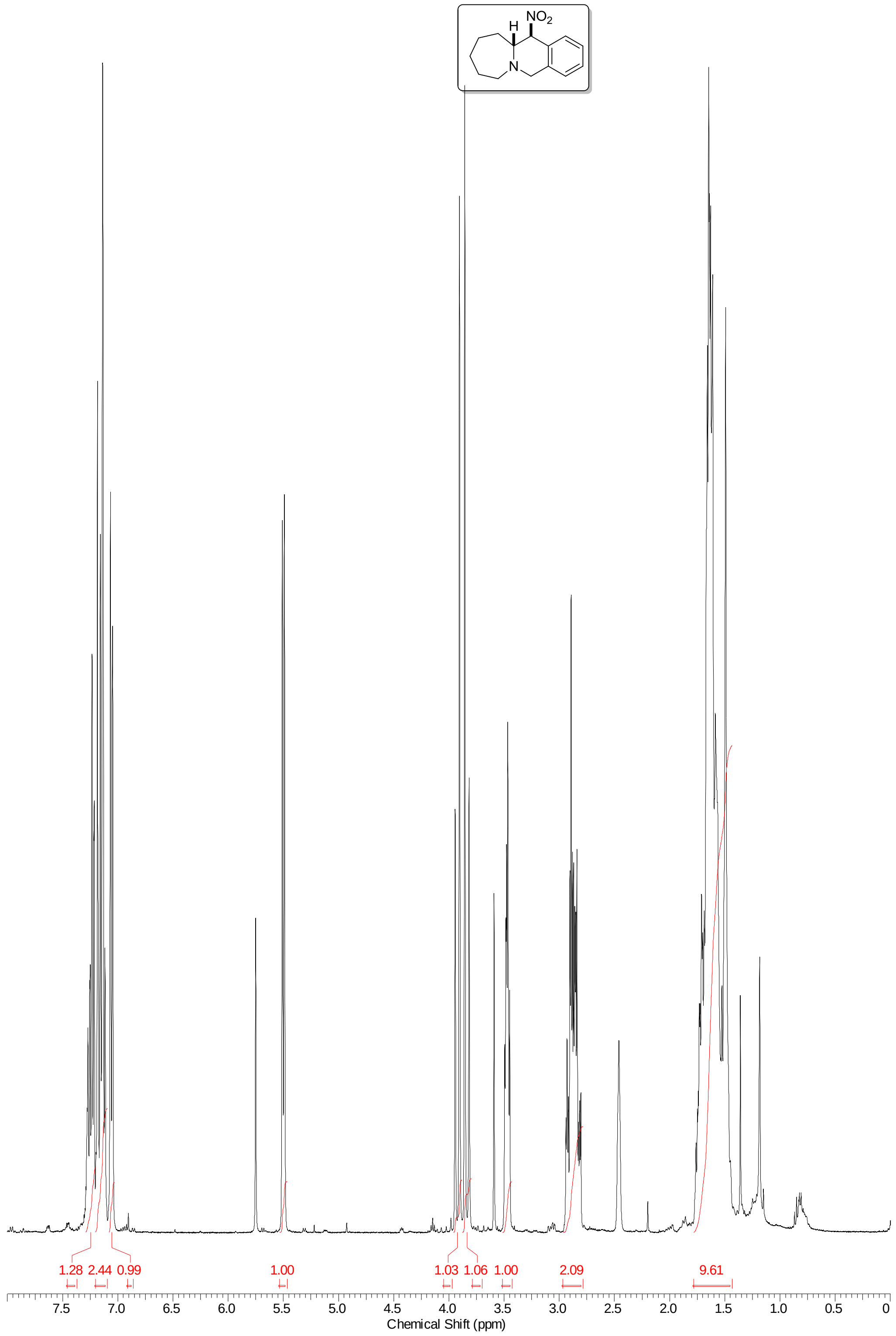

* Impurities shown are not the minor diastereomer but proposed to be inseparable fully reduced mono-cyclic nitro-amine byproduct. The yield was corrected accordingly.

4.2.28 ^{13}CNMR spectrum of 12-nitro-5,7,8,9,10,11,11a,12-octahydroazepino[1,2-*b*]isoquinoline (5k)*

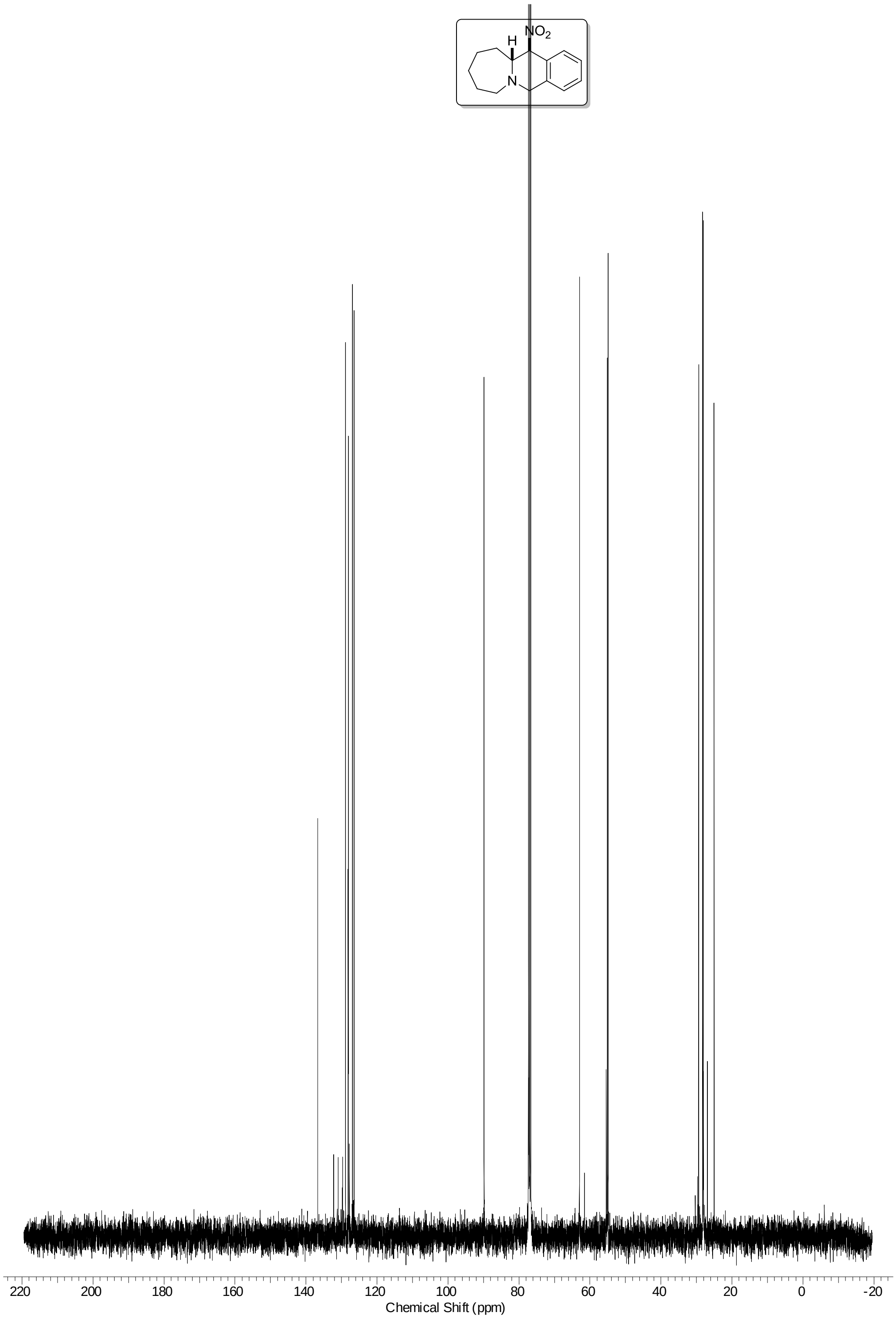

* Impurities shown are not the minor diastereomer but proposed to be inseparable fully reduced mono-cyclic nitro-amine byproduct. The yield was corrected accordingly.

4.2.29 ^{1}HNMR spectrum of 13-nitro-7,8,9,10,11,12,12a,13-octahydro-5*H*-azocino[1,2-*b*]isoquinoline (5l)*

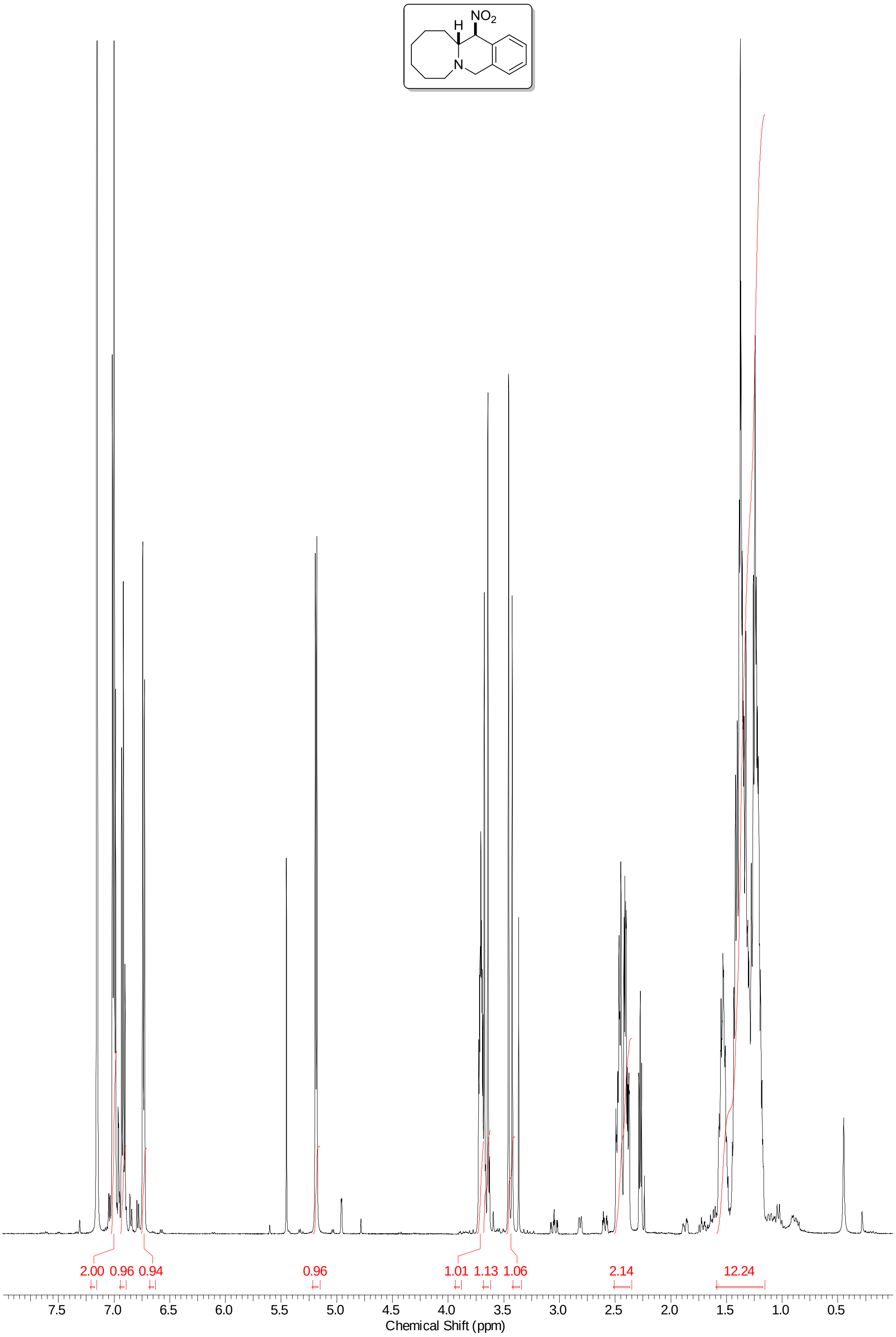

* Impurities shown are not the minor diastereomer but proposed to be inseparable fully reduced mono-cyclic nitro-amine byproduct. The yield was corrected accordingly.

4.2.30 ^{13}CNMR spectrum of 13-nitro-7,8,9,10,11,12,12a,13-octahydro-5*H*-azocino[1,2-*b*]isoquinoline (5l)*

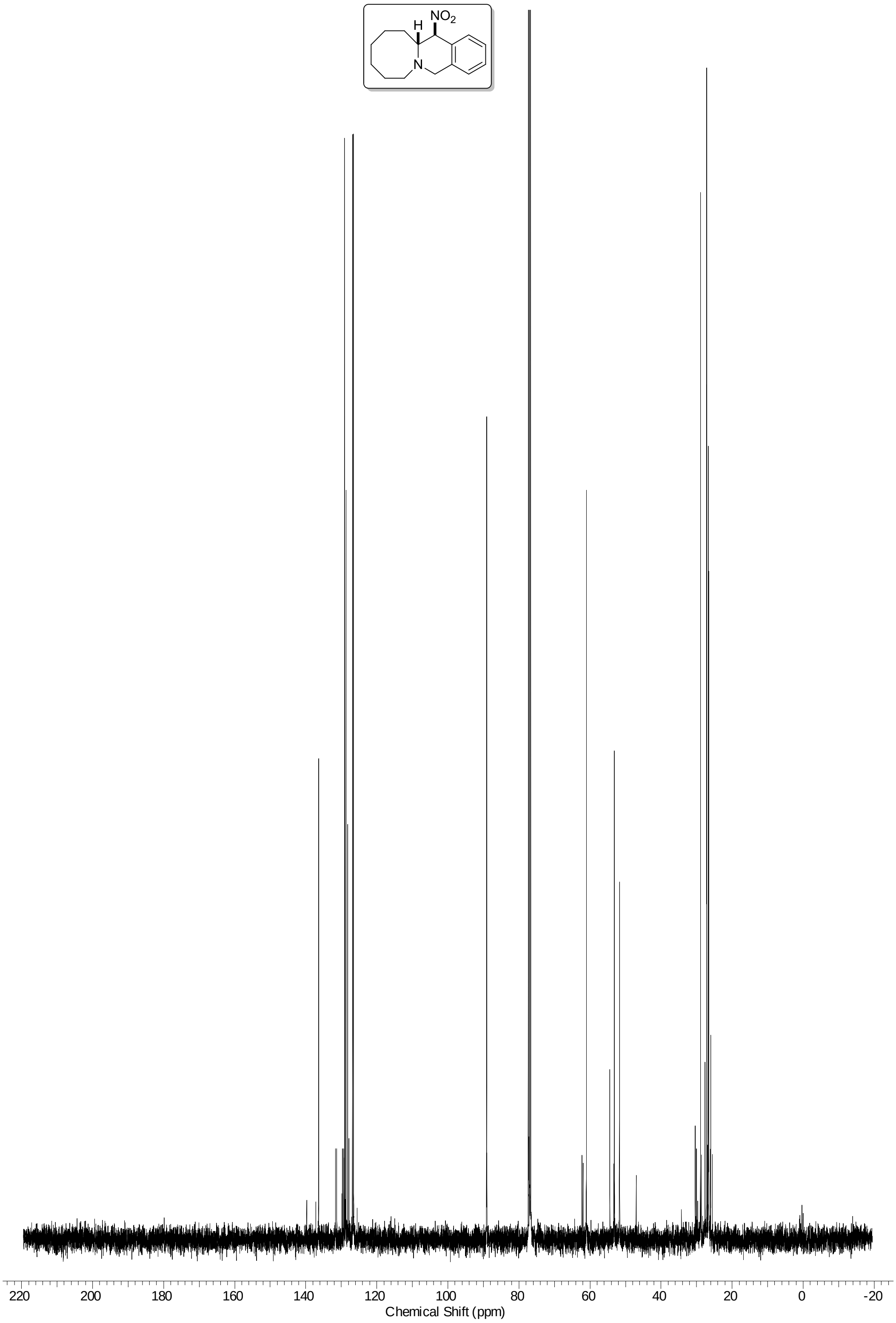

* Impurities shown are not the minor diastereomer but proposed to be inseparable fully reduced mono-cyclic nitro-amine byproduct. The yield was corrected accordingly.

4.2.31 ^{1}HNMR spectrum of 1-(4-Nitrobutyl)-2,3,4,5-tetrahydro-1*H*-azepine (7)

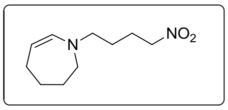

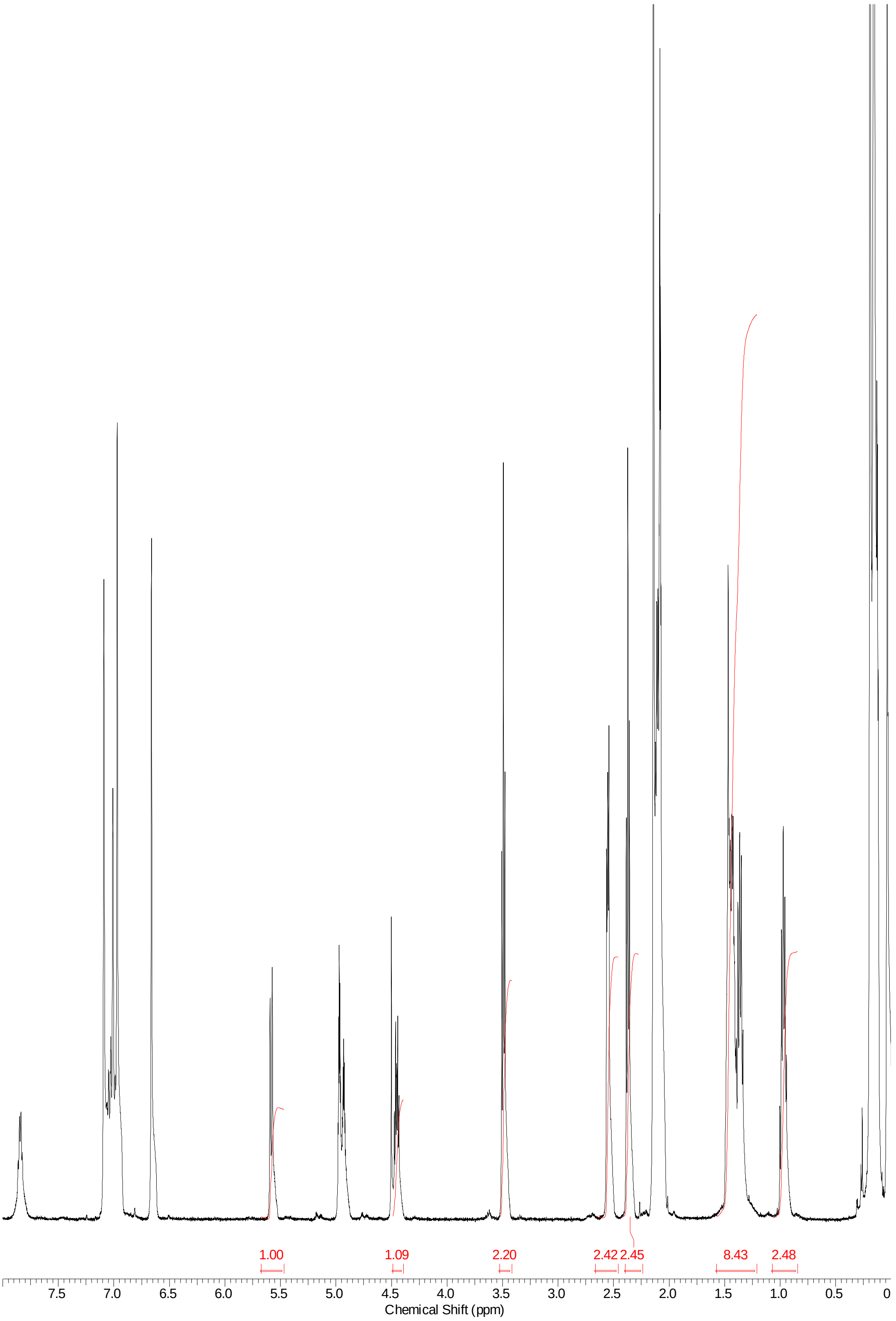

4.2.32 ^{13}CNMR spectrum of 1-(4-Nitrobutyl)-2,3,4,5-tetrahydro-1*H*-azepine (7)*

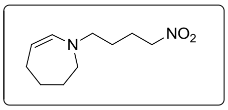

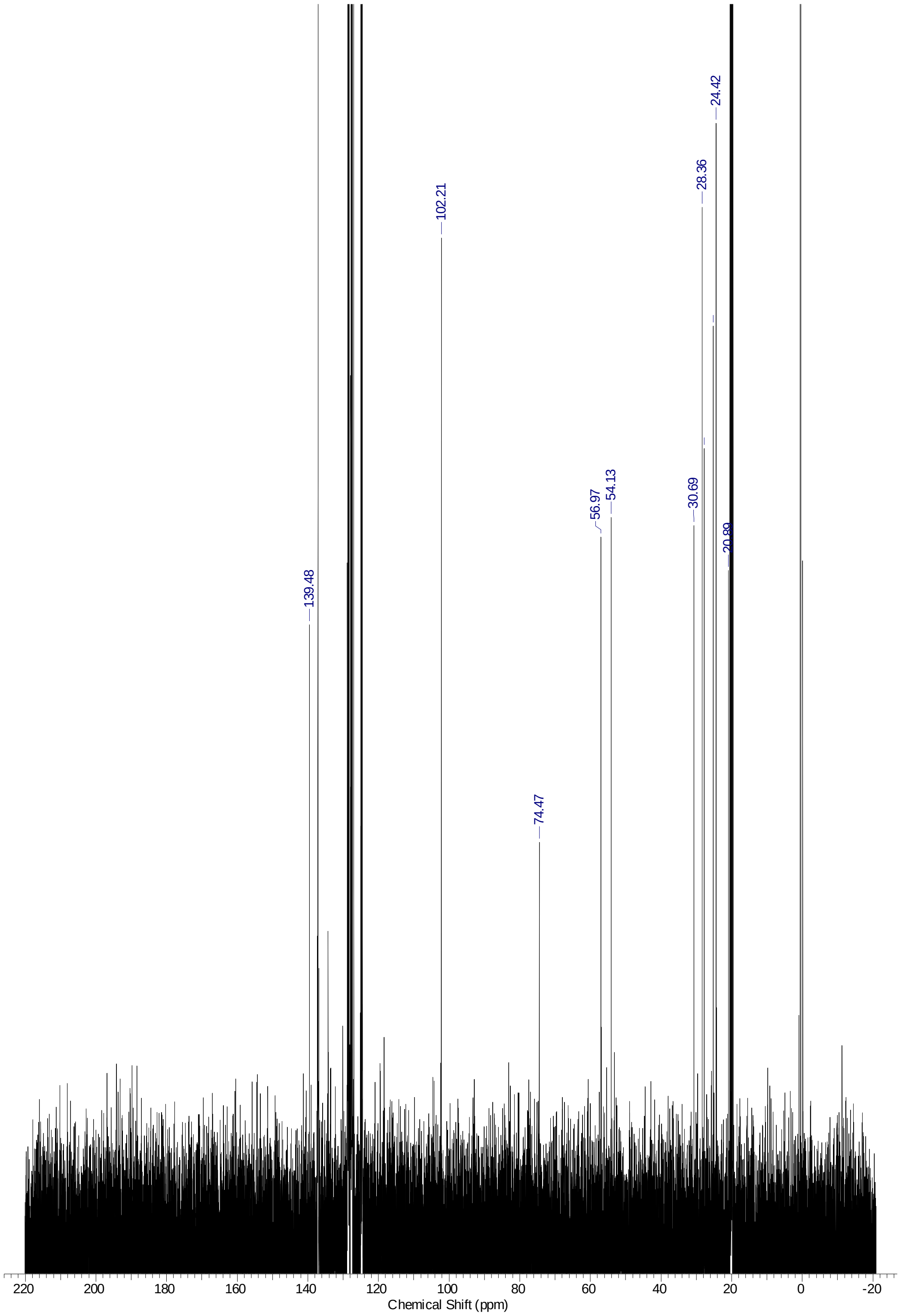

* Labeled peaks are the peaks from the title compound. Unlabeled peaks are from the mesitylene internal standard and TMDS/TMDS byproducts.

4.2.33 ^{1}HNMR spectrum of 1-(4-Nitrobutyl)-3,4,5,6-tetrahydro-2*H*-azepinium chloride (8)

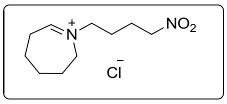

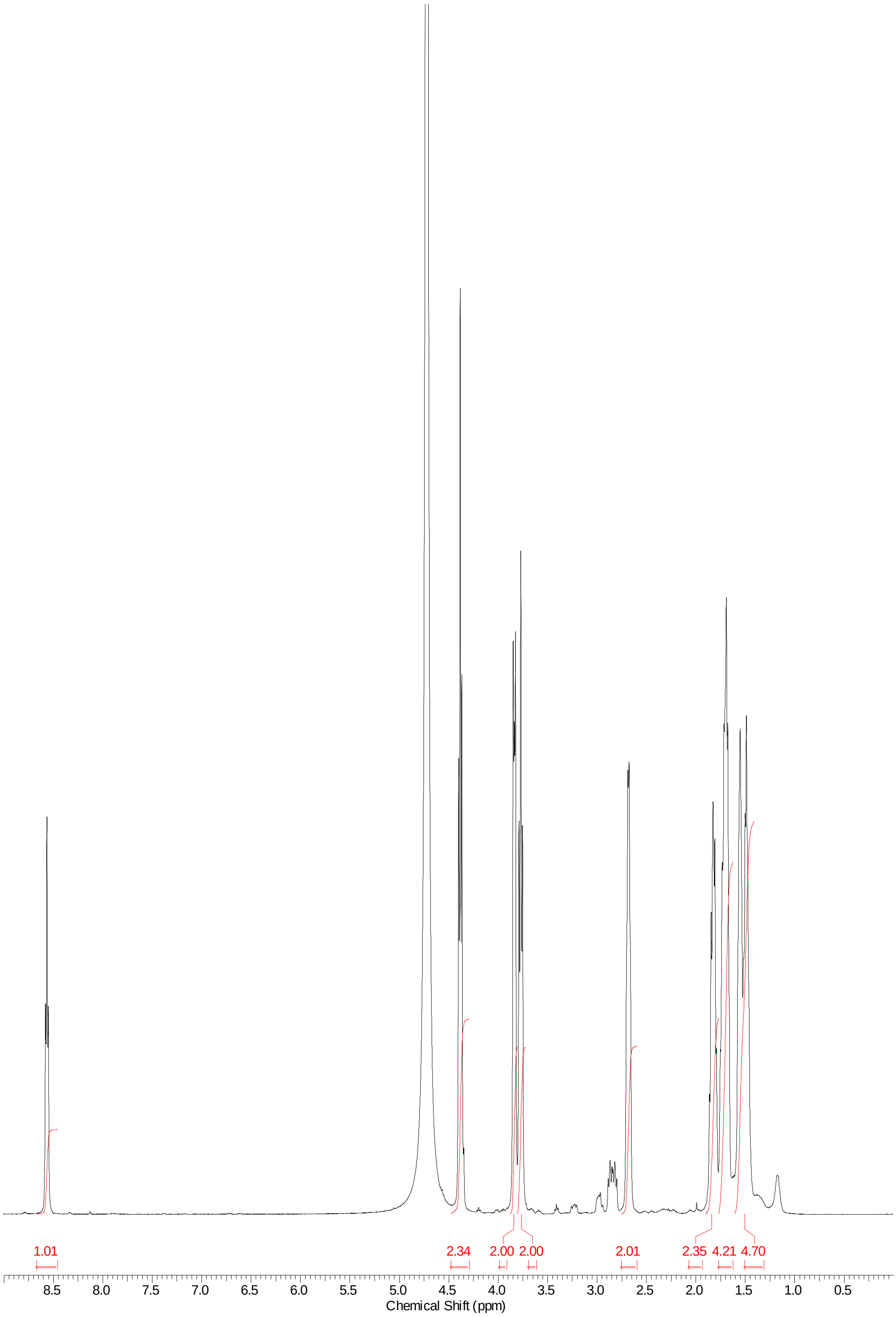

4.2.34 ^{13}CNMR spectrum of 1-(4-Nitrobutyl)-3,4,5,6-tetrahydro-2*H*-azepinium chloride (8)

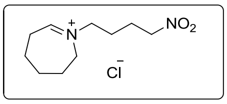

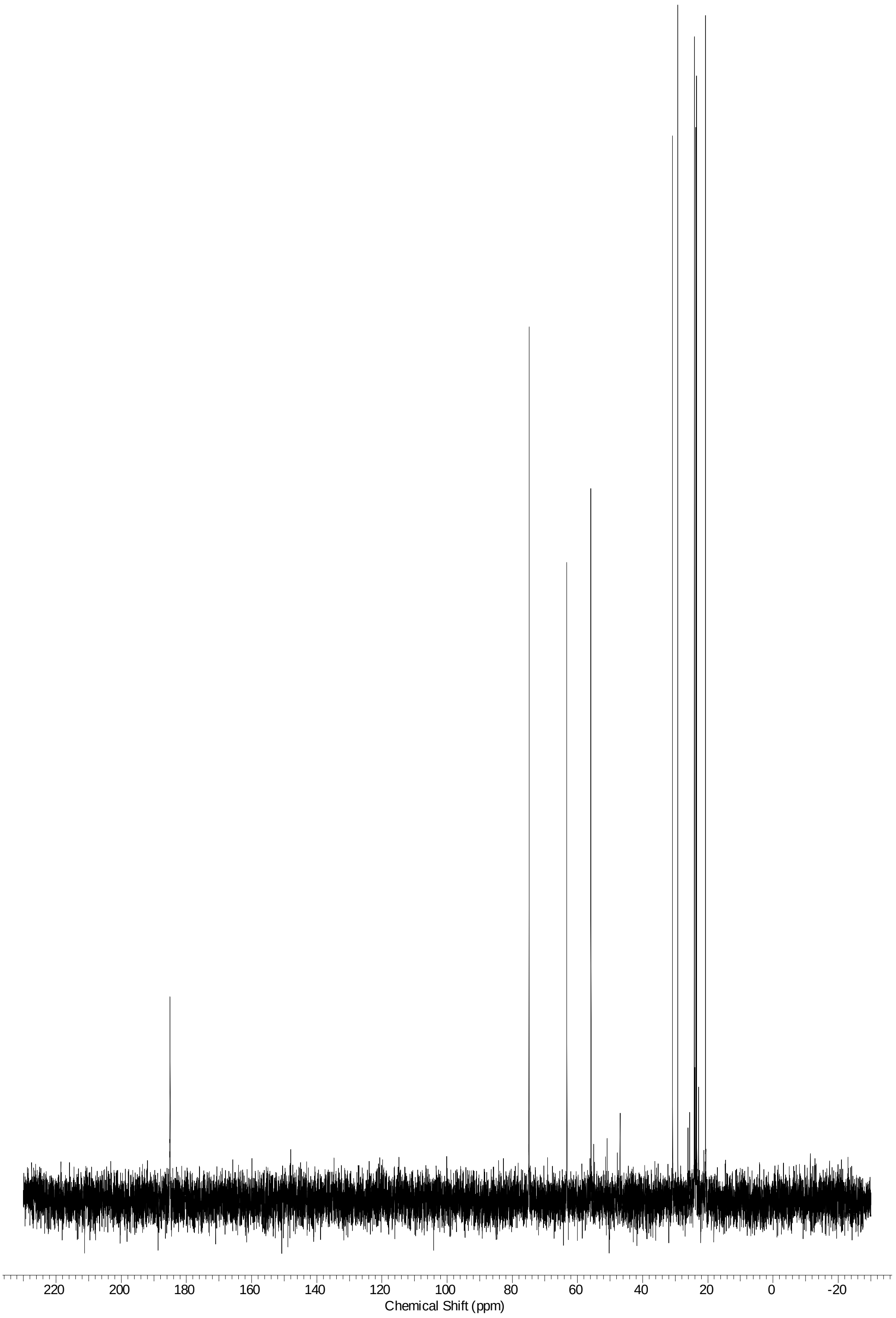

4.2.35 ^{1}HNMR spectrum of (±)-*epi*-epiquinamide

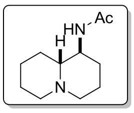

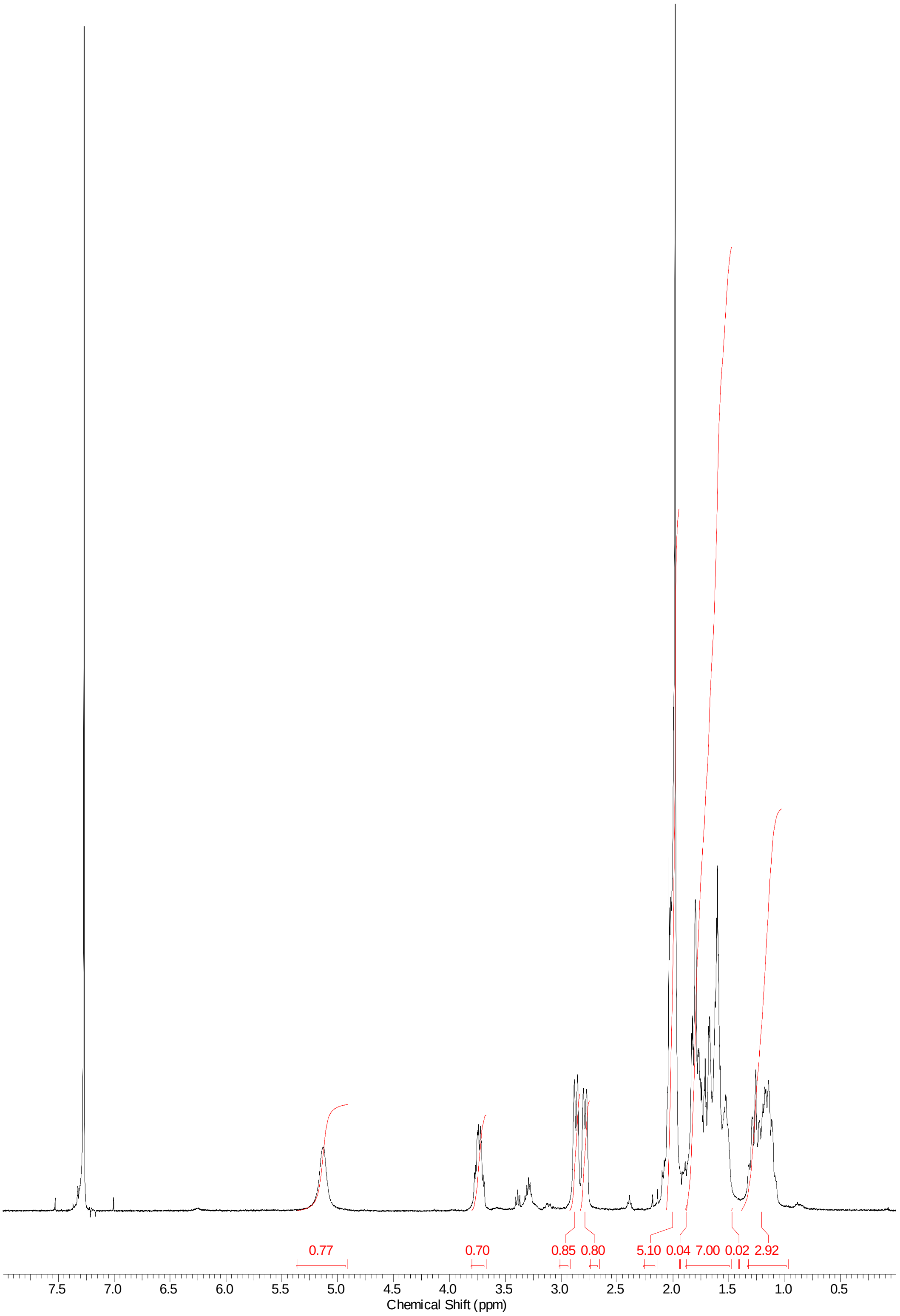

4.2.36 ^{13}CNMR spectrum of (±)-*epi*-epiquinamide

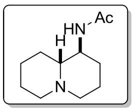

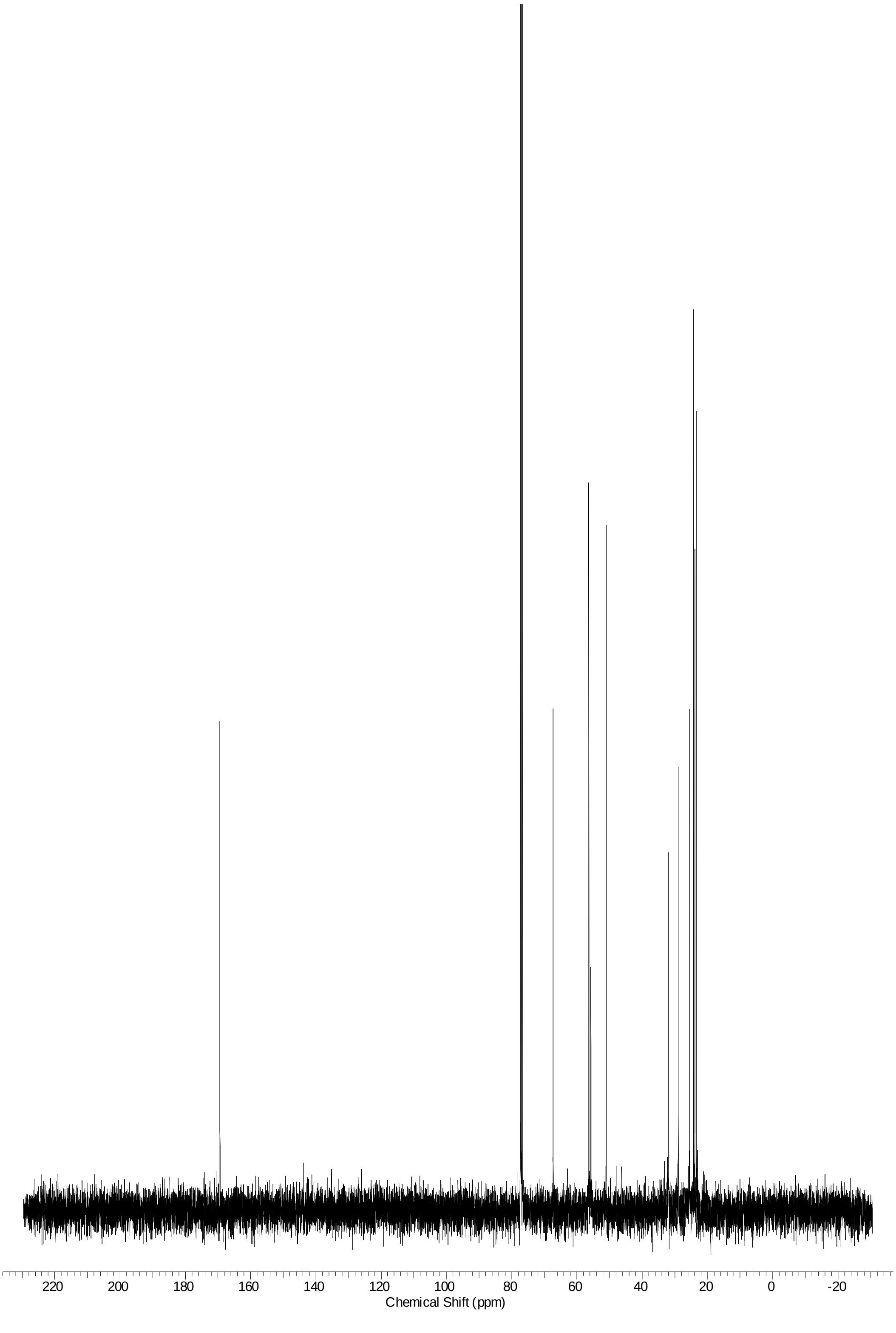

[1] Ikeda, T.; Kawamura, M.; Murase, N.; Nukui, S., Shishido, Y.; Kawai, M.; Okumura, Y. Patent: US2001/46993 A1, **2001.**
[2] Comoy, C.; Benarab, A.; Leinot, M.; Monteil, A.; Guillaumet, G. *Il Farmaco* **1999,** *54,* 791-799.
[3] Fitch, R. W.; Sturgeon, G. D.; Patel, S. R.; Spande, T. F.; Garraffo, H. M.; Daly, J. W.; Blaauw, R. H. *J. Nat. Prod.* **2009,** *72,* 243-247.
